# Five New Cytotoxic Metabolites from the Marine Fungus *Neosartorya pseudofischeri*

**DOI:** 10.3390/md14010018

**Published:** 2016-01-13

**Authors:** Wen-Jian Lan, Sheng-Jiao Fu, Meng-Yang Xu, Wan-Ling Liang, Chi-Keung Lam, Guo-Hua Zhong, Jun Xu, De-Po Yang, Hou-Jin Li

**Affiliations:** 1 School of Pharmaceutical Sciences, Sun Yat-sen University, Guangzhou 510006, China; lanwj@mail.sysu.edu.cn (W.-J.L.); xumy3@mail2.sysu.edu.cn (M.-Y.X.); natprodlwl@gmail.com (W.-L.L.); junxu@biochemomes.com (J.X.); lssydp@mail.sysu.edu.cn (D.-P.Y.); 2 Guangdong Technology Research Center for Advanced Chinese Medicine, Guangzhou 510006, China; 3 Lab of Insect Toxicology, South China Agricultural University, Guangzhou 510642, China; marnatprod2015@yeah.net (S.-J.F.); guohuazhong@scau.edu.cn (G.-H.Z.); 4 School of Chemistry and Chemical Engineering, Sun Yat-sen University, Guangzhou 510275, China; cklam@mail.sysu.edu.cn

**Keywords:** marine fungus, *Neosartorya pseudofischeri*, phenylpyropene, pyripyropene, sesquiterpene, cytotoxic activity

## Abstract

The marine fungus *Neosartorya pseudofischeri* was isolated from *Acanthaster planci* from the South China Sea. In a preliminary bioactivity screening, the crude methanol extract of the fungal mycelia showed significant inhibitory activity against the Sf9 cell line from the fall armyworm *Spodoptera frugiperda*. Five novel compounds, including 5-olefin phenylpyropene A (**1**), 13-dehydroxylpyripyropene A (**4**), deacetylsesquiterpene (**7**), 5-formyl-6-hydroxy-8-isopropyl-2- naphthoic acid (**9**) and 6,8-dihydroxy-3-((1*E*,3*E*)-penta-1,3-dien-1-yl)isochroman-1-one (**10**), together with eleven known compounds, phenylpyropene A (**2**) and C (**3**), pyripyropene A (**5**), 7-deacetylpyripyropene A (**6**), (1*S*,2*R*,4a*R*,5*R*,8*R*,8a*R*)-1,8a-dihydroxy-2-acetoxy-3,8-dimethyl-5- (prop-1-en-2-yl)-1,2,4a, 5,6,7,8,8a-octahydronaphthalene (**8**), isochaetominine C (**11**), trichodermamide A (**12**), indolyl-3-acetic acid methyl ester (**13**), 1-acetyl-β-carboline (**14**), 1,2,3,4-tetrahydro-6-hydroxyl-2-methyl-l,3,4-trioxopyrazino[l,2-a]-indole (**15**) and fumiquinazoline F (**16**), were obtained. The structures of these compounds were determined mainly by MS and NMR data. The absolute configuration of **9** was assigned by the single-crystal X-ray diffraction studies. Compounds **1**–**11** and **15** showed significant cytotoxicity against the Sf9 cells from *S. frugiperda*.

## 1. Introduction

The fall armyworm *Spodoptera frugiperda* is a pest feeding on the gramineous plants occurring worldwide and causing serious damage to several economically-important crops, such as the maize, sorghum, sugarcane, as well as the cotton, cruciferous and Cucurbitaceae plants. Although other alternative methods have been investigated on the biological control, as well as the development of resistant transgenic plants, the control of *S. frugiperda* still relies mainly on the use of chemical pesticides, easily inducing resistance and causing severe damage to the environment and consumers. However, some natural products with higher selectivity and safety become an important potential source of new biopesticides for the control of *S. frugiperda* [1,2,3,4,5]. Sf9 cells are clonal isolates of *S. frugiperda* Sf21 cells (IPLB-SF21-AE) [6]. A cultured Sf9 cell line is commonly used for primary screening to determine insecticidal activity [7] and in biomedical research for the purpose of recombinant protein expression using insect-specific viruses called baculoviruses [8].

**Figure 1:**
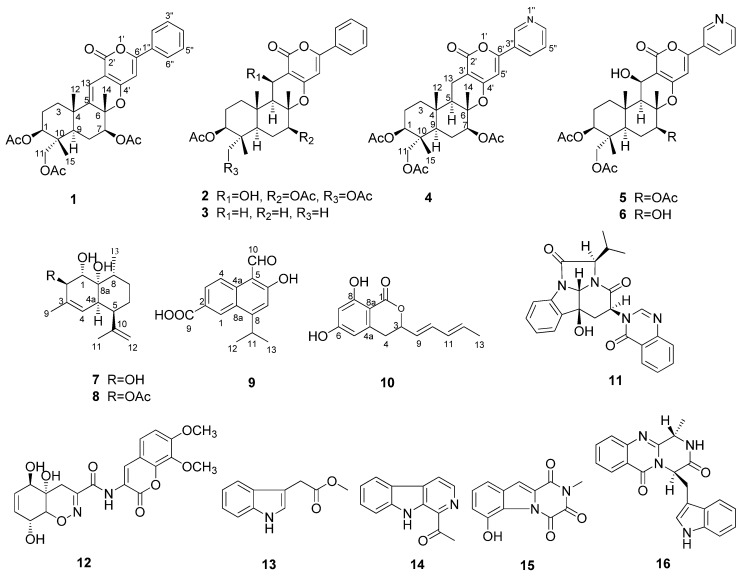
Structures of Compounds **1**–**16**.

In recent years, we conducted research on the metabolites of marine fungi and obtained a series of novel and/or bioactive metabolites [9,10,11,12,13]. The marine fungus *Neosartorya pseudofischeri* was isolated from the inner tissue of a starfish *Acanthaster planci* collected from the South China Sea. In the previous metabolites study, we obtained three novel compounds, neosartins A–C, as well as a series of known gliotoxin analogues and diketopiperazines with potent antitumor and antibacterial activities from the culture broth extract of GlyPY (glycerol-peptone-yeast extract) and GluPY (glucose-peptone-yeast extract) media [14]. During our insecticidal activity screening against the fall armyworm *S. frugiperda*, we found that the crude methanol extract of the mycelia of *N. pseudofischeri* cultivated in GluPY showed a 65% cell growth inhibition rate against the Sf9 cell line from *S. frugiperda* at a concentration of 50 mg/L. The bioactivity-guided metabolites isolation afforded five novel compounds, including 5-olefin phenylpyropene A (**1**), 13-dehydroxyl pyripyropene A (**4**), deacetylsesquiterpene (**7**), 5-formyl-6-hydroxy-8-isopropyl-2-naphthoic acid (**9**) and 6,8-dihydroxy-3-((1*E*,3*E*)-penta-1,3-dien-1-yl)isochroman-1-one (**10**), together with eleven known compounds, phenylpyropene A (**2**) and C (**3**), pyripyropene A (**5**), 7-deacetylpyripyropene A (**6**), (1*S*,2*R*,4a*R*,5*R*,8*R*,8a*R*)-1,8a-dihydroxy-2-acetoxy-3,8-dimethyl-5-(prop-1-en-2-yl)-1,2,4a,5,6,7,8,8a-octahydronaphthalene (**8**), isochaetominine C (**11**), trichodermamide A (**12**), indolyl-3-acetic acid methyl ester (**13**), 1-acetyl-β-carboline (**14**), 1,2,3,4-tetrahydro-6-hydroxyl-2-methyl-l,3,4- trioxopyrazino[l,2-a]-indole (**15**) and fumiquinazoline F (**16**) (Figure 1). Compounds **1**–**11** and **15** showed significant cytotoxicity against the Sf9 cells from *S. frugiperda*. Herein, we report the isolation, structure elucidation and the bioactivity of these compounds.

## 2. Results and Discussion

### 2.1. Structural Elucidation

**Figure 2:**
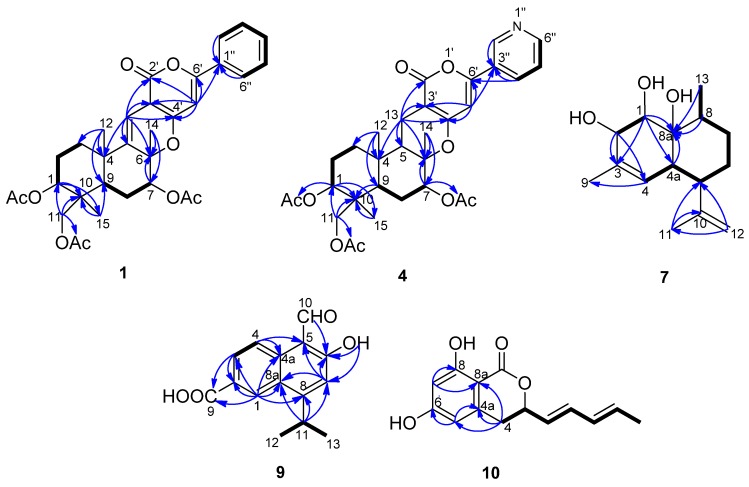
^1^H–^1^H COSY (bold lines) and the main HMBC (arrows) correlations of Compounds **1**, **4**, **7**, **9** and **10**.

5-Olefin phenylpyropene A (**1**) was isolated as a yellowish oil. The molecular formula was established as C_32_H_36_O_9_ based on the analysis of ^13^C-NMR data and HR(+)ESIMS *m/z* 565.2448 [M + H]^+^ (calcd. for C_32_H_36_O_9_, 565.2432), implying fifteen degrees of unsaturation (Supplementary Figure S1). The IR spectrum indicated the presence of a carbonyl group (1680 cm^−1^) and a benzene ring (3073, 1574 and 1507 cm^−1^). UV maxima at 232, 278 and 322 nm supported the long conjugated system containing a benzene ring. The ^13^C-NMR and DEPT spectra displayed six methyls, four methylenes, ten methines and twelve quaternary carbons. The diagnostic aryl protons at *δ*_H_ 7.81 (m, 2H) and 7.44 (m, 3H) revealed a monosubstituted benzene ring. Two alkenyl protons at *δ*_H_ 6.46 (s) and 6.36 (s) indicated there were two trisubstituted double bonds in the molecule. Three methyl group singlets at *δ*_H_ 2.16, 2.10 and 2.04 showed HMBC correlations with the carbonyl groups at *δ*_C_ 169.9, 171.1 and 170.4, respectively, confirming there are three acyloxy groups. The other three methyl group singlets at *δ*_H_ 1.57, 1.25 and 0.87 are connected with the quaternary carbons at *δ*_C_ 21.2, 24.2 and 13.3, respectively. In the ^1^H–^1^H COSY spectrum, the cross peaks of H-1/H-2, H-2/H-3, H-7/H-8, H-8/H-9, H-2′′(H-6′′)/H-3′′(H-5′′) and H-3′′(H-5′′)/H-4′′ revealed the partial structure of –CHCH_2_CH_2_–, –CHCH_2_CH– and –CHCHCHCHCH– in the molecule (Figure 2). The key HMBC correlations of H-1/C-10, H-11/C-10, H-15/C-10, H-12/C-3, H-12/C-4, H-12/C-5, H-13/C-4, H-13/C-5, H-13/C-6, H-7/C-6, H-14/C-6, H-12/C-9 and H-15/C-9 deduced the sesquiterpene moiety. The cross peaks of H-13 to C-2′, C-3′ and C-4′, H-5′ to C-4′, C-6′ and C-1′′ in the HMBC spectrum confirmed the connectivity of the sesquiterpene fragment, α-pyrone and the monosubstituted phenyl ring (Table 1 and Supplementary Figures S2–S7).

**Table 1:** ^1^H and ^13^C-NMR data of **1**, **4** and **6** at 400/100 MHz, respectively, δ in ppm.

Position	1 ^a^		4 ^a^		6 ^b^	
	*δ*_C_	*δ*_H_ (*J* in Hz)	*δ*_C_	*δ*_H_ (*J* in Hz)	*δ*_C_	*δ*_H_ (*J* in Hz)
1	73.3, CH	4.79, dd (11.6, 4.4)	73.5, CH	4.79, dd (11.6, 4.8)	74.4, CH	4.79, dd (11.2, 5.6)
2	23.2, CH_2_	1.98, m; 1.75, m	22.8, CH_2_	1.88, m; 1.70, m	23.6, CH_2_	1.84, m; 1.88, m
3	35.4, CH_2_	2.08, m; 1.62, m	36.6, CH_2_	1.83, m; 1.20, ddd (13.2, 13.2, 3.2)	36.8, CH_2_	1.43, td (12.8, 4.8); 2.15, td (12.8, 4.8)
4	38.7, C		36.6, C		38.8, C	
5	143.7, C		50.3, CH	1.60, dd (12.4, 4.8)	55.0, CH	1.66, d (3.6)
6	83.5, C		81.9, C		86.2, C	
7	77.7, CH	5.22, d (12.0, 5.2)	77.5, CH	5.02, dd (11.6, 4.8)	77.6, CH	4.97, dd (12.4, 5.2)
8	24.3, CH_2_	1.81, m; 1.63, m	24.9, CH_2_	1.78, m; 1.50, m	29.0, CH_2_	1.60, d (12.4); 1.82, m
9	41.0, CH	1.73, m	45.1, CH	1.66, d (12.0)	46.2, CH	1.53, d (3.6)
10	40.5, C		40.2, C		41.3, C	
11	64.6, CH_2_	3.78, d (12.0); 3.74, d (12.0)	64.7, CH_2_	3.78, d (12.0); 3.73, d (12.0)	64.6, CH_2_	3.83, d (11.6); 3.77, d (11.6)
12	24.2, CH_3_	1.25, s	15.5, CH_3_	1.00, s	17.9, CH_3_	1.48, s
13	111.4, C	6.36, s	16.9, CH_2_	2.57, dd (17.2, 4.8); 2.33, dd (17.2, 12.4)	60.6, CH	4.95, d (3.6)
14	21.2, CH_3_	1.57, s	15.4, CH_3_	1.32, s	15.9, CH_3_	1.76, s
15	13.3, CH_3_	0.87, s	13.3, CH_3_	0.86, s	13.3, CH_3_	0.92, s
2′	162.1, C		163.7, C		163.5, C	
3′	100.3, C		99.8, C		100.1, C	
4′	160.2, C		162.2, C		157.9, C	
5′	97.4, CH	6.46, s	99.2, CH	6.43, s	147.6, CH	
6′	154.9, C		155.9, C		128.4, C	
1′′	131.0, C					
2′′	125.6, CH	7.81, m	146.7, CH	8.99, s	147.6, CH	9.07, dd (2.4, 0.8)
3′′	128.9, CH	7.44, m	127.3, C		127.5, C	
4′′	131.0, CH	7.44, m	132.8, CH	8.09, brd (8.0)	133.6, CH	8.22, ddd (8.4, 2.4, 1.6)
5′′	128.9, CH	7.44, m	123.6, CH	7.39, dd (8.0, 4.8)	124.6, CH	7.53, ddd (8.4, 4.8, 0.8)
6′′	125.6, CH	7.81, m	151.2, CH	8.66, d (4.8)	152.3, CH	8.69, dd (4.8, 1.6)
1-OCO*CH_3_*	21.1, CH_3_	2.04, s	21.1, CH_3_	2.04, s	21.0, CH_3_	2.00, s
1-O*CO*CH_3_	170.0, C		170.5, C		170.6, C	
7-OCO*CH_3_*	21.1, CH_3_	2.16, s	21.3, CH_3_	2.15, s		
7-O*CO*CH_3_	169.9, C		170.1, C			
11-OCO*CH_3_*	20.8, CH_3_	2.10, s	20.8, CH_3_	2.11, s	20.7, CH_3_	1.99, s
11-O*CO*CH_3_	171.1, C		171.0, C		170.8, C	
13-OH						4.28, brs
7-OH						4.10, brs

^a 1^H and ^13^C-NMR data were measured in CDCl_3_; ^b 1^H and ^13^C-NMR data were measured in acetone-*d*_6_.

**Figure 3:**
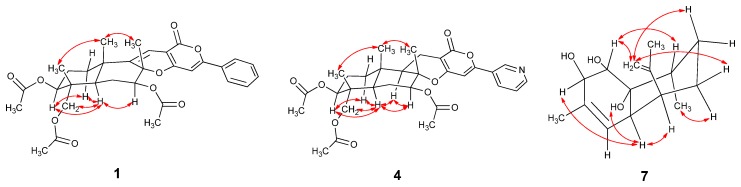
Key NOESY correlations of Compounds **1**, **4** and **7**.

The relative configuration of **1** was determined by the analysis of the NOESY spectrum. The NOE correlations between H-12/H-14 and H-12/H-15, H-1/H-9, H-7/H-9 and H-7/H-11 were observed. Consequently, H-12, H-14 and H-15 are β-oriented, while H-1, H-7 and H-9 are α-oriented (Figure 3).

Compounds **2** and **3** were identified as phenylpyripyropene A and C [15,16], by comparing their NMR data to the reference values. The double bond between the C-5 and C-13 positions in **1** was replaced by the saturated carbon-carbon bond in **2**. Compounds **1** and **2** have triacyloxy groups at the C-1, C-7 and C-11 positions; however, Compound **3** has only one acyloxy group at the C-1 position (Supplementary Figures S8–S11).

13-Dehydroxylpyripyropene A (**4**) was obtained as a white powder. It showed a molecular formula of C_31_H_37_NO_9_ determined by the ^13^C-NMR data and HR(+)ESIMS peak at *m*/*z* 568.2549 [M + H]^+^ (calcd. for C_31_H_37_NO_9_, 568.2541) (Supplementary Figure S12). The ^13^C-NMR and DEPT spectra (Table 1) displayed six methyls, five methylenes, nine methines and eleven quaternary carbons. By comparing the NMR data with pyripyropene A (**5**) (Supplementary Figures S13–S20) [17], a quick identification was made revealing that C-13 of **4** was a methylene (*δ*_C_ 16.9, *δ*_H_ 2.57, 2.33), corresponding to one methine group (*δ*_C_ 60.1, *δ*_H_ 4.99) connected to one hydroxyl group in pyripyropene A (Figure 2). The relative configuration of **4** was determined on the basis of NOESY data. The NOE correlations of H-12/H-15, H-14/H-15 supported these methyl groups on the β-position of the ring system; while those correlations of H-1/H-9, H-5/H-7, H-5/H-9, H-7/H-9 and H-9/H-11 were used to place these protons on the α-position (Figure 3).

Compound **6** was elucidated as 7-deacetyl pyripyropene A (Supplementary Figures S21 and S22). **6** was once obtained by hydrolysis of pyripyropene A with 1,8-diazabicyclo[5,4,0]undec-7-ene (DBU) in 80% methanol [18]. However, this is the first report including the detailed NMR data.

Deacetylsesquiterpene (**7**) was isolated as a yellowish oil. The molecular formula was determined as C_15_H_24_O_3_ from the ^13^C-NMR data and the HR(+)ESIMS peak at *m*/*z* 275.1603 [M + Na]^+^ (calcd. for C_15_H_24_O_3_Na, 275.1618), indicating four degrees of unsaturation (Supplementary Figure S23). The strong IR absorption at 3443 cm^−1^ indicated the presence of the hydroxyl groups. The ^13^C-NMR and DEPT spectra displayed three methyls, three methylenes, six methines and three quaternary carbons (Table 2). The quaternary carbon (*δ*_C_ 132.3) and the methine (*δ*_C_ 124.4, *δ*_H_ 5.28) formed a trisubstituted double bond. The quaternary carbon (*δ*_C_ 147.2) and the methylene (*δ*_C_ 111.0, *δ*_H_ 4.94 and 4.72) constructed a terminal double bond. Thus, Compound **7** must be bicyclic to account for the four double bond equivalents required by the molecular formula. The ^1^H–^1^H COSY cross peaks of H-1/H-2, H-4/H-4a, H-4a/H-5, H-5/H-6, H-6/H-7, H-7/H-8 and H-8/H-13 established two partial structures, –CHCH– and –CHCHCHCH_2_CH_2_CHCH_3_ (Figure 2). The HMBC correlations of H-1/C-8, H-2/C-3, H-5/C-10, H-8/C-8a, H-9/C-3 and H-11/C-10 established the skeleton; three hydroxyl groups were attached to C-1 (*δ*_C_ 74.6), C-2 (*δ*_C_ 74.2) and C-8a (*δ*_C_ 73.8). The NOESY correlations of H-1/H-12, H-7a/H-12, H-6e/H-12, H-2/H-4a, H-4a/H-5, H-4a/8a-OH and H-6a/H-13 revealing 1-OH, 8a-OH, H-5 and H-13 were α-oriented; however, 2-OH was β-oriented (Figure 3 and Supplementary Figures S24–S29).

**Table 2:** ^1^H and ^13^C-NMR data of **7**, **9** and **10**, δ in ppm.

Position	7 ^a^		9 ^b^		10 ^c^	
	δ_C_, Type	δ_H_, Mult., (*J* in Hz)	δ_C_, Type	δ_H_, Mult., (*J* in Hz)	δ_C_, Type	δ_H_, Mult., (*J* in Hz)
1	74.6, CH	3.98, d (1.2)	127.9, CH	8.92, d (1.5)	169.1, C	
2	74.2, CH	4.05, s	137.4, C			
3	132.3, C		129.2, CH	8.22, dd (9.0,1.5)	78.4, CH	5.13, ddd (10.0, 6.8, 4.0)
4	124.4, CH	5.28, s	121.6, CH	8.77, d (9.0)	32.5, CH_2_	3.00, dd (16.4, 4.0); 2.93, dd (16.4, 10.0)
4a	38.1, CH	2.67, s	127.3, C		141.7, C	
5	41.4, CH	2.38, brd (12.0)	111.4, C		107.1, CH	6.24, d (2.0)
6	25.9, CH_2_	1.56, m; 1.33, m	167.1, C		163.4, C	
7	30.7, CH_2_	1.48, m; 1.40, m	117.0, CH	7.21, s	101.0, CH	6.18, d (2.0)
8	31.2, CH	1.99, m	159.5, C		164.8, C	
8a	73.8, C		126.3, C		100.1, C	
9	20.1, CH_3_	1.78, s	167.6, C		127.0, CH	5.71, dd (15.2, 6.8)
10	147.2, C		195.3, CH	11.42, brs	133.3, CH	6.35, dd (15.2, 10.0)
11	22.4, CH_3_	1.74, brs	30.1, CH	3.90, heptet (7.0)	130.4, CH	6.10, ddd (15.2, 10.0, 1.2)
12	111.0, CH_2_	4.94, q (1.2 ); 4.72, s	23.4, CH_3_	1.45, d (7.0)	131.7, CH	5.81, dq (15.2, 6.8)
13	15.0, CH_3_	0.93, d (6.8)	23.4, CH_3_	1.45, d (7.0)	18.0, CH_3_	1.74, dd (6.8, 1.2)
1-OH		2.00, s				
2-OH		2.00, s				
2-O*CO*CH_3_						
2-OCO*CH_3_*						
6-OH				13.42, s		11.06, brs
8-OH						11.06, brs
8a-OH		2.00, s				
CO*OH*				10.94, brs		

^a 1^H and ^13^C-NMR data were measured at 400/100 MHz, in CDCl_3_; ^b 1^H and ^13^C-NMR data were measured at 500/125 MHz, in acetone-*d*_6_; ^c 1^H and ^13^C-NMR data were measured at 400/100 MHz, in DMSO-*d*_6_.

**Figure 4:**
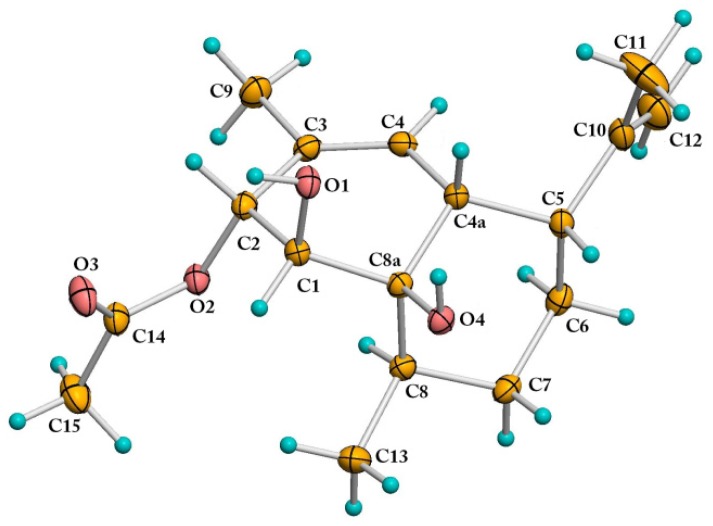
ORTEP (Oak Ridge Thermal Ellipsoid Plot) drawing of Compound **8**.

The formula of Compound **8** was identified as C_17_H_26_O_4_, based on the analysis of ^13^C-NMR data and HREIMS peak at *m*/*z* 294.1828 [M]^+^ (calcd. for C_17_H_26_O_4_, 294.1826). The NMR spectroscopic data of Compound **8** were very similar to those of Compound **7**, except that the C-2 hydroxyl group in **7** was replaced by an acetoxy group in **8** (Supplementary Figures S30 and S31). Fortunately, we obtained a single crystal of **8** from the MeOH solution. In the crystal, the adjacent molecules are interlinked by a pair of strong O1-H...O4 (hydroxyl) and O4-H...O3 (carbonyl) hydrogen bonds to form a zigzag chain running parallel to the b-axis. Neighboring chains of molecules are packed closely together with the hydrophobic methyl groups pointing towards each other. The absolute configuration of **8** was determined as 1*S*, 2*R*, 4a*R*, 5*R*, 8*R*, 8a*R* with the Flack parameter value −0.16(17) by single-crystal X-ray diffraction analysis (Figure 4 and Supplementary Crystallographic Information Framework (CIF)) using Cu K*α* radiation. Compound **8**, named (1*S*,2*R*,4a*R*,5*R*,8*R*,8a*R*)-1,8a-dihydroxy-2-acetoxy- 3,8-dimethyl-5-(prop-1-en-2-yl)-1,2,4a,5,6,7,8,8a-octahydronaphthalene, was previously obtained as the degradation product of the natural product CJ-12662, which was obtained from the fermentation broth of *Aspergillus fischeri* var. *thermomutatus* ATCC 18,618 [19] and later also isolated from fungi *Neosartorya pseudofischeri* and *Eurotium chevalieri* [20,21]. However, the single-crystal X-ray diffraction data were never reported.

Compound **9** was obtained as a white powder. Its molecular formula was deduced to be C15H14O4 by analysis of the ^13^C-NMR data and HR(-)ESIMS ion at *m*/*z* 257.0817 [M − H]^−^ (calcd. for C_15_H_13_O_4_, 257.0819), which required nine degrees of unsaturation (Supplementary Figure S32). The IR spectrum revealed the presence of a carboxyl group (3283 and 1671 cm^−1^) and an aldehyde group (2812 cm^−1^). Two methyl groups at *δ*_H_ 1.45 (d, *J* = 7.0 Hz, 6H) showed ^1^H–^1^H COSY correlations with the methine group at *δ*_H_ 3.90 (seven peaks, *J* = 7.0 Hz) and formed an isopropyl fragment (Table 2, Figure 2 and Supplementary Figures S33–S36). According to the HMBC correlations of H-1/C-2, H-1/C-8a, H-3/C-2 and H-4/C-4a, the protons at *δ*_H_ 8.92 (d, *J* = 1.5 Hz, H-1), 8.22 (dd, *J* = 9.0, 1.5 Hz, H-3) and 8.77 (d, *J* = 9.0 Hz, H-4) were located at the 1,3,4-position on a phenyl ring. HMBC correlations of H-1/C-2, H-1/C-8a, H-3/C-2, H-4/C-4a, H-4/C-5, H-7/C-5, H-7/C-6, H-11/C-7 and H-11/C-8 indicated the existence of a naphthyl ring, accounting for seven degrees of unsaturation. The remaining carboxyl group (*δ*_C_ 167.6, *δ*_H_ 10.94), the aldehyde (*δ*_C_ 195.3, *δ*_H_ 11.42), phenolic hydroxyl group (*δ*_H_ 13.42, and the isopropyl fragment were located on C-2, C-5, C-6 and C-8, respectively, based on the HMBC cross peaks of H-1/C-9, H-3/C-9, H-10/C-6, 6-OH/C-6, 6-OH/C-7 and H-11/C-8. The aldehyde and hydroxyl groups formed an intramolecular hydrogen bond, which accounted for the downfield resonance of the phenolic hydroxyl group. Therefore, the structure of **9** was elucidated as 5-formyl-6-hydroxy-8-isopropyl-2-naphthoic acid.

Compound **10** was isolated as a white powder. The ^13^C-NMR data and HR(-)ESIMS peak at *m*/*z* 245.0815 [M − H]^+^ (calcd. for C_14_H_13_O_4_, 245.0819), established the molecular formula as C_14_H_14_O_4_ (Supplementary Figure S37). The ^13^C-NMR and DEPT spectra revealed fourteen carbon resonances, including one methyl group, one methylene group, seven methine groups and five quaternary carbons. The ^1^H-NMR spectrum exhibited two meta-aromatic protons at *δ*_H_ 6.24 (d, *J* = 2.0 Hz) and 6.18 (d, *J* = 2.0 Hz), indicating the presence of a tetrasubstituted aromatic ring. Two phenolic hydroxyl groups at *δ*_H_ 11.06 (brs) were connected to C-6 (*δ*_C_ 163.4) and C-8 (*δ*_C_ 164.8). Additionally, two pairs of trans-oriented olefinic protons at *δ*_H_ 5.71 (dd, *J* = 15.2, 6.8 Hz), 6.35 (dd, *J* = 15.2, 10.0 Hz), 6.10 (ddd, *J* = 15.2, 10.0, 1.2 Hz), 5.81 (dq, *J* = 15.2, 6.8 Hz), one oxymethine at *δ*_H_ 5.13 (ddd, *J* = 10.0, 6.8, 4.0 Hz), one methylene at *δ*_H_ 3.00 (dd, *J* = 16.4, 4.0 Hz), 2.93 (dd, *J* = 16.4, 10.0 Hz) and one methyl group at *δ*_H_ 1.74 (dd, *J* = 6.8, 1.2 Hz) were connected in sequence and formed a pentadienyl group based on their ^1^H–^1^H COSY correlations. The remnant quaternary carbon (*δ*_C_ 169.1) connected with C-8a (*δ*_C_ 100.1) and the oxymethine and formed a lactone. The HMBC correlations of H-4/C-4a, H-4/C-8a, H-5/C-6, H-7/C-8 and H-7/C-8a revealed a 3,6,8-trisubstituted isocoumarin ring system (Table 2, Figure 2 and Supplementary Figures S38–S43). The absolute configuration at C-3 remains undetermined. Therefore, Compound **10** was elucidated as 6,8-dihydroxy-3-((1*E*,3*E*)-penta-1,3- dien-1-yl)isochroman-1-one.

The other known compounds, isochaetominine C (**11**) [22], trichodermamide A (**12**) [23], indolyl-3-acetic acid methyl ester (**13**) [24], 1-acetyl-β-carboline (**14**) [25], 1,2,3,4-tetrahydro- 6-hydroxy-2-methyl-l,3,4-trioxopyrazino[l,2-a]-indole (**15**) [26] and fumiquinazoline F (**16**) [27], were identified by comparing their spectroscopic data with the literature values (Supplementary Figures S44–S55).

### 2.2. Proposed Biosynthetic Pathways

**Figure 5:**
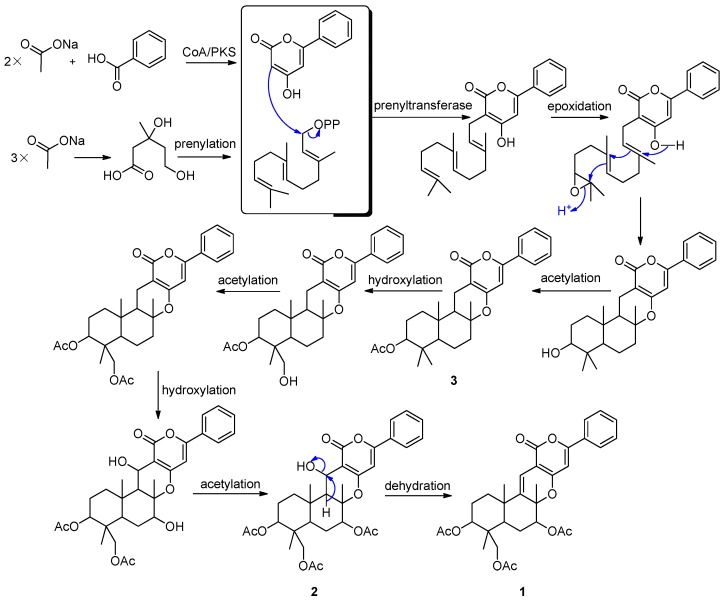
Proposed biosynthetic pathways of **1**–**3**.

Tomoda *et al.* described the biosynthetic origin of pyripyropene A (**5**), which was established by feeding experiments using various [^13^C] and [^14^C] precursors. Pyripyropene A (**5**) is derived from three mevalonates, five acetates and one nicotinic acid. The pyridino-α-pyrone moiety is produced via condensation of a primer nicotinic acid with two acetates in a “head-to-tail” fashion; an all-trans farnesyl pyrophosphate is produced via the mevalonate pathway; the two parts are linked and cyclized to form the core skeleton, and then three acetyl residues from the acetates are introduced into the skeleton to yield **5** [28]. Based on the above finding, we proposed that the phenyl-α-pyrones (**1**–**3**) shared a similar biosynthetic pathway (Figure 5). Benzoic acid, instead of nicotinic acid, is one of the precursors. It is very interesting that Compound **2** was observed slowly changing into Compound **1** when stored in DMSO-*d*_6_. The change was recorded by ^1^H-NMR spectra. After 18 days, Compound **2** was changed into Compound **1** thoroughly. However, the pure Compounds **1** and **2** are stable at the temperature range from −20 °C–90 °C.

### 2.3. Cytotoxicity

Compounds **1**–**11** and **15** showed significant cytotoxicity against the Sf9 cells (Table 3). After 48 h of treatment at the concentration of 50 mg/L, the cell growth inhibition rates of compounds **2**, **3**, **7**–**9**, **11** and **15** were greater than 90%. Therefore, further in-depth studies to identify the insecticidal activities *in vivo* and the mechanism are warranted for their development as biorational pesticides.

**Table 3:** The death rate (%) of Compounds **1**–**11** and **15** against insect cell line Sf9 (*n* = 3, *p* ≤ 0.05).

Compounds	6 h	12 h	24 h	48 h
1	65.79	68.26	76.16	85.24
2	60.75	68.86	81.21	95.01
3	72.55	72.15	73.08	93.73
4	47.53	57.41	73.21	85.37
5	41.05	54.60	58.04	71.77
6	25.58	29.46	35.30	56.03
7	79.58	81.89	90.46	98.68
8	64.52	69.33	77.98	91.87
9	57.96	65.32	69.92	90.97
10	36.47	38.25	52.36	61.67
11	63.70	69.16	71.65	94.53
15	56.97	61.43	80.33	92.52
Rotenone	69.21	79.43	90.93	98.54

## 3. Experimental Section

### 3.1. General Experimental Procedures

Preparative HPLC was performed using a Shimadzu LC-20AT HPLC pump (Shimadzu Corporation, Nakagyo-ku, Kyoto, Japan) equipped with an SPD-20A dual λ absorbance detector (Shimadzu Corporation, Nakagyo-ku, Kyoto, Japan) and a Shim-pack PRC-ODS HPLC column (250 mm × 20 mm, Shimadzu Corporation, Nakagyo-ku, Kyoto, Japan). Optical rotations were measured using a Schmidt and Haensch Polartronic HNQW5 optical rotation spectrometer (SCHMIDT + HAENSCH GmbH & Co., Berlin, Germany). UV spectra were recorded on a Shimadzu UV-VIS-NIR spectrophotometer (Shimadzu Corporation, Nakagyo-ku, Kyoto, Japan). IR spectra were recorded on a PerkinElmer Frontier FT-IR spectrophotometer (PerkinElmer Inc., Waltham, MA, USA). 1D and 2D NMR spectra were recorded on Bruker Avance II 400 spectrometers (Bruker BioSpin AG, Industriestrasse 26, Fällanden, Switzerland) and a Varian INOVA500NB spectrometer (Varian Medical Systems In., Salt Lake City, UT, USA). The chemical shifts are relative to the residual solvent signals (CDCl_3_: *δ*_H_ 7.26 and *δ*_C_ 77.0; acetone-*d*_6_: *δ*_H_ 2.05 and *δ*_C_ 29.92; DMSO-*d*_6_: *δ*_H_ 2.50 and *δ*_C_ 39.51). The low- and high-resolution EI mass spectra were obtained on Thermo DSQ and Thermo MAT95XP mass spectrometers (Thermo Fisher Scientific, Waltham, MA, USA), respectively. The low- and high-resolution ESI-MS analyses were performed with a Thermo LCQ DECA XP liquid chromatography-mass spectrometry (Thermo Fisher Scientific, Waltham, MA, USA) and a Thermo Fisher LTQ Orbitrap Elite High Resolution liquid chromatography-mass spectrometry (Thermo Fisher Scientific, Waltham, MA, USA). X-ray diffraction data were acquired on a Bruker SMART APEX CCD X-ray single-crystal diffractometer (Bruker AXS GmbH, Karlsruhe, Germany).

### 3.2. Fungal Material

The marine fungus *N. pseudofischeri* was isolated from the inner tissue of the starfish *A. planci* collected from the Hainan Sanya National Coral Reef Reserve, China. The fungus was identified on the basis of the DNA sequences of the ITS1-5.8S-ITS2 (Internal Transcribed Spacer, ITS) regions of their rRNA gene. The ITS gene sequence showed 100% homology with that of the fungus *N. pseudofischeri* in GenBank (Accession Number KF999816).

### 3.3. Fermentation, Extraction and Isolation

The 20 L scale-up fermentation of *N. pseudofischeri* was carried out in 1000 mL Erlenmeyer flasks containing 500 mL liquid culture media, which was composed of glucose 10 g, peptone 5 g, yeast extract 2 g, CaCO_3_ 1 g and sea water 1 L. After 30 days of growth, the mycelia of *N. pseudofischeri* were extracted with methanol, and the organic solvent was evaporated to dryness under vacuum to afford the crude extract (5.36 g). Then, the extract was separated by flash silica gel column chromatography using petroleum ether-ethyl acetate in a gradient elution (100:0–0:100, *v*/*v*), followed by ethyl acetate-methanol in a gradient elution (100:0–0:100, *v*/*v*) to give 10 fractions (F1–F10). Subfractions F4.1–F4.6 were obtained from F4 (610 mg) by reverse phase silica gel using methanol-water (30:70–100:0, *v*/*v*) gradient elution. Then, F4.3 (44 mg) was further separated by RP-HPLC using methanol-water (65:35, *v*/*v*) as the eluent to obtain Compounds **1** (6.2 mg), **4** (4.1 mg) and **5** (3.4 mg). F4.4 (32 mg) was further separated by RP-HPLC eluted with methanol-water (68:32, *v/v*) to obtain Compounds **2** (10.1 mg), **3** (3.5 mg), **6** (1.7 mg) and **12** (2.4 mg). F5 (157 mg) was further separated by RP-HPLC eluted with methanol-water (70:30, *v*/*v*) to obtain Compound **8** (32.6 mg). F6 (113 mg) was further separated by RP-HPLC eluted with methanol-water (60:40, *v*/*v*) to obtain **7** (12.1 mg) and **9** (2.8 mg). F7 (201 mg) was separated by RP-HPLC eluted with MeCN-water (60:40, *v*/*v*) to get Compounds **10** (4.5 mg), **11** (3.1 mg) and **16** (2.3 mg). F8 (97 mg) was further separated by RP-HPLC eluted with MeCN-water (50:50, *v*/*v*) to obtain Compounds **13** (2.0 mg), **14** (2.9 mg) and **15** (23.2 mg).

5-Olefin phenylpyropene A (**1**): yellowish oil; ${\left[\alpha \right]}_{D}^{20}$: + 129° (*c* = 0.1, CHCl_3_); UV (MeOH) λ_max_ (ɛ) 234 (13,810), 322 (10,108) nm; IR *ν*_max_ 2960, 2925, 1735, 1602, 1566, 1451, 1378, 1241, 1043, 758 cm^−1^; for ^1^H and ^13^C-NMR data, see Table 1; LR(+)ESIMS *m*/*z* 565.3 [M + H]^+^. HR(+)ESIMS *m*/*z* 565.2448 [M + H]^+^ (calcd. for C_32_H_3__7_O_9_, 565.2432).

13-Dehydroxylpyripyropene A (**4**): white powder; ${\left[\alpha \right]}_{D}^{20}$: +20° (*c* = 0.1, CHCl_3_); white powder; UV (MeOH) λ_max_ (ɛ) 216 (18,120), 246 (12,977), 278 (4038) nm; IR *ν*_max_ 2960, 2921, 2851, 1713, 1464, 1377, 1260, 1035, 810, 721 cm^−1^. For ^1^H and ^13^C-NMR data, see Table 1; LR(+)ESIMS *m*/*z* 568.2 [M + H]^+^. HR(+)ESIMS *m*/*z* 568.2549 [M + H]^+^ (calcd. for C_31_H_38_NO_9_, 568.2541).

7-deacetylpyripyropene A (**6**): white powder; for ^1^H and ^13^C-NMR data, see Table 1.

Deacetylsesquiterpene (**7**): yellowish oil; ${\left[\alpha \right]}_{D}^{20}$: +38° (*c* = 0.058, MeOH); UV (MeOH) λ_max_ (ɛ) 212 (136) nm; IR *ν*_max_ 3443, 2959, 2926, 2856 1721, 1675, 1609, 1455, 1376, 1260, 1095, 1029, 992, 887, 756 cm^−1^. For ^1^H and ^13^C-NMR data, see Table 2; HR(+)ESIMS: *m*/*z* 275.1603 [M + Na]^+^ (calcd. for C_15_H_24_O_3_Na, 275.1618).

(1S,2R,4aR,5R,8R,8aR)-1,8a-dihydroxy-2-acetoxy-3,8-dimethyl-5-(prop-1-en-2-yl)-1,2,4a,5,6,7,8, 8a-octahydronaphthalene (**8**): white crystal. ${\left[\alpha \right]}_{D}^{20}$: −68° (*c* = 0.079, MeOH); IR (MeOH) *ν*_max_ 3677, 3436, 3317, 2928, 2862, 1731, 1642, 1444, 1405, 1369, 1238, 1030, 967, 945, 927, 885, 720, 609 cm^−1^. For ^1^H and ^13^C-NMR spectra, see Supplementary Figures S30 and S31; HREIMS: *m*/*z* 294.1828 [M]^+^ (calcd. for C_17_H_26_O_4_, 294.1826). The crystal of **8** was obtained from MeOH solution. C_17_H_26_O_4_, *M* = 294.38, colorless block, Orthorhombic system, P 2_1_ 2_1_ 2_1_ (19), a = 8.3364 (1), b = 9.3709 (2), c = 21.3150 (5) Å. V = 1665.12 (6) Å^3^, *Z* = 4. *D*_calcd_ = 1.174 g/cm^3^, crystal size 0.41 mm × 0.45 mm × 0.43 mm, F(000) = 640, T = 173(2) K.

5-formyl-6-hydroxy-8-isopropyl-2-naphthoic acid (**9**): white powder; IR *ν*_max_ 3283, 3020, 2953, 2928, 2867, 2812, 2720, 1671, 1616, 1601, 1512, 1458, 1422, 1393, 1289, 1026, 1208, 1148, 1109, 1080, 1008, 985, 921, 796 cm^−1^; for ^1^H and ^13^C-NMR data, see Table 2; HR(-)ESIMS *m*/*z* 257.0817 [M − H]^−^ (calcd. for C_15_H_13_O_4_, 257.0819).

6,8-dihydroxy-3-((1E,3E)-penta-1,3-dien-1-yl)isochroman-1-one (**10**): white powder; ${\left[\alpha \right]}_{D}^{20}$: +40° (*c* = 0.1, MeOH); UV (MeOH) λ_max_ (ɛ) 232 (6312), 268 (3427), 302 (2060) nm; IR *ν*_max_ 3528, 3400, 2953, 2926, 2855, 1656, 1622, 1460, 1374, 1247, 1110, 1003, 772 cm^−1^. For ^1^H and ^13^C-NMR data, see Table 2; HR(-)ESIMS *m*/*z* 245.0815 [M − H]^−^ (calcd. for C_14_H_13_O_4_, 245.0819).

### 3.4. Bioassay

To evaluate the biological activities of these compounds, cytotoxic assays were carried out with insect cultured cell line Sf9 from *S. frugiperda*. Sf9 cells were maintained at 27 °C in TC-199-MK medium supplemented with 10% FCS (*v*/*v*), 1% L-glutamine 200 mmol/L and penicillin-streptomycin-neomycin solutions (*v*/*v*). The cell growth inhibition was measured by the MTT method. Cells were seeded in 96-well microtitration plates at the exponential growth phase. Different concentrations of compounds diluted with medium were added at their log-phase growth stage. Additionally, 0.2 μL of solvent (DMSO) only was added as the control (CK). The final concentration of solvent in the cultures assayed was 1%. Compounds were solubilized with DMSO at concentrations of 10 and 50 mg/L. The rotenone was used as the positive control. After 6 h, 12 h, 24 h and 48 h of treatment, 5 mg/mL MTT were dissolved in PBS, and 20 μL of this stock solution were added to the culture cells. After an additional 3 h of incubation, the medium was discarded, and the 96-well plates were dried in the air. Then, 100 μL of DMSO were added to dissolve the formazan crystals, and the absorbance was measured at 570 nm by a microplate reader (Spectramex, 190 Molecular Devices Inc., Sunnyvale, CA, USA).

The cytotoxic effect was expressed as the relative percentage of inhibition calculated as follows: cell growth inhibition rate (%) = [(A control − A treatment)/A control] × 100 [29].

## 4. Conclusions

In summary, sixteen compounds, including five new compounds (**1**, **4**, **7**, **9** and **10**), were obtained from the mycelium of marine fungus *N. pseudofischeri*. Their structure types include phenylpyripyropenes, pyripyropenes, sesquiterpenoids, isocoumarin and alkaloids. Our findings provide further evidence that the marine fungus *N. pseudofischeri* has the tremendous potential of biosynthesis. The potent cytotoxicity of these compounds against the Sf9 cells revealed the prospect to develop biorational pesticides.

## Supplementary Files

Supplementary File 1
