# Supplementary material for: Five New Cytotoxic Metabolites from the Marine Fungus Neosartorya pseudofischeri
Source: Mar Drugs. 2016 Jan 13;14(1):18. doi: 10.3390/md14010018 (PMC4728515; doi:10.3390/md14010018)
Supplement: Supplementary File 1 [file marinedrugs-14-00018-s001.pdf]

Supplementary Materials: Five New Cytotoxic Metabolites from the Marine Fungus *Neosartorya pseudofischeri*

Wen-Jian Lan, Sheng-Jiao Fu, Meng-Yang Xu, Wan-Ling Liang, Chi-Keung Lam, Guo-Hua Zhong, Jun Xu, De-Po Yang and Hou-Jin Li

List of Supporting Information	Page
**Figure S1.** HR(+)ESIMS spectrum of 5-olefin phenylpyropene A (**1**)	S2
**Figure S2.** ^{1}H NMR spectrum of 5-olefin phenylpyropene A (**1**) in $CDCl_3$, 400 MHz	S3
**Figure S3.** ^{13}C NMR spectrum of 5-olefin phenylpyropene A (**1**) in $CDCl_3$, 100 MHz	S3
**Figure S4**. HSQC spectrum of 5-olefin phenylpyropene A (**1**)	S4
**Figure S5.** ^{1}H-^{1}H COSY spectrum of 5-olefin phenylpyropene A (**1**)	S4
**Figure S6.** HMBC spectrum of 5-olefin phenylpyropene A (**1**)	S5
**Figure S7.** NOESY spectrum of 5-olefin phenylpyropene A (**1**)	S5
**Figure S8.** ^{1}H NMR spectrum of phenylpyropene A (**2**) in $CDCl_3$, 400 MHz	S6
**Figure S9.** ^{13}C NMR spectrum of phenylpyropene A (**2**) in $CDCl_3$, 100 MHz	S6
**Figure S10.** ^{1}H NMR spectrum of phenylpyropene C (**3**) in $CDCl_3$, 400 MHz	S7
**Figure S11.** ^{13}C NMR spectrum of phenylpyropene C (**3**) in $CDCl_3$, 100 MHz	S7
**Figure S12.** HR(+)ESIMS spectrum of 13-dehydroxylpyripyropene A (**4**)	S8
**Figure S13.** ^{1}H NMR spectrum of 13-dehydroxylpyripyropene A (**4**) in $CDCl_3$, 400 MHz	S8
**Figure S14.** ^{13}C NMR spectrum of 13-dehydroxylpyripyropene A (**4**) in $CDCl_3$, 100 MHz	S9
**Figure S15.** HSQC spectrum of 13-dehydroxylpyripyropene A (**4**)	S9
**Figure S16.** ^{1}H-^{1}H COSY spectrum of13-dehydroxylpyripyropene A (**4**)	S10
**Figure S17.** HMBC spectrum of 13-dehydroxylpyripyropene A (**4**)	S10
**Figure S18.** NOESY spectrum of 13-dehydroxylpyripyropene A (**4**)	S11
**Figure S19.** ^{1}H NMR spectrum of pyripyropene A (**5**) in $CDCl_3$, 400 MHz	S11
**Figure S20.** ^{13}C NMR spectrum of pyripyropene A (**5**) in $CDCl_3$, 100 MHz	S12
**Figure S21.** ^{1}H NMR spectrum of 7-deacetylpyripyropene A (**6**) in acetone-d_6, 400 MHz	S12
**Figure S22.** ^{13}C NMR spectrum of 7-deacetylpyripyropene A (**6**) in acetone-d_6, 100 MHz	S13
**Figure S23.** HR(+)ESIMS spectrum of deacetylsequiterpene (**7**)	S13
**Figure S24.** ^{1}H NMR spectrum of deacetylsequiterpene (**7**) in $CDCl_3$, 400 MHz	S14
**Figure S25.** ^{13}C NMR spectrum of deacetylsequiterpene (**7**) in $CDCl_3$, 100 MHz	S14
**Figure S26.** HSQC spectrum of deacetylsequiterpene (**7**)	S15
**Figure S27.** ^{1}H-^{1}H COSY spectrum of deacetylsequiterpene (**7**)	S15
**Figure S28.** HMBC spectrum of deacetylsequiterpene (**7**)	S16
**Figure S29.** NOESY spectrum of deacetylsequiterpene (**7**)	S16
**Figure S30.** ^{1}H NMR spectrum of sesquiterpene (**8**) in $CDCl_3$, 400 MHz	S17
**Figure S31.** ^{13}C NMR spectrum of sesquiterpene (**8**) in $CDCl_3$, 100 MHz	S17
**Figure S32**.HR(-)ESIMS spectrum of 5-formly-6-hydroxy-8-isopro-pyl-2-naphthoic acid (**9**)	S18
**Figure S33.** ^{1}H NMR spectrum of 5-formly-6-hydroxy-8-isopro-pyl-2-naphthoic acid (**9**) in Acetone-d_6, 500 MHz	S18
**Figure S34.** ^{13}C NMR spectrum of 5-formly-6-hydroxy-8-isopro-pyl-2-naphthoic acid (**9**) in Acetone-d_6, 125 MHz	S19
**Figure S35.** HSQC spectrum of 5-formly-6-hydroxy-8-isopro-pyl-2-naphthoic acid (**9**)	S19
**Figure S36.** HMBC spectrum of 5-formly-6-hydroxy-8-isopro-pyl-2-naphthoic acid (**9**)	S20
**Figure S37.** HRESIMS spectrum of 6,8-dihydroxy-3-((1*E*,3*E*)-penta-1,3-dien-1-yl)isochroman-1-one (**10**)	S20
**Figure S38.** ^{1}H NMR spectrum of6,8-dihydroxy-3-((1*E*,3*E*)-penta-1,3-dien-1-yl)isochroman-1-one (**10**) in DMSO-d_6, 400 MHz	S21
**Figure S39.** ^{13}C NMR spectrum of6,8-dihydroxy-3-((1*E*,3*E*)-penta-1,3-dien-1-yl)isochroman-1-one (**10**) in DMSO-d_6, 100 MHz	S21
**Figure S40.** HSQC spectrum of 6,8-dihydroxy-3-((1*E*,3*E*)-penta-1,3-dien-1-yl)isochroman-1-one (**10**)	S22

**Figure S41.** ^{1}H-^{1}H COSY spectrum of 6,8-dihydroxy-3-((1*E*,3*E*)-penta-1,3-dien-1-yl)isochroman-1-one (**10**)	S22
**Figure S42.** HMBC spectrum of 6,8-dihydroxy-3-((1*E*,3*E*)-penta-1,3-dien-1-yl)isochroman-1-one (**10**)	S23
**Figure S43.** NOESY spectrum of 6,8-dihydroxy-3-((1*E*,3*E*)-penta-1,3-dien-1-yl)isochroman-1-one (**10**)	S23
**Figure S44.** ^{1}H NMR spectrum of isochaetominine C (**11**) in $CDCl_3$, 400 MHz	S24
**Figure S45.** ^{13}C NMR spectrum of isochaetominine C (**11**) in $CDCl_3$, 100 MHz	S24
**Figure S46.** ^{1}H NMR spectrum of trichodermamide A (**12**) in DMSO-d_6, 400 MHz	S25
**Figure S47.** ^{13}C NMR spectrum of trichodermamide A (**12**) in DMSO-d_6, 100 MHz	S25
**Figure S48.**^{1}H NMR spectrum of indolyl-3-acetic acid methyl ester (**13**) in DMSO-d_6, 400 MHz	S26
**Figure S49.** ^{13}C NMR spectrum of indolyl-3-acetic acid methyl ester (**13**) in DMSO-d_6, 100 MHz	S26
**Figure S50.** ^{1}H NMR spectrum of 1-(9*H*-β-carbolin-1-yl)-ethanone (**14**) in $CDCl_3$, 400 MHz	S27
**Figure S51.** ^{13}C NMR spectrum of 1-(9*H*-β-carbolin-1-yl)-ethanone (**14**) in $CDCl_3$, 100 MHz	S27
**Figure S52.** ^{1}H NMR spectrum of 1,2,3,4-tetrahydro-6-hydroxyl-2-methyl-l,3,4-trioxopyrazino[l,2-a]-indole (**15**) in DMSO-d_6, 400 MHz	S28
**Figure S53.** ^{13}C NMR spectrum of 1,2,3,4-tetrahydro-6-hydroxyl-2-methyl-l,3,4-trioxopyrazino[l,2-a]-indole (**15**) in DMSO-d_6, 100 MHz	S28
**Figure S54.** ^{1}H NMR spectrum of fumiquinazoline F (**16**) in $CDCl_3$, 400 MHz	S29
**Figure S55.** ^{13}C NMR spectrum of fumiquinazoline F (**16**) in $CDCl_3$, 100 MHz	S29
**CIF of Sesquiterpene** (8)	S30

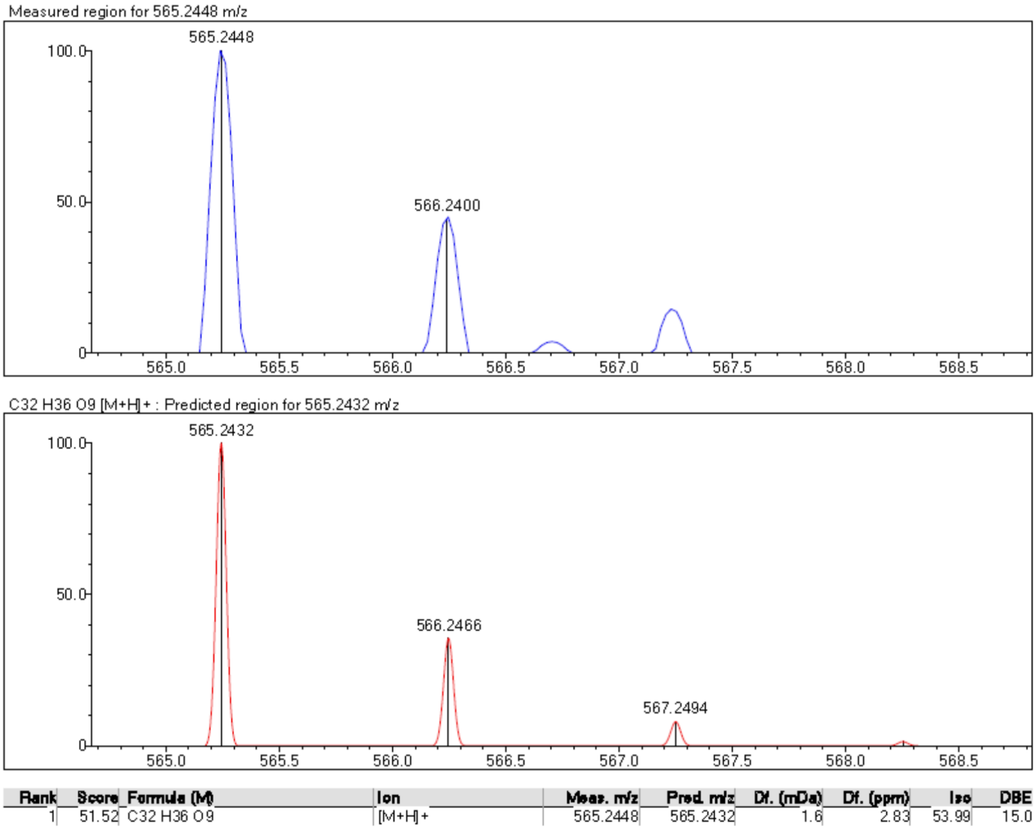

Figure S1. HR(+)ESIMS spectrum of 5-olefin phenylpyropene A (**1**).

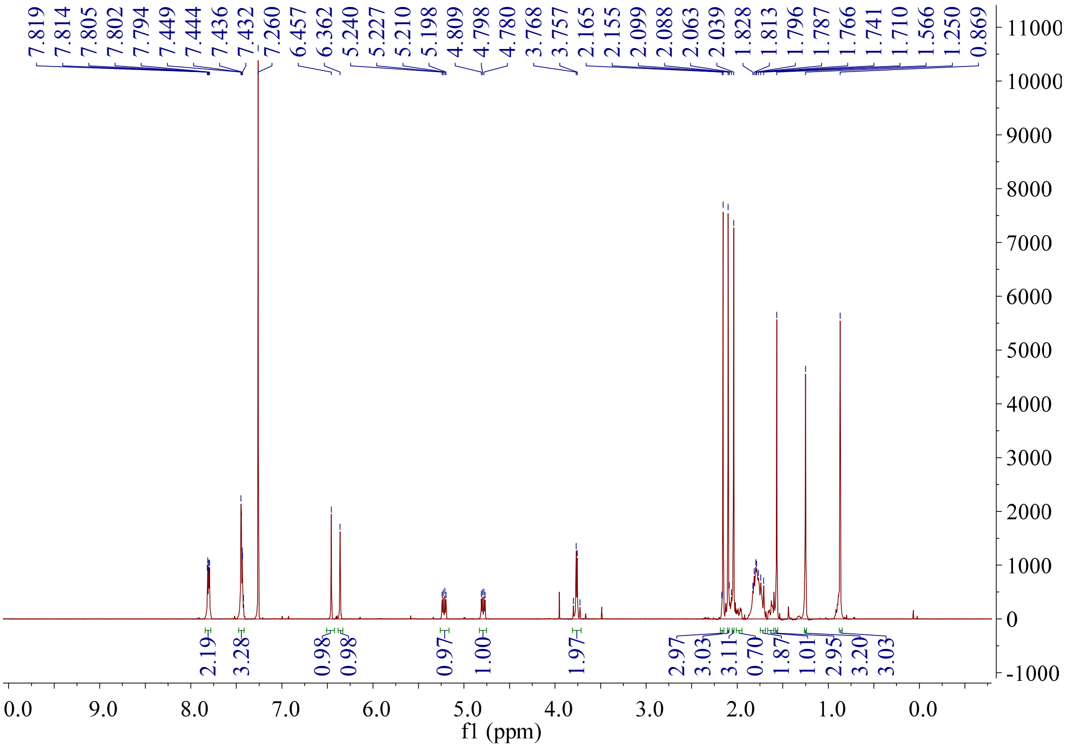

Figure S2. ^{1}H NMR spectrum of 5-olefin phenylpyropene A (**1**) in $CDCl_3$, 400 MHz.

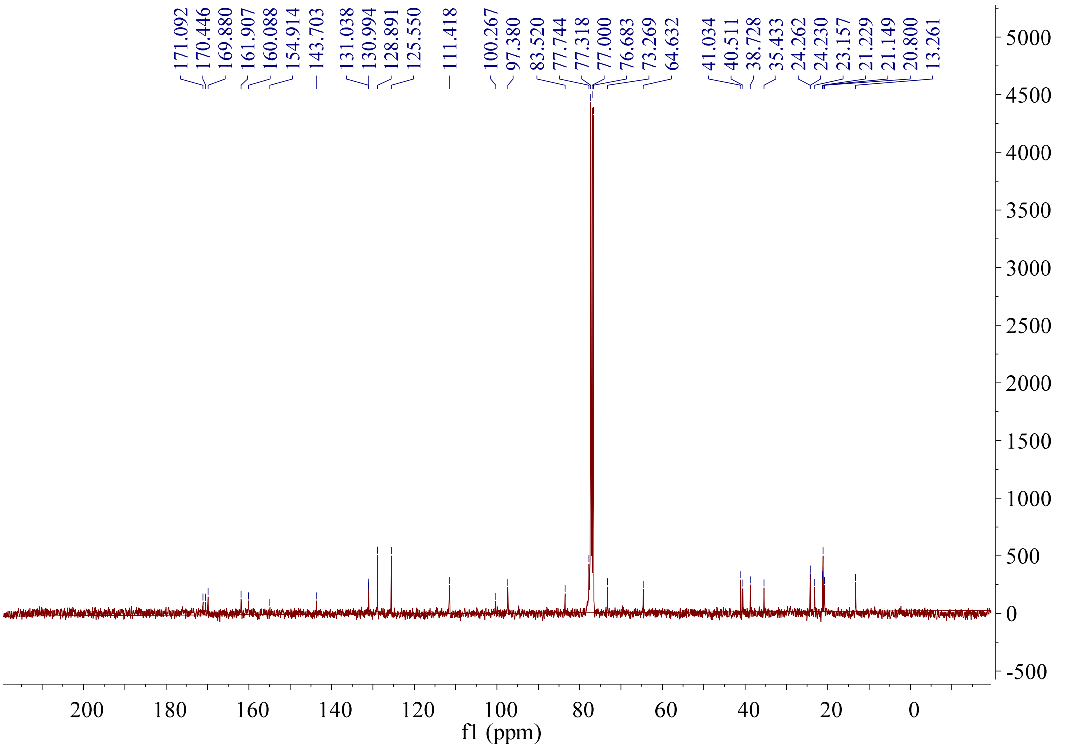

Figure S3. ^{13}C NMR spectrum of 5-olefin phenylpyropene A (**1**) in $CDCl_3$, 100 MHz.

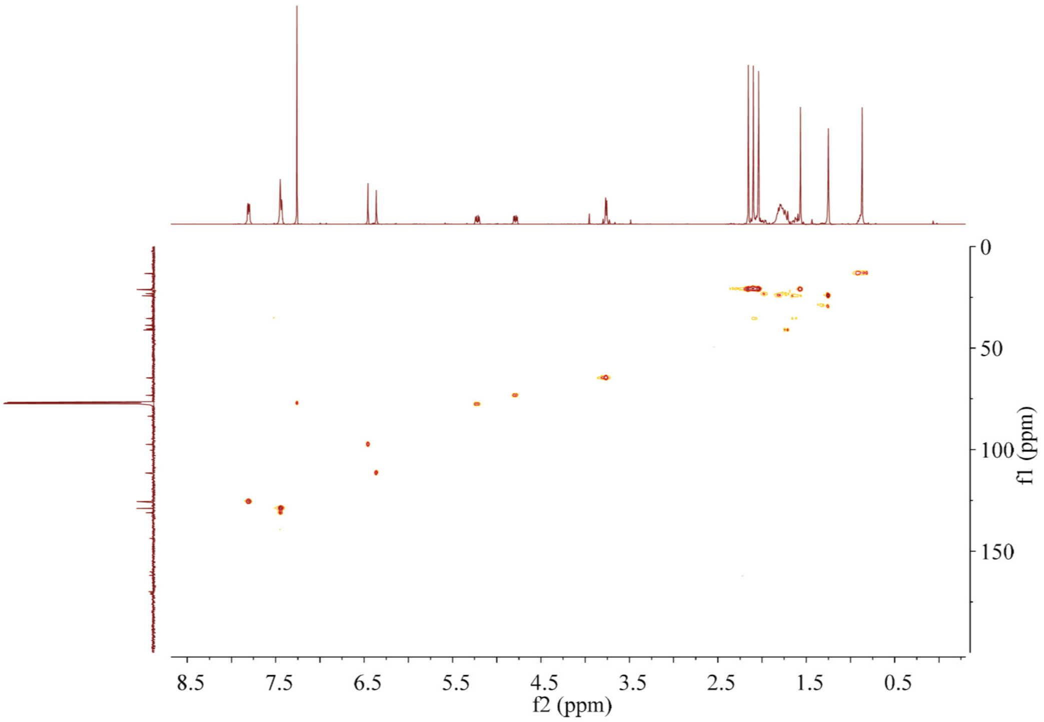

Figure S4. HSQC spectrum of 5-olefin phenylpyropene A (**1**).

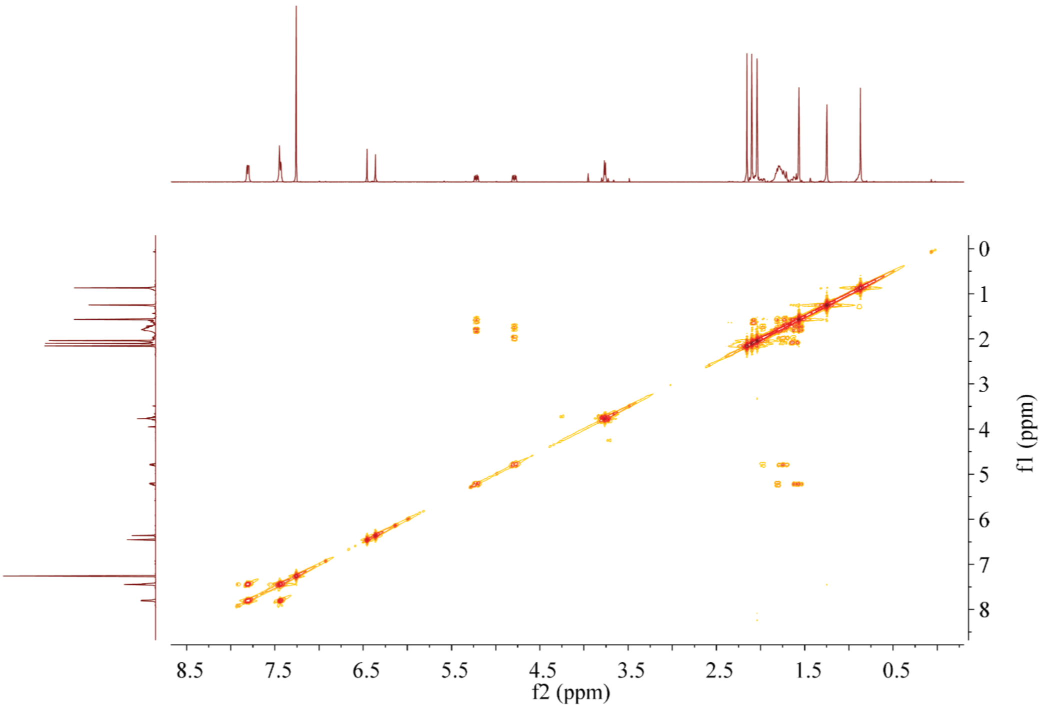

Figure S5. ^{1}H-^{1}H COSY spectrum of 5-olefin phenylpyropene A (**1**).

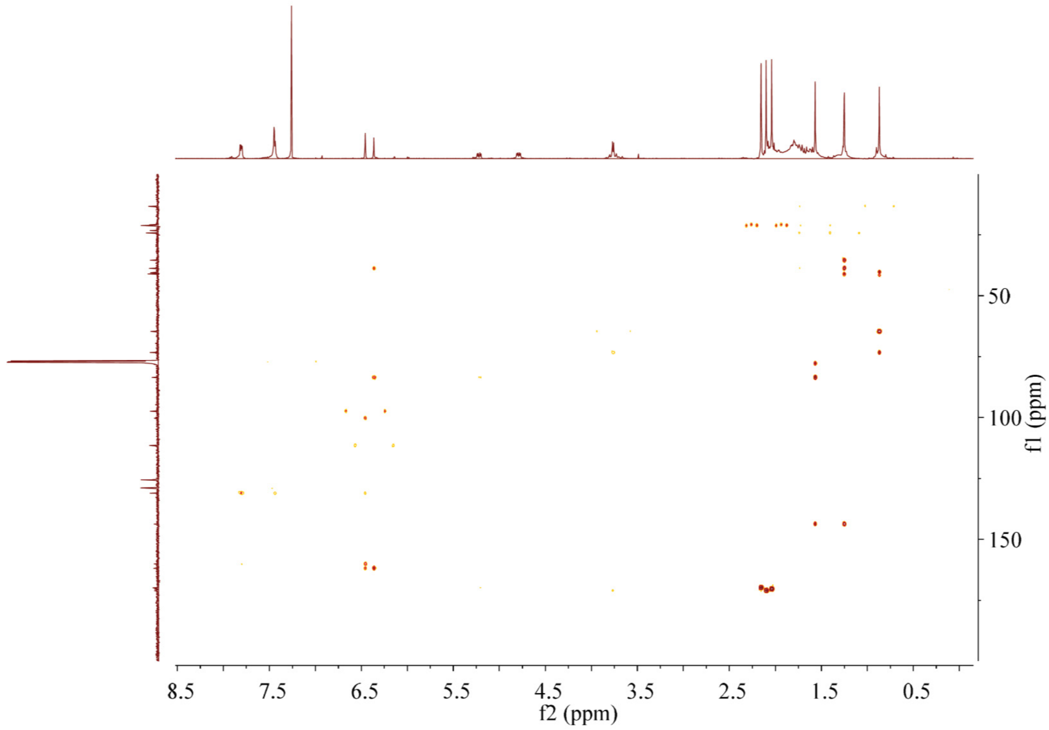

Figure S6. HMBC spectrum of 5-olefin phenylpyropene A (**1**).

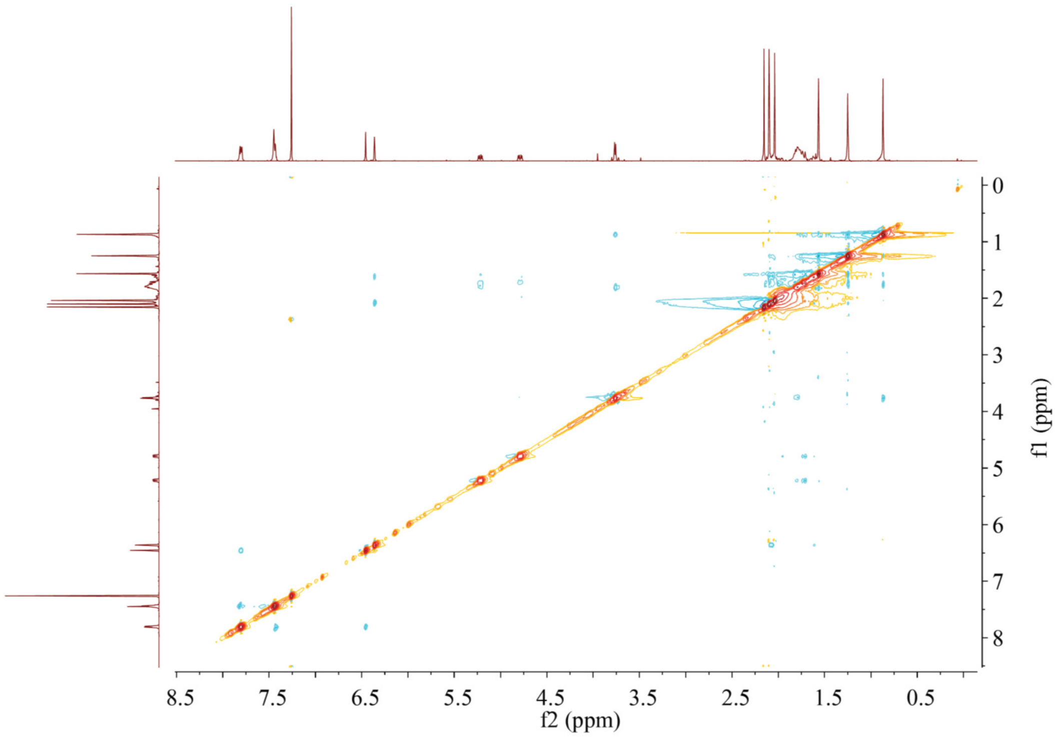

Figure S7. NOESY spectrum of 5-olefin phenylpyropene A (**1**).

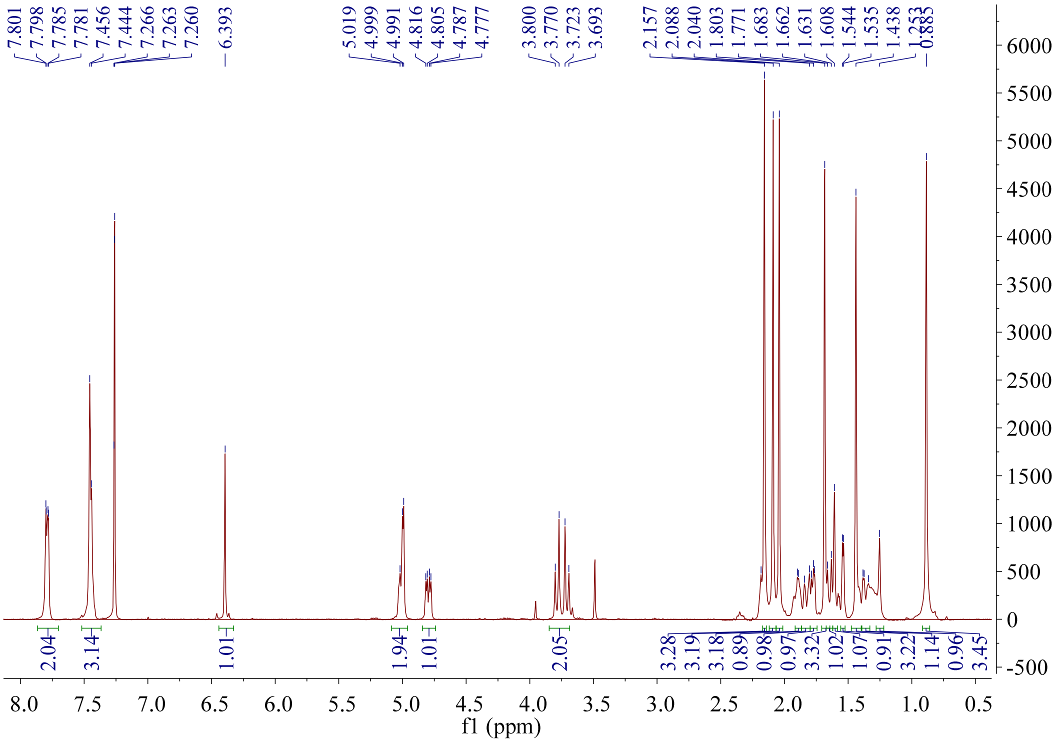

Figure S8. ^{1}H NMR spectrum of phenylpyropene A (**2**) in $CDCl_3$, 400 MHz.

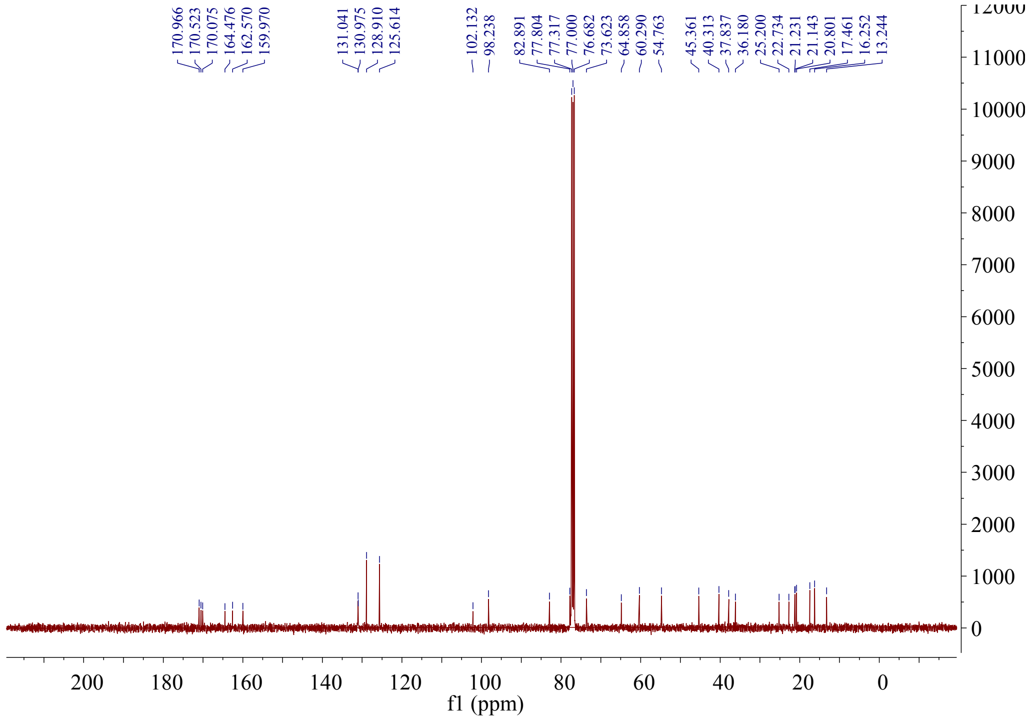

Figure S9. ^{13}C NMR spectrum of phenylpyropene A (**2**) in $CDCl_3$, 100 MHz.

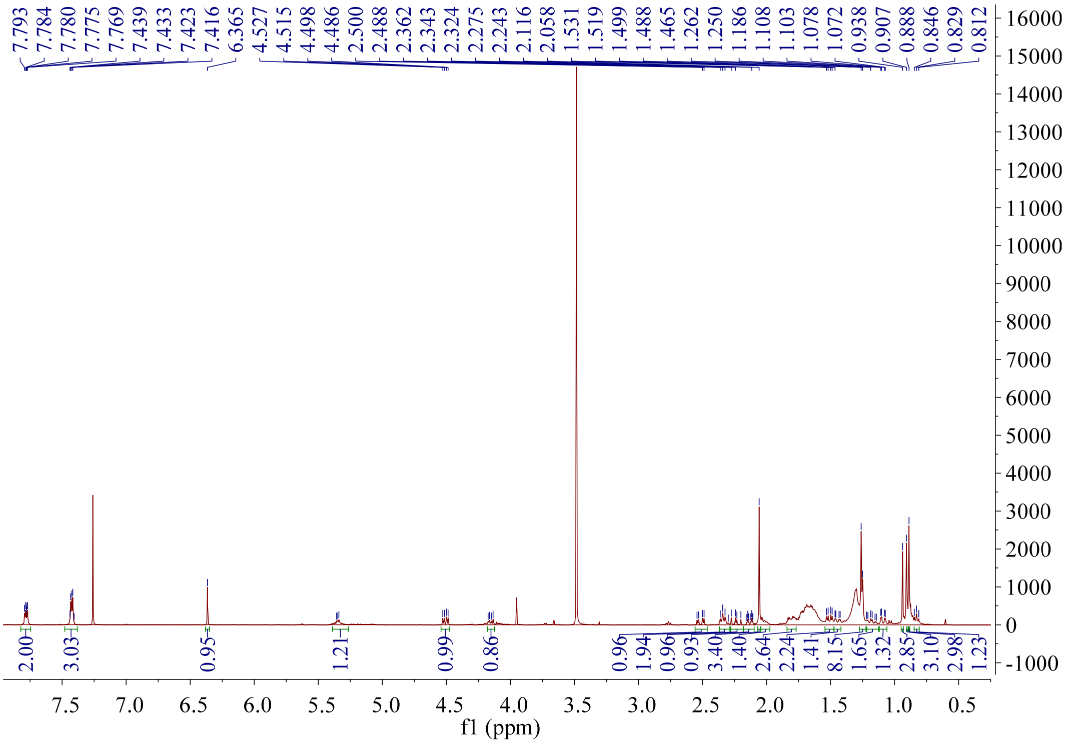

Figure S10. ^{1}H NMR spectrum of phenylpyropene C (**3**) in $CDCl_3$, 400 MHz.

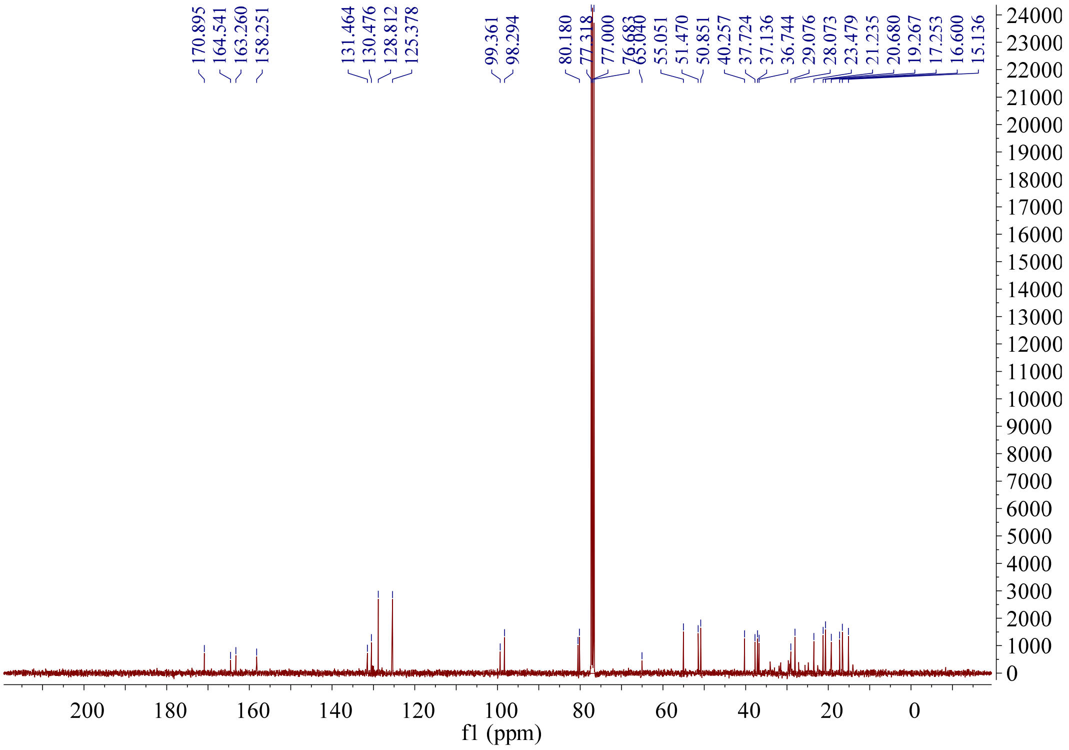

Figure S11. ^{13}C NMR spectrum of phenylpyropene C (**3**) in $CDCl_3$, 100 MHz.

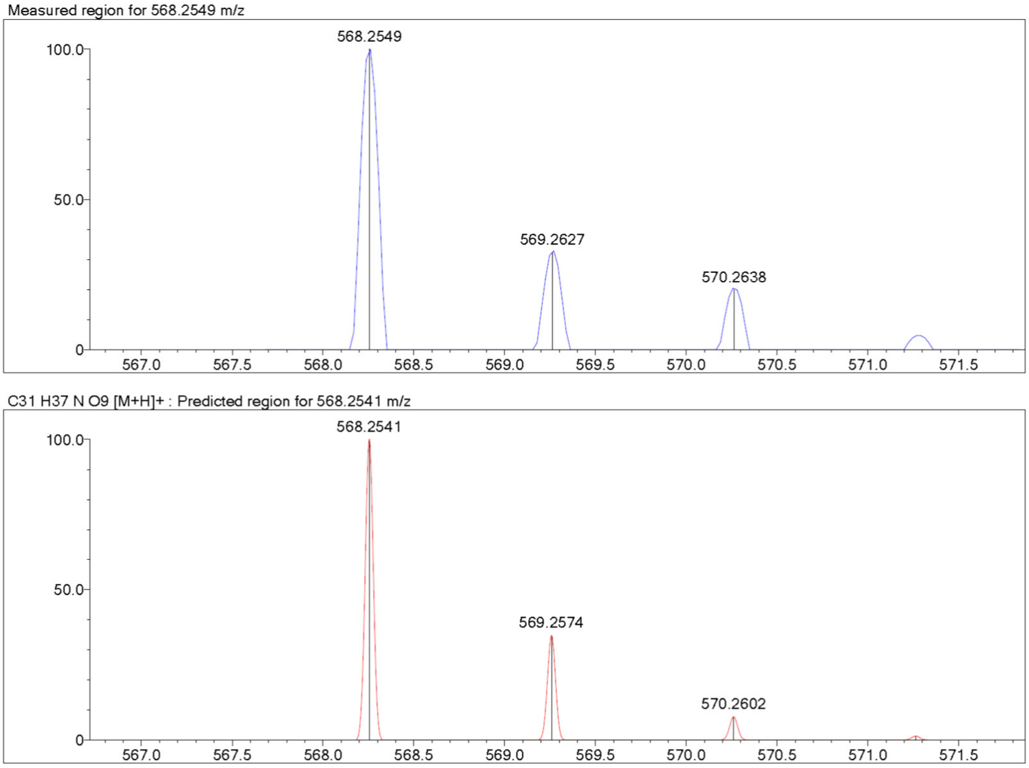

Rank	Score	Formula (M)	Ion	Meas. m/z	Pred. m/z	Df. (mDa)	Df. (ppm)	Iso	DBE
1	57.61	C31 H37 N O9	[M+H]+	568.2549	568.2541	0.8	1.41	58.20	14.0

Figure S12. HR(+)ESIMS spectrum of 13-dehydroxylpyripyropene A (**4**).

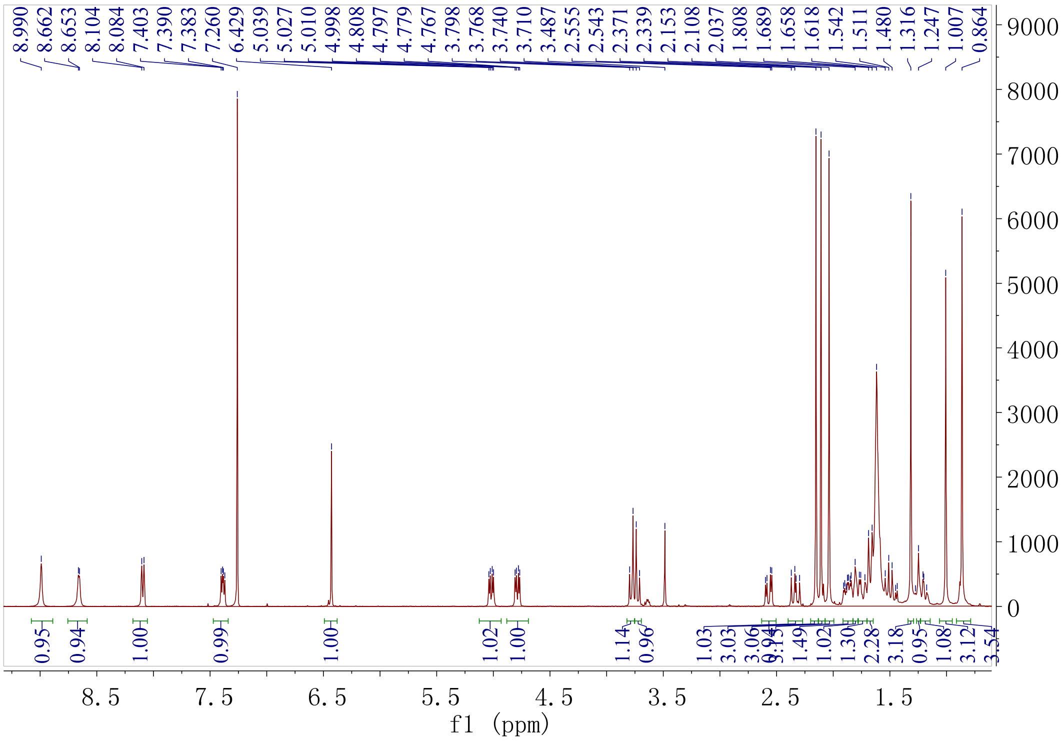

Figure S13. ^{1}H NMR spectrum of 13-dehydroxylpyripyropene A (**4**) in $CDCl_3$, 400 MHz.

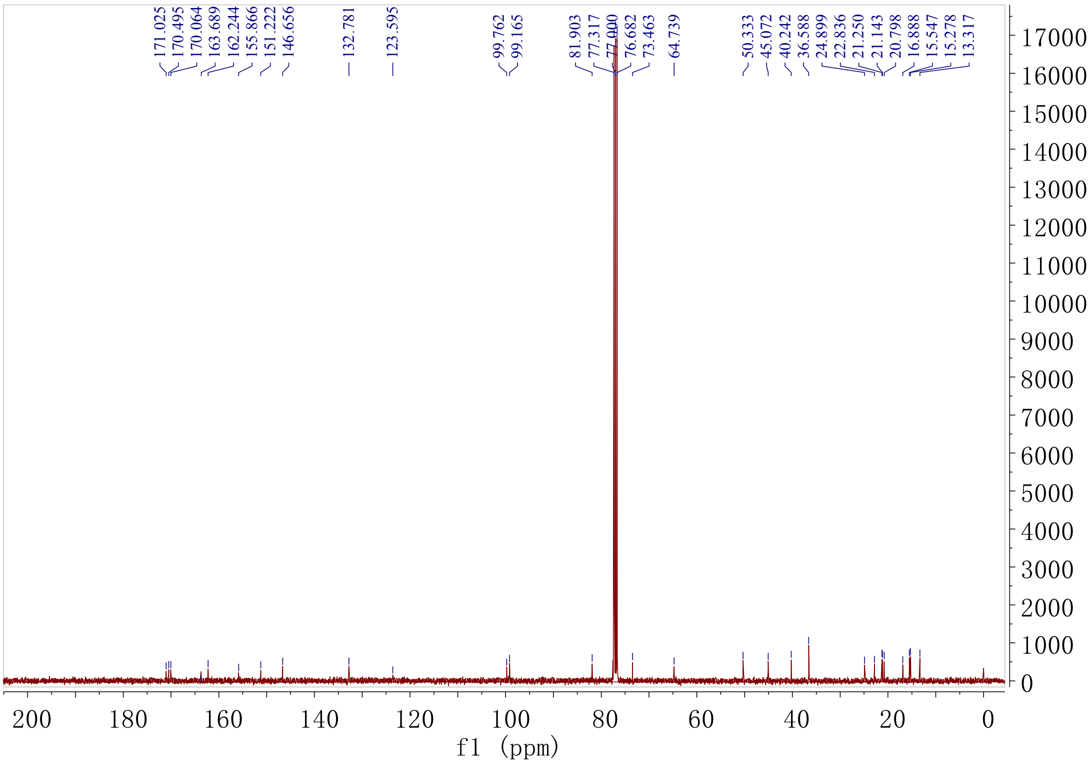

Figure S14. ^{13}C NMR spectrum of 13-dehydroxylpyripyropene A (**4**) in $CDCl_3$, 100 MHz.

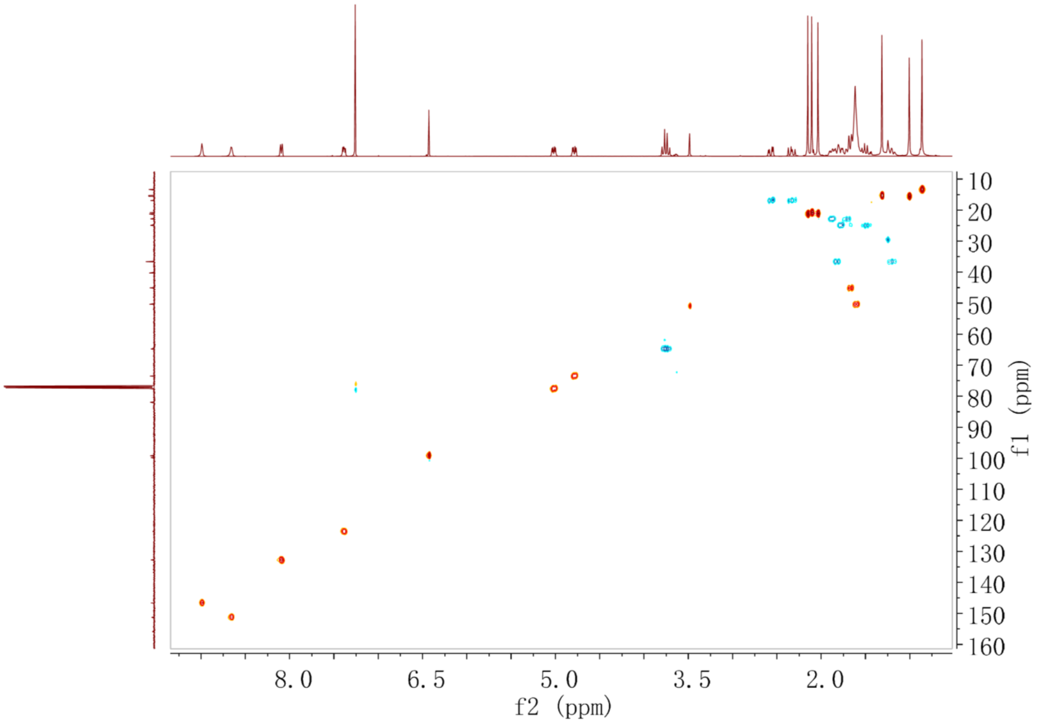

Figure S15. HSQC spectrum of 13-dehydroxylpyripyropene A (**4**).

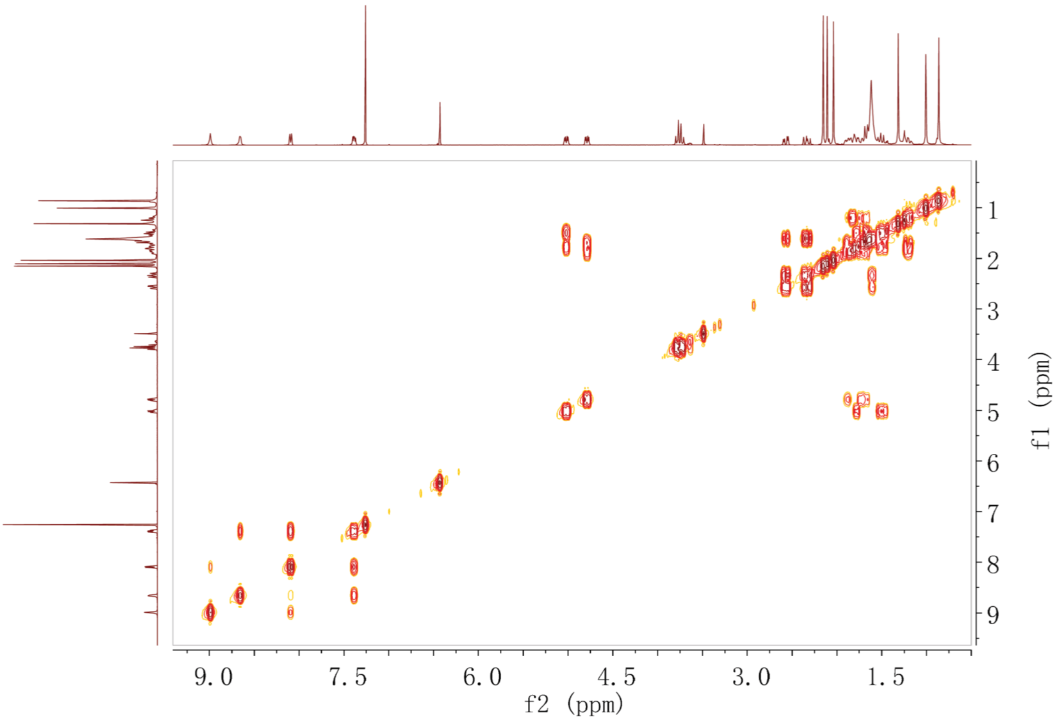

Figure S16. ^{1}H-^{1}H COSY spectrum of13-dehydroxylpyripyropene A (**4**).

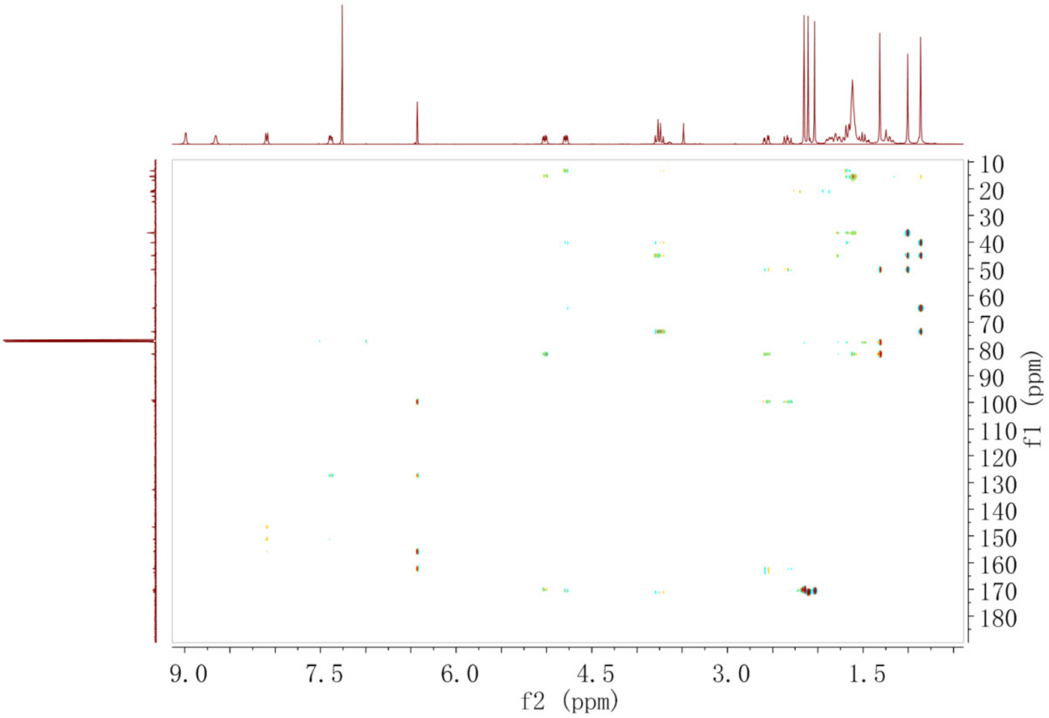

Figure S17. HMBC spectrum of 13-dehydroxylpyripyropene A (**4**).

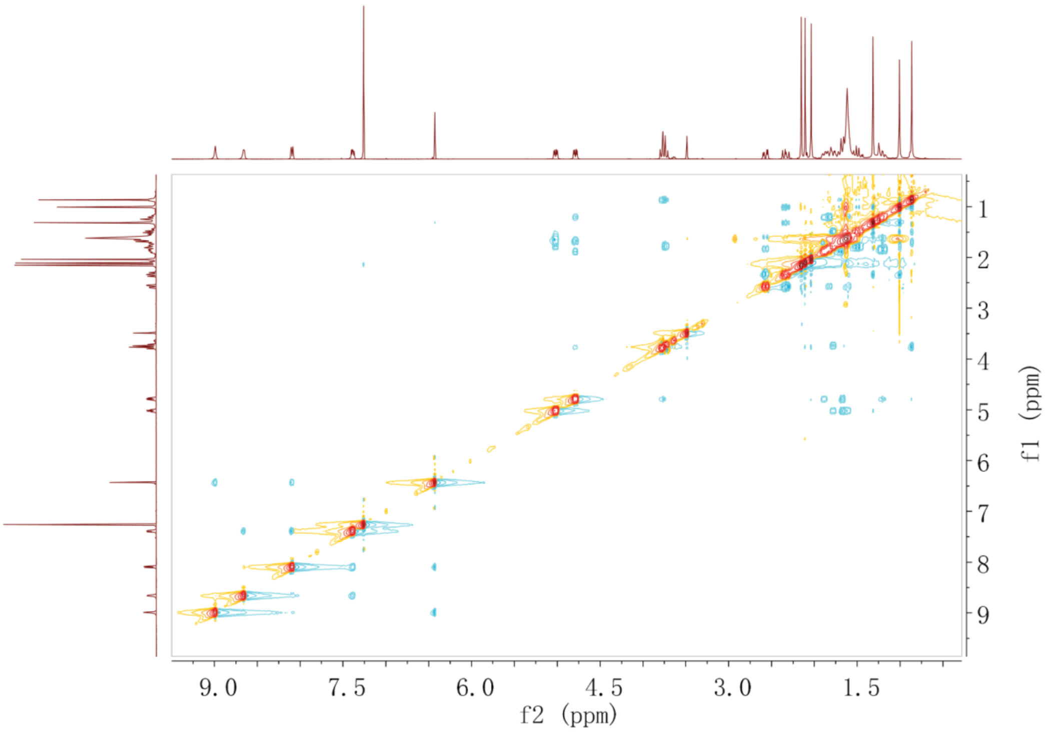

Figure S18. NOESY spectrum of 13-dehydroxylpyripyropene A (**4**).

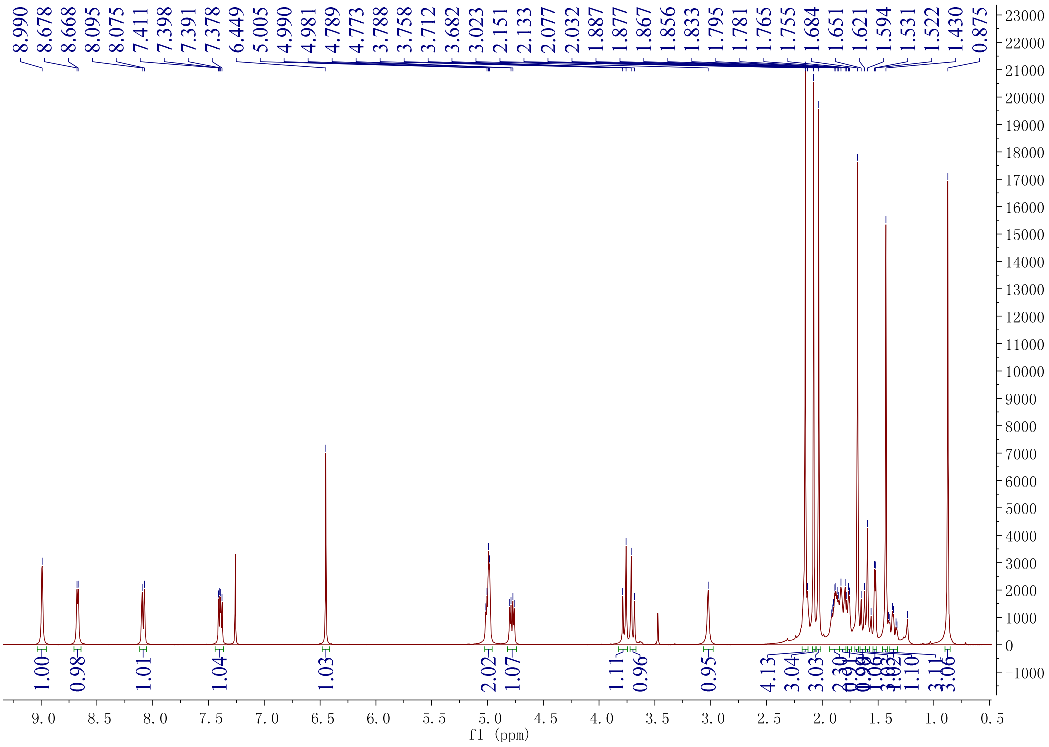

Figure S19. ^{1}H NMR spectrum of pyripyropene A (**5**) in $CDCl_3$, 400 MHz.

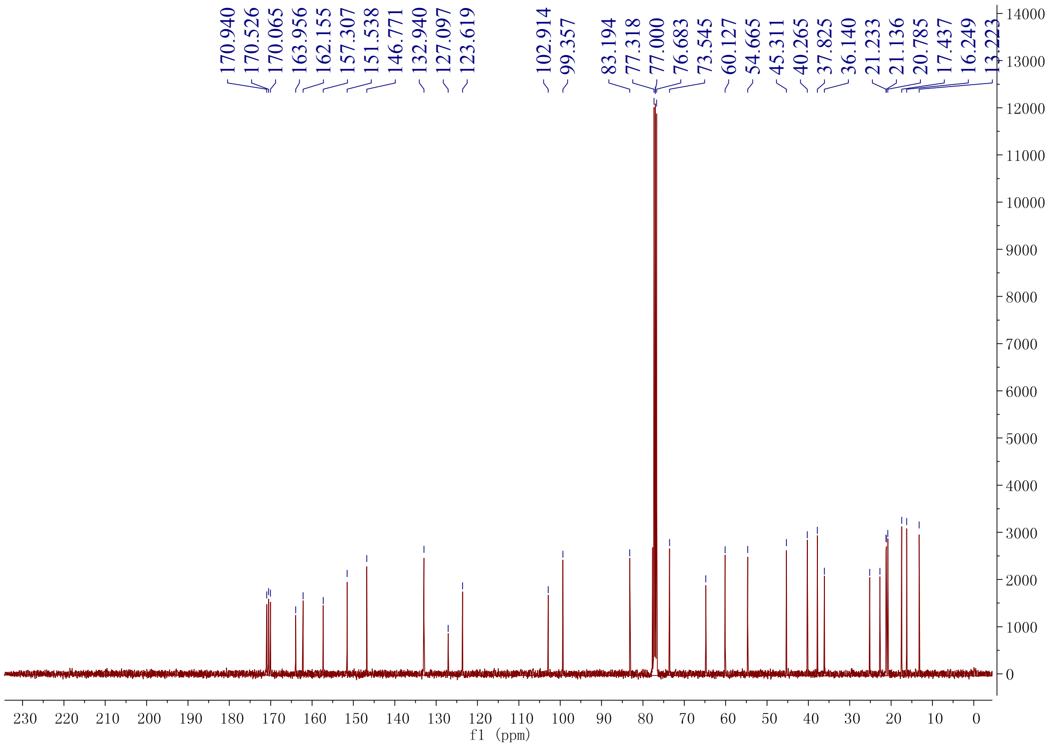

Figure S20. ^{13}C NMR spectrum of pyripyropene A (**5**) in $CDCl_3$, 100 MHz.

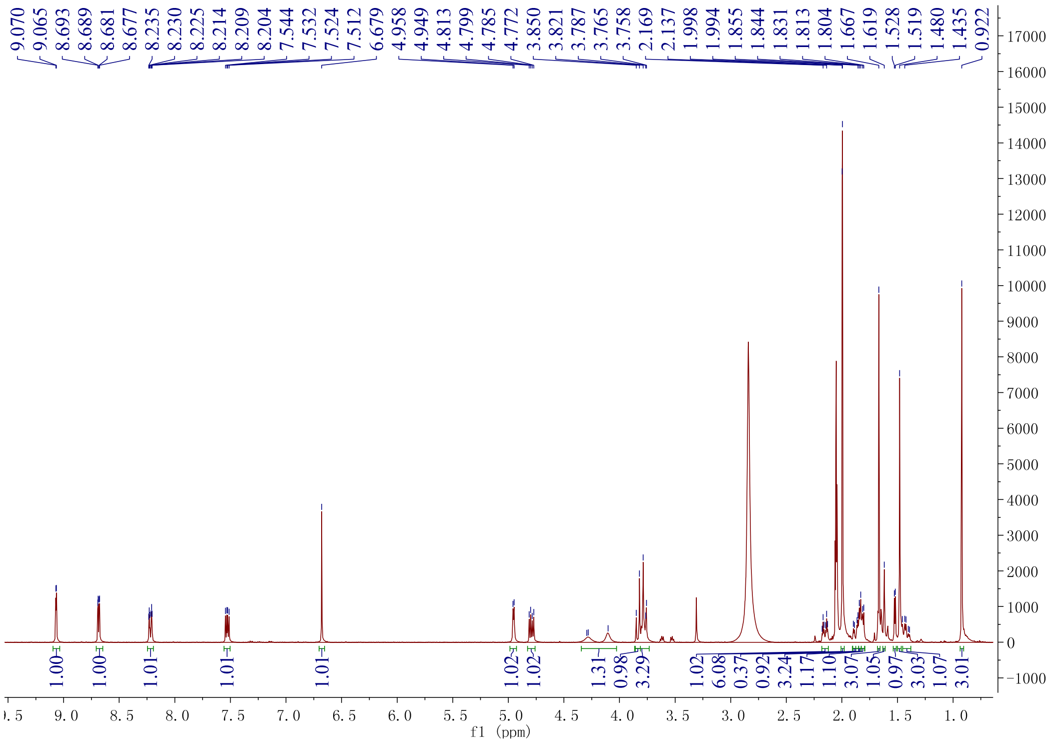

Figure S21. ^{1}H NMR spectrum of 7-deacetylpyripyropene A (**6**) in acetone-d_6, 400 MHz.

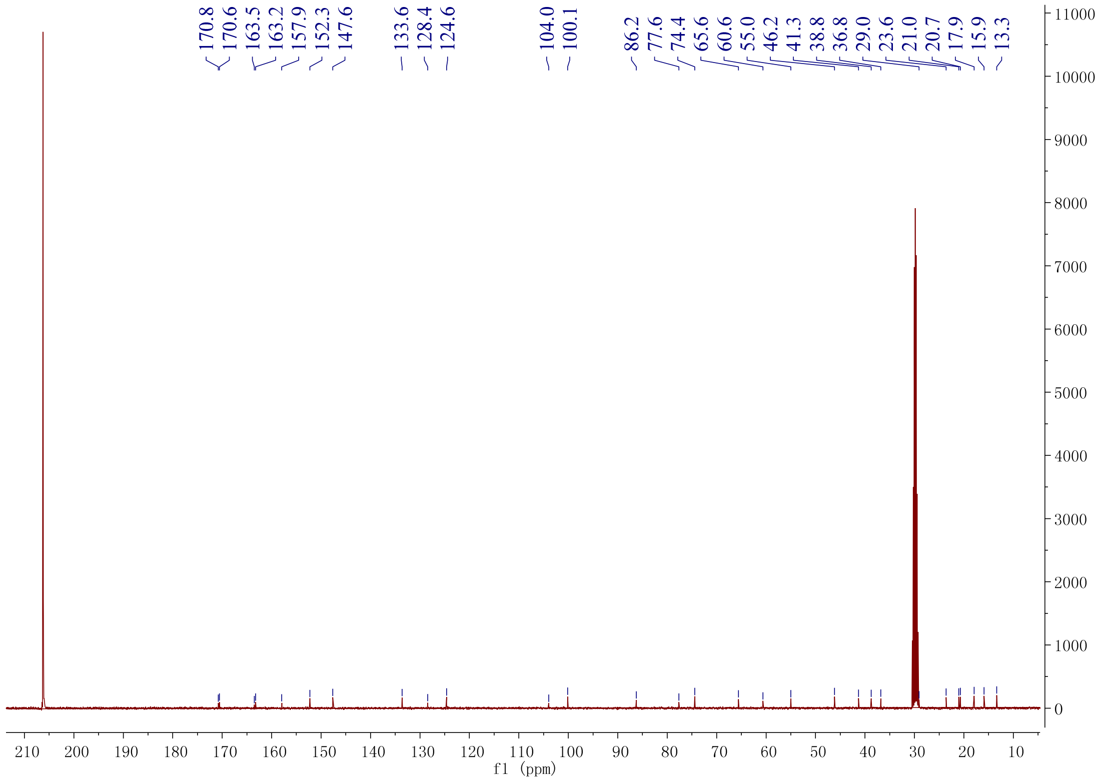

Figure S22. ^{13}C NMR spectrum of 7-deacetylpyripyropene A (**6**) in acetone-d_6, 100 MHz.

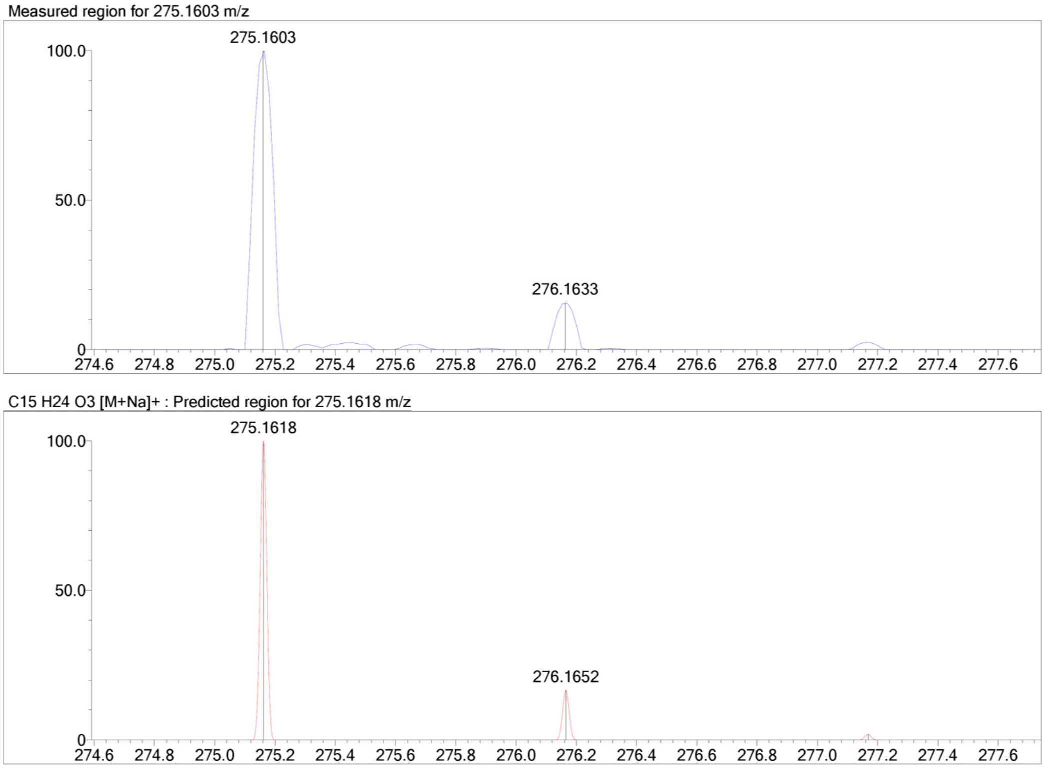

Rank	Score	Formula (M)	Ion	Meas. m/z	Pred. m/z	Df. (mDa)	Df. (ppm)	Iso	DBE
1	67.60	C15 H24 O3	[M+Na]+	275.1603	275.1618	-1.5	-5.45	79.06	4.0

Figure S23. HR(+)ESIMS spectrum of deacetylsequiterpene (**7**).

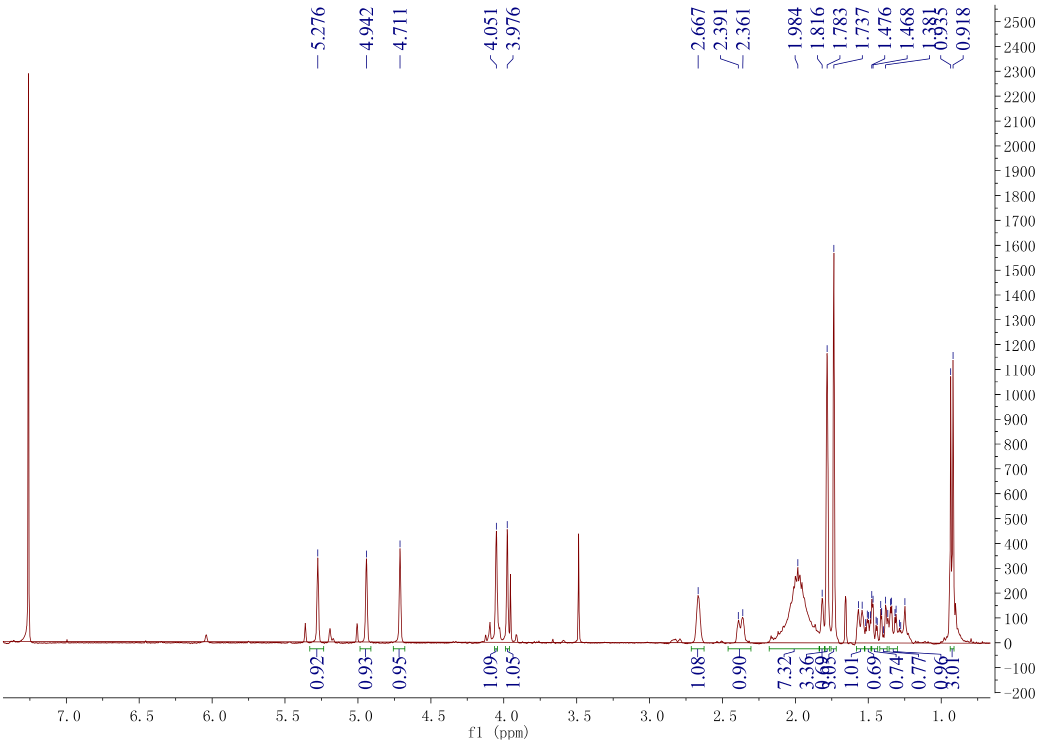

Figure S24. ^{1}H NMR spectrum of deacetylsequiterpene (**7**) in $CDCl_3$, 400 MHz.

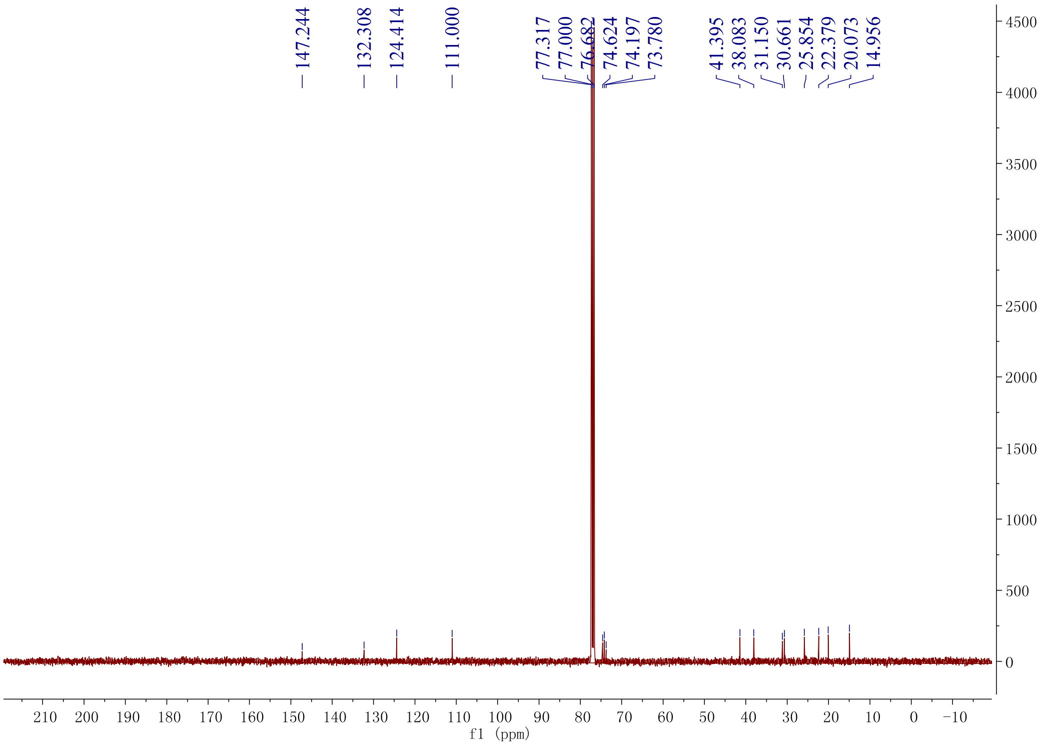

Figure S25. ^{13}C NMR spectrum of deacetylsequiterpene (**7**) in $CDCl_3$, 100 MHz.

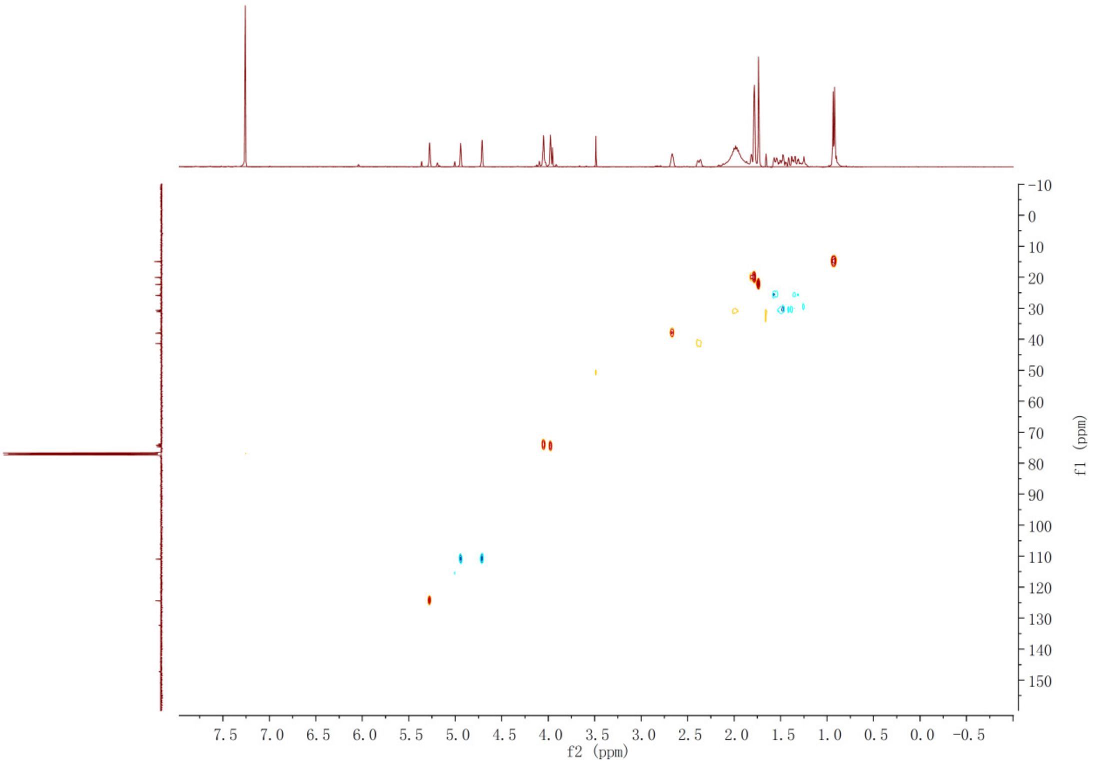

Figure S26. HSQC spectrum of deacetylsequiterpene (**7**).

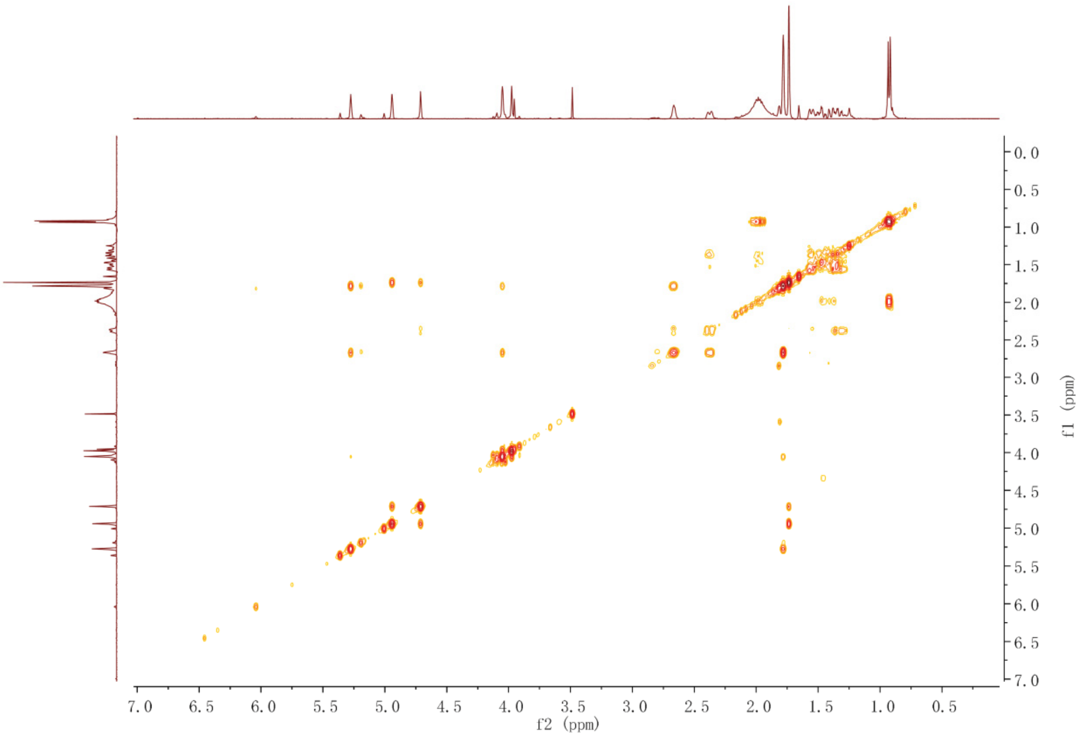

Figure S27. ^{1}H-^{1}H COSY spectrum of deacetylsequiterpene (**7**).

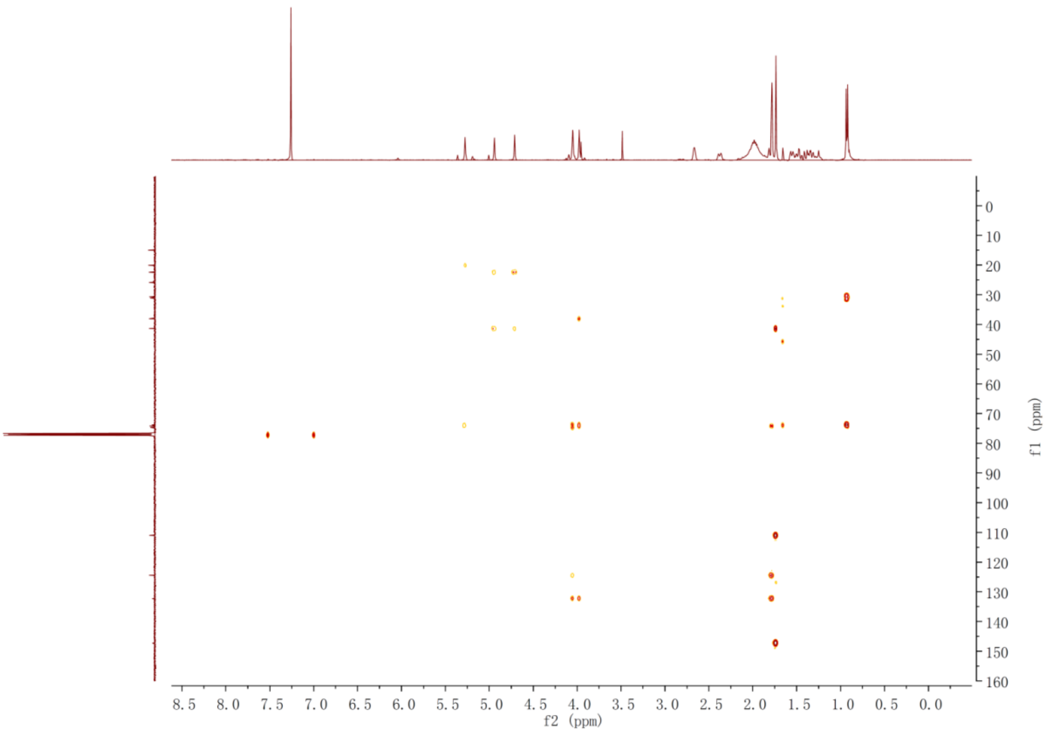

Figure S28. HMBC spectrum of deacetylsequiterpene (**7**).

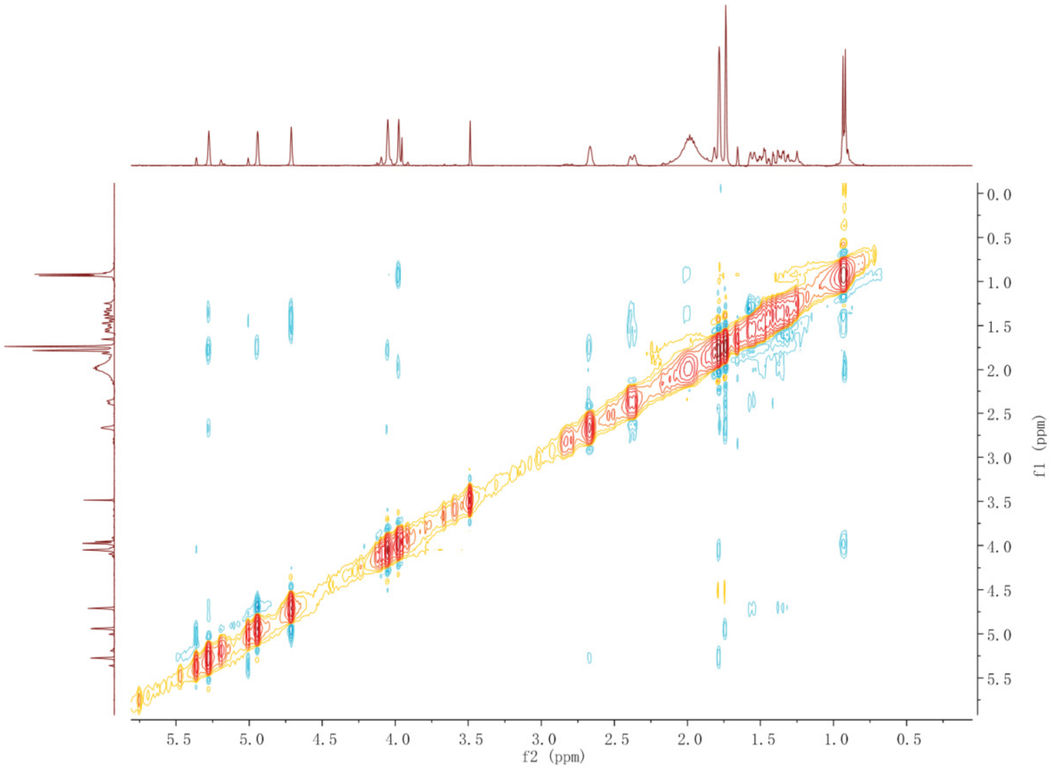

Figure S29. NOESY spectrum of deacetylsequiterpene (**7**).

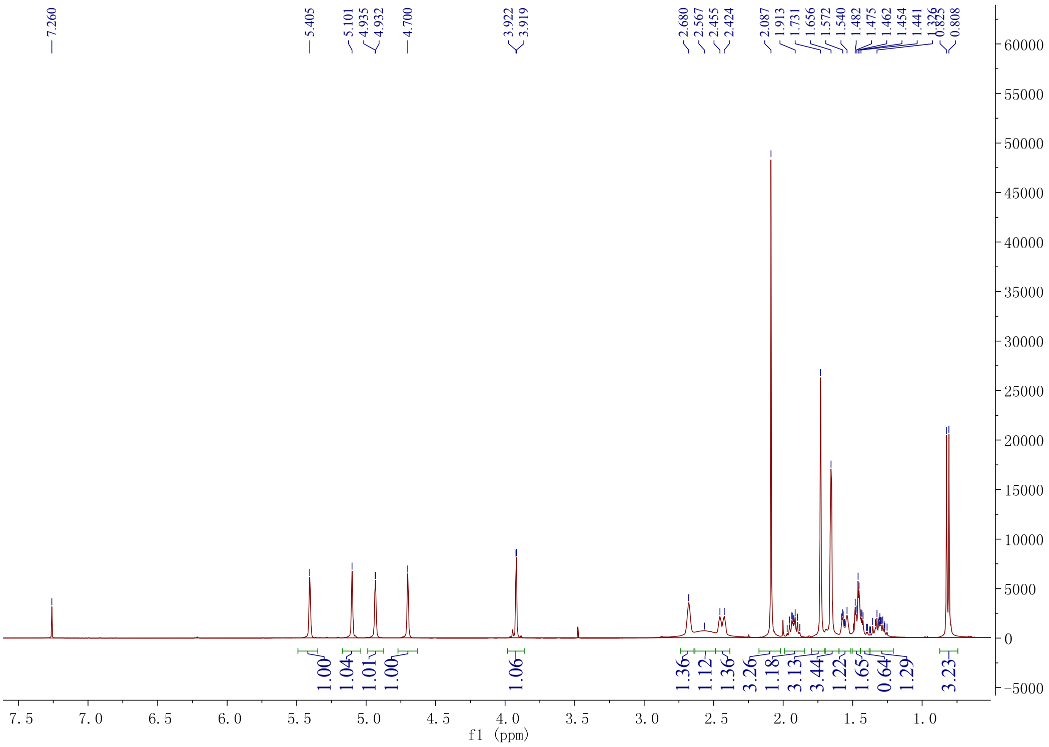

Figure S30. ^{1}H NMR spectrum of sesquiterpene (**8**) in $CDCl_3$, 400 MHz.

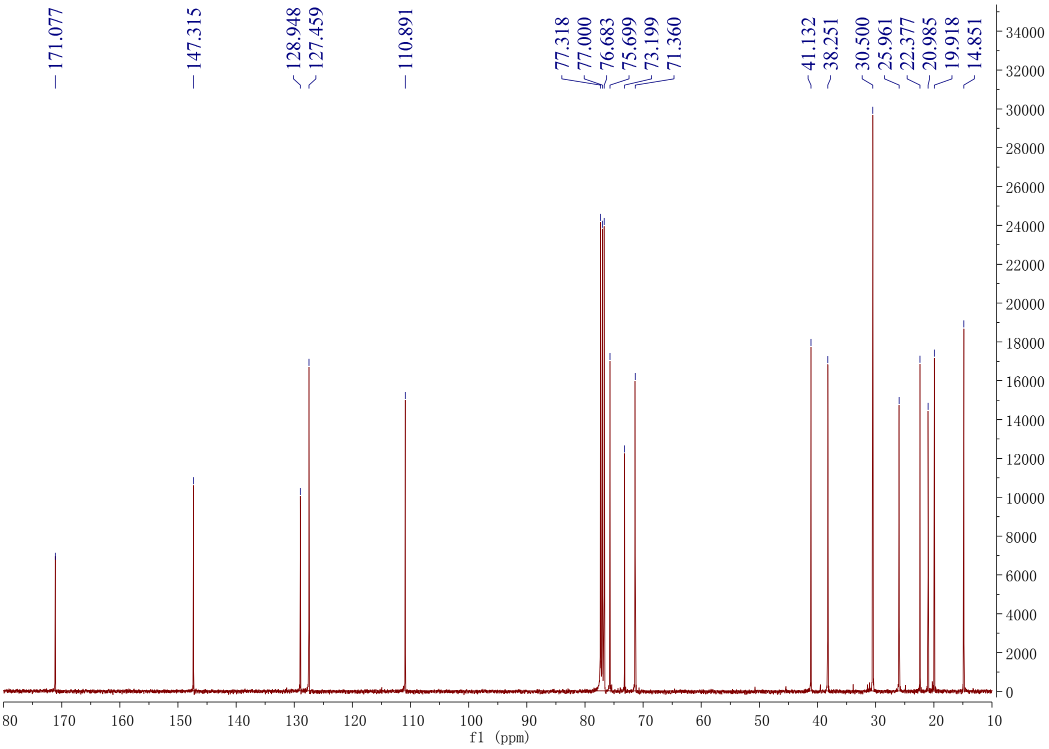

Figure S31. ^{13}C NMR spectrum of sesquiterpene (**8**) in $CDCl_3$, 100 MHz.

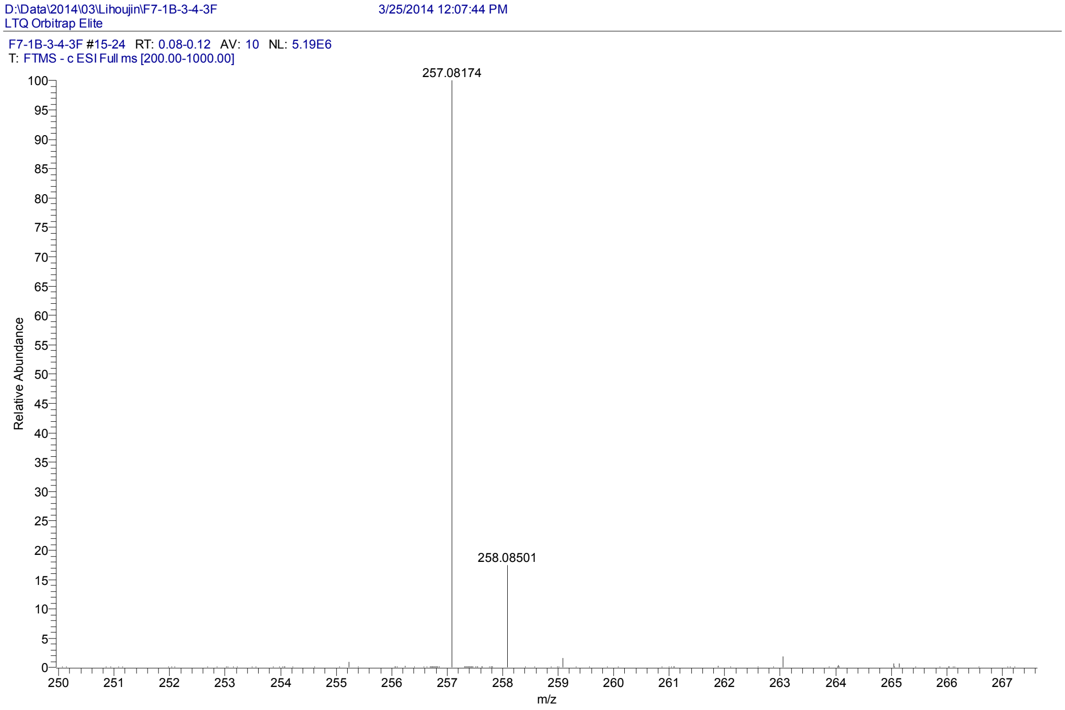

SPECTRUM-Simulation				
m*/*z	**Theo. Mass**	**Delta (ppm)**	**RDB Equiv.**	**Composition**
257.08174	257.08193	−0.75	9.5	C15 H13 O4

Figure S32. HR(-)ESIMS spectrum of 5-formly-6-hydroxy-8-isopro-pyl-2-naphthoic acid (**9**).

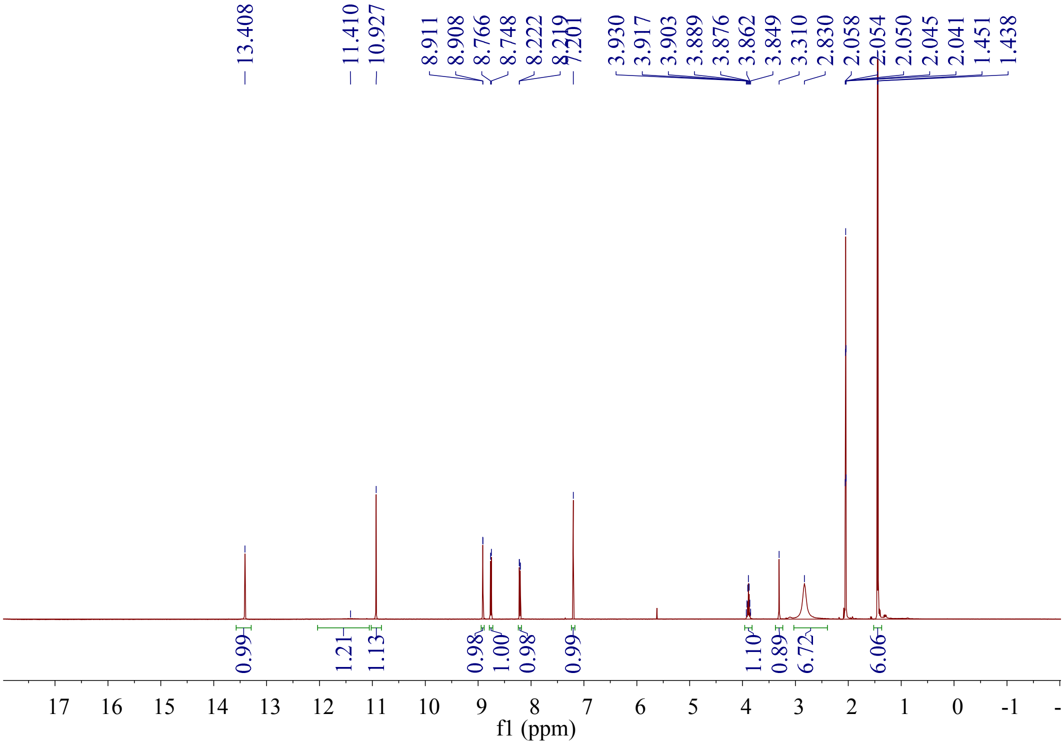

Figure S33. ¹H NMR spectrum of 5-formly-6-hydroxy-8-isopro-pyl-2-naphthoic acid (**9**) in Acetone-d_6, 500MHz.

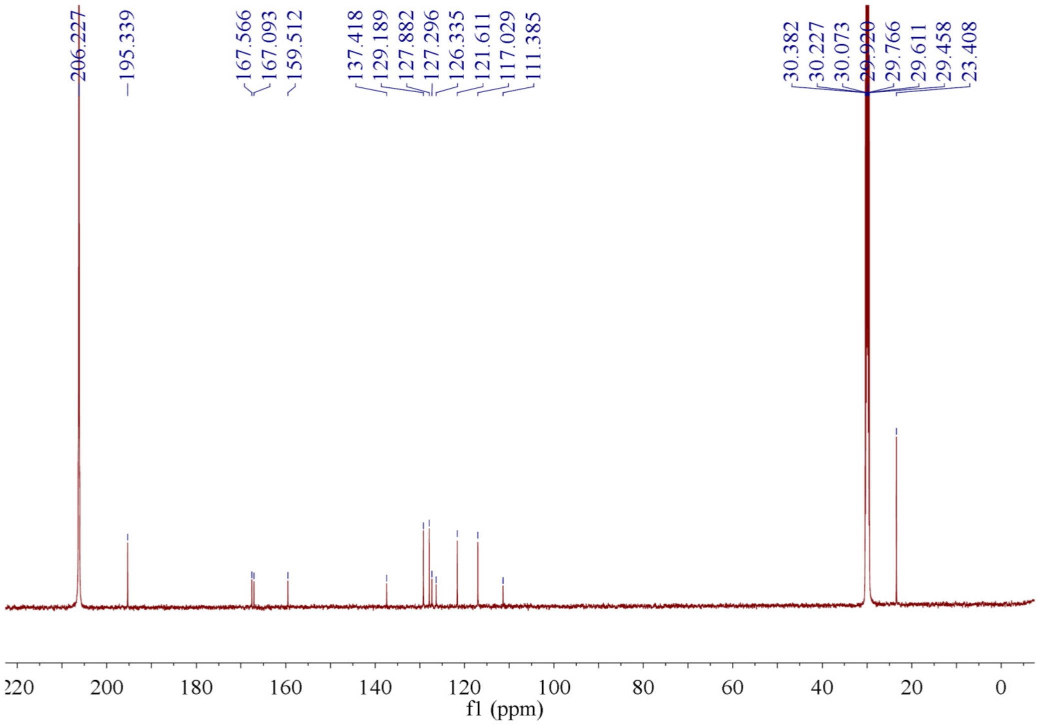

Figure S34. ^{13}C NMR spectrum of 5-formly-6-hydroxy-8-isopro-pyl-2-naphthoic acid (**9**) in Acetone-d_6, 125 MHz.

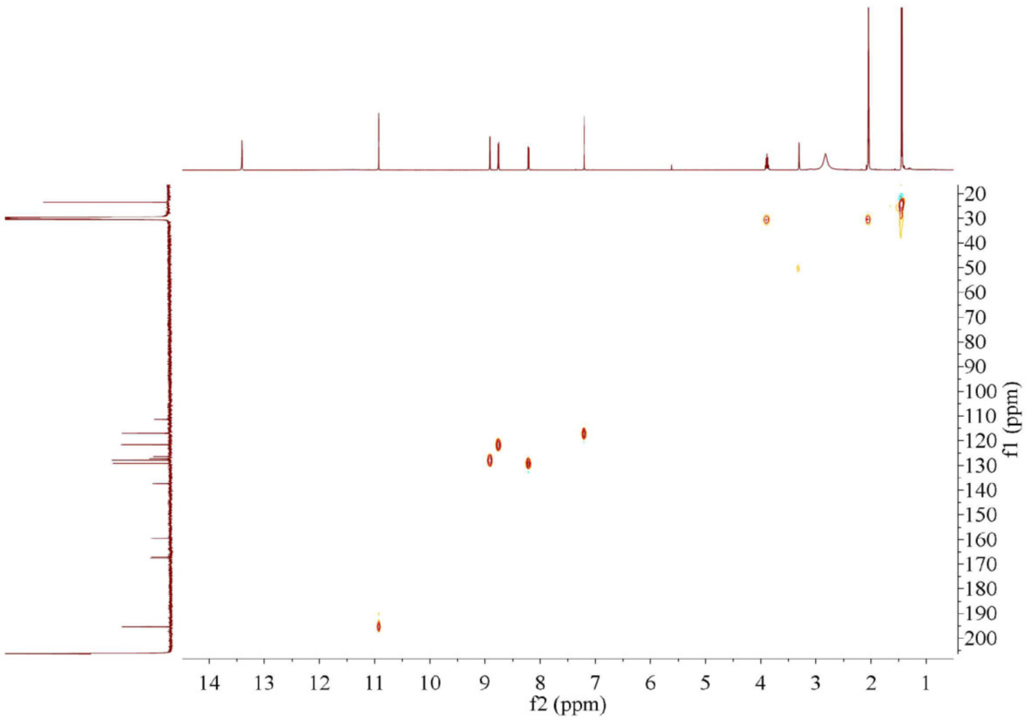

Figure S35. HSQC spectrum of 5-formly-6-hydroxy-8-isopro-pyl-2-naphthoic acid (**9**).

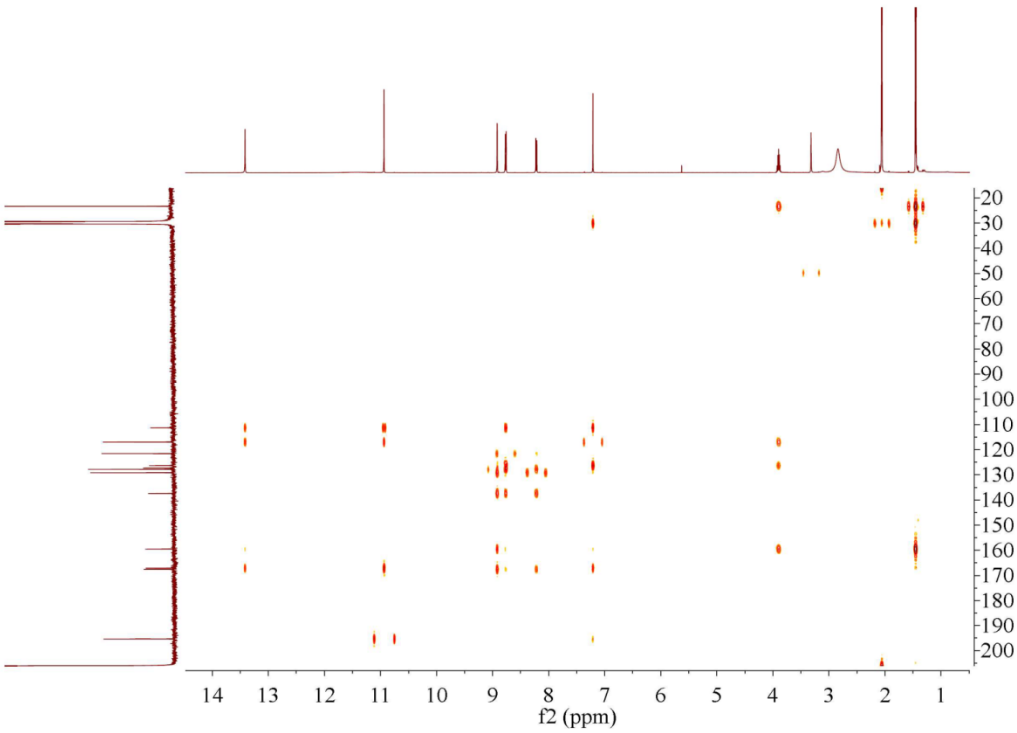

Figure S36. HMBC spectrum of 5-formly-6-hydroxy-8-isopro-pyl-2-naphthoic acid (**9**).

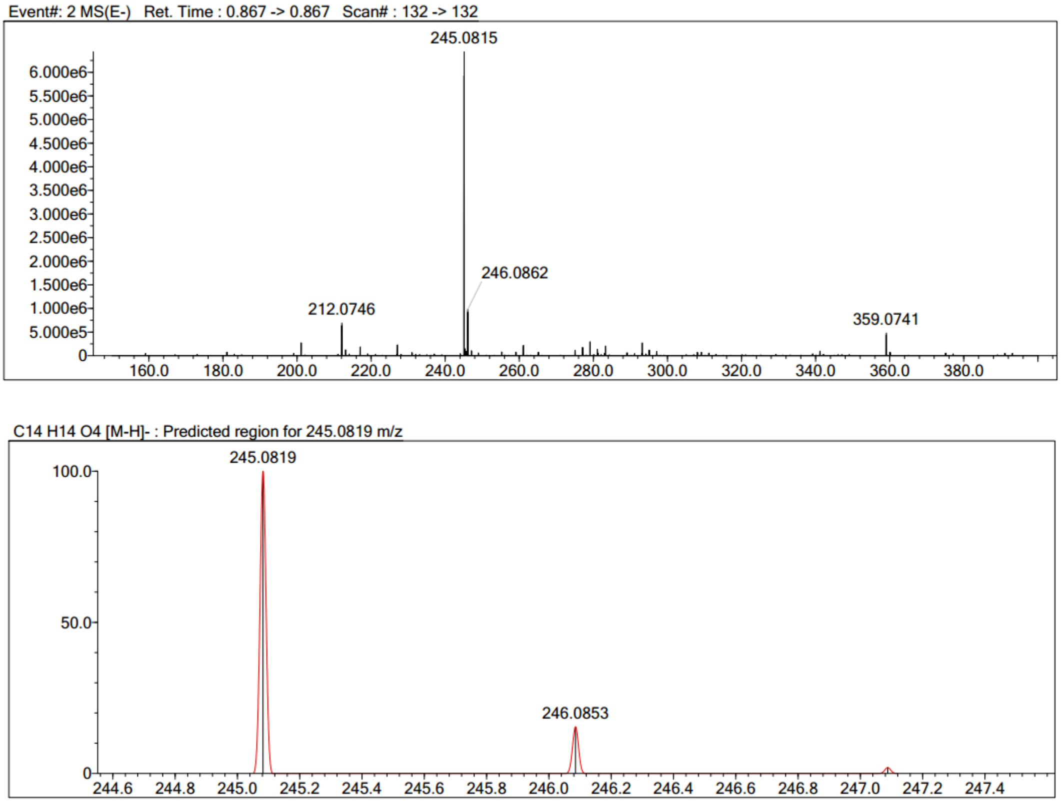

Rank	Score	Formula (M)	Ion	Meas. m/z	Pred. m/z	Df. (mDa)	Df. (ppm)	Iso	DBE
1	72.68	C14 H14 O4	[M-H]-	245.0815	245.0819	-0.4	-1.63	73.85	8.0

Figure S37. HRESIMS spectrum of **10**.

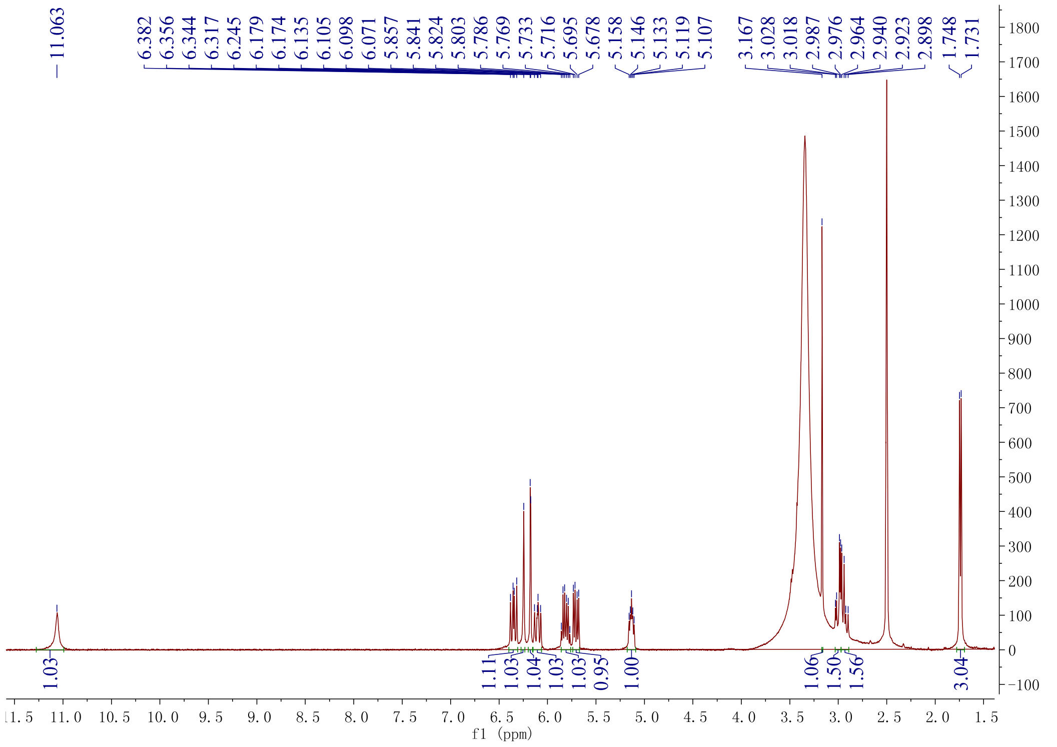

Figure S38. ^{1}H NMR spectrum of **10** in DMSO-d_6, 400 MHz.

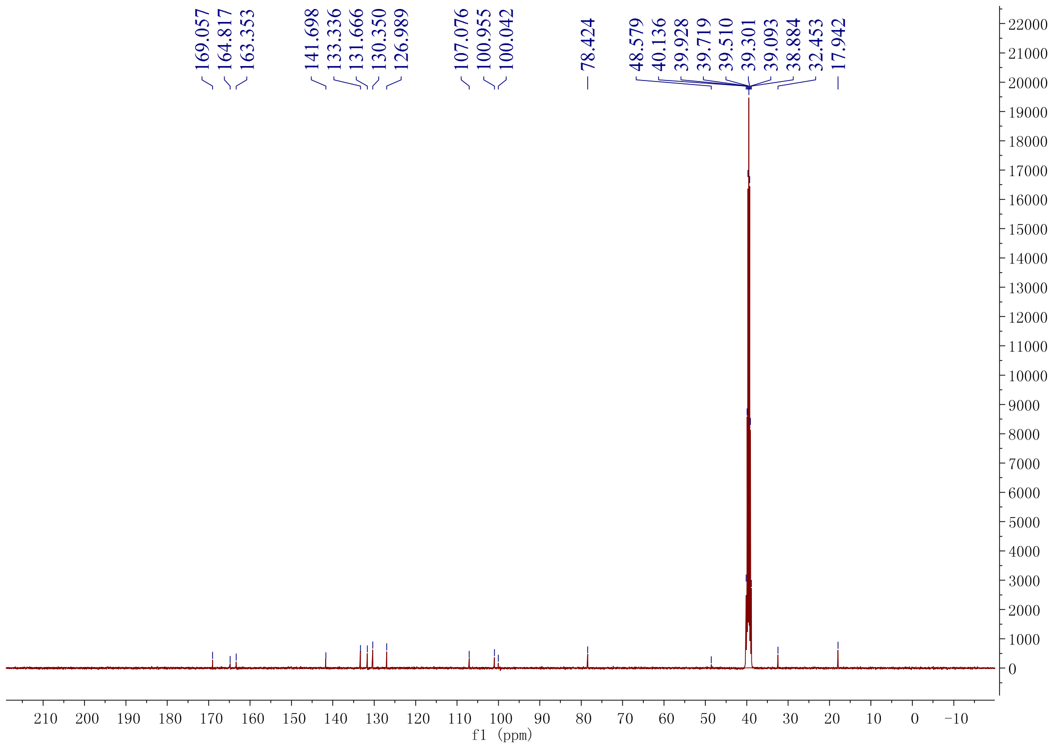

Figure S39. ^{13}C NMR spectrum of **10** in DMSO-d_6, 100 MHz.

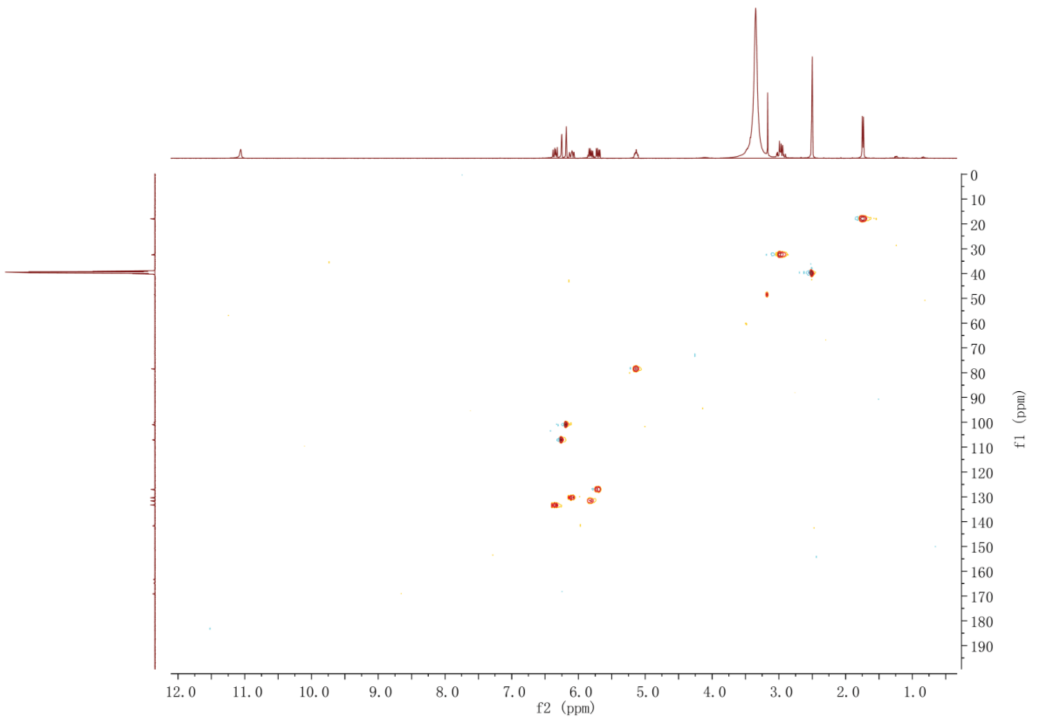

Figure S40. HSQC spectrum of 6,8-dihydroxy-3-((1*E*,3*E*)-penta-1,3-dien-1-yl)isochroman-1-one (**10**).

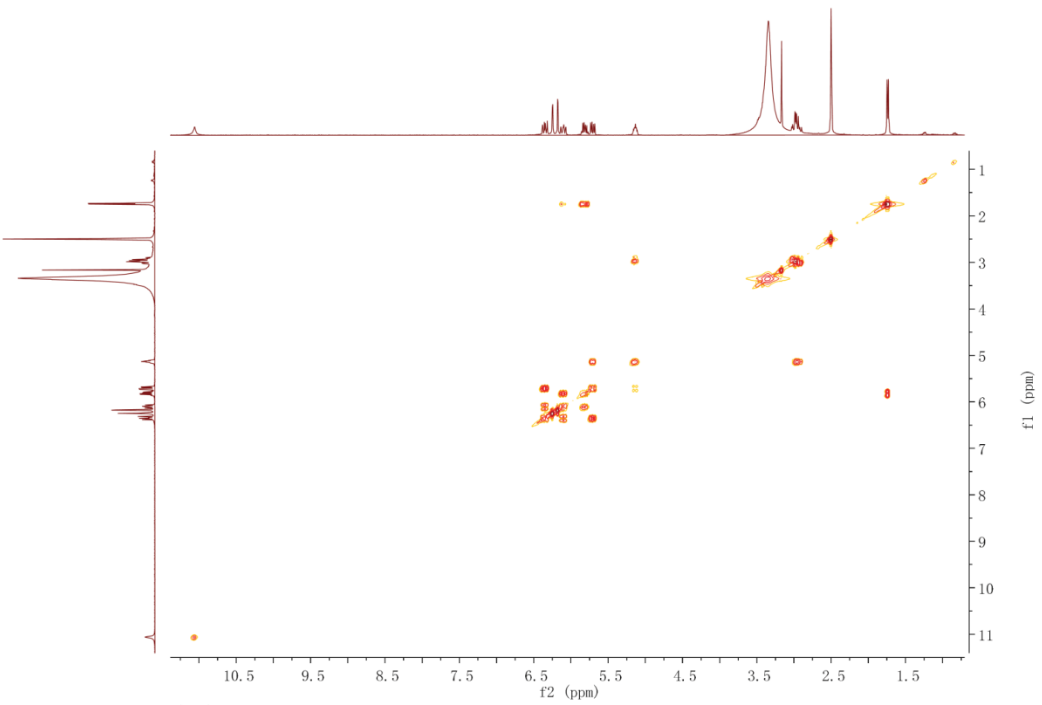

Figure S41. ^{1}H-^{1}H COSY spectrum of 6,8-dihydroxy-3-((1*E*,3*E*)-penta-1,3-dien-1-yl)isochroman-1-one (**10**).

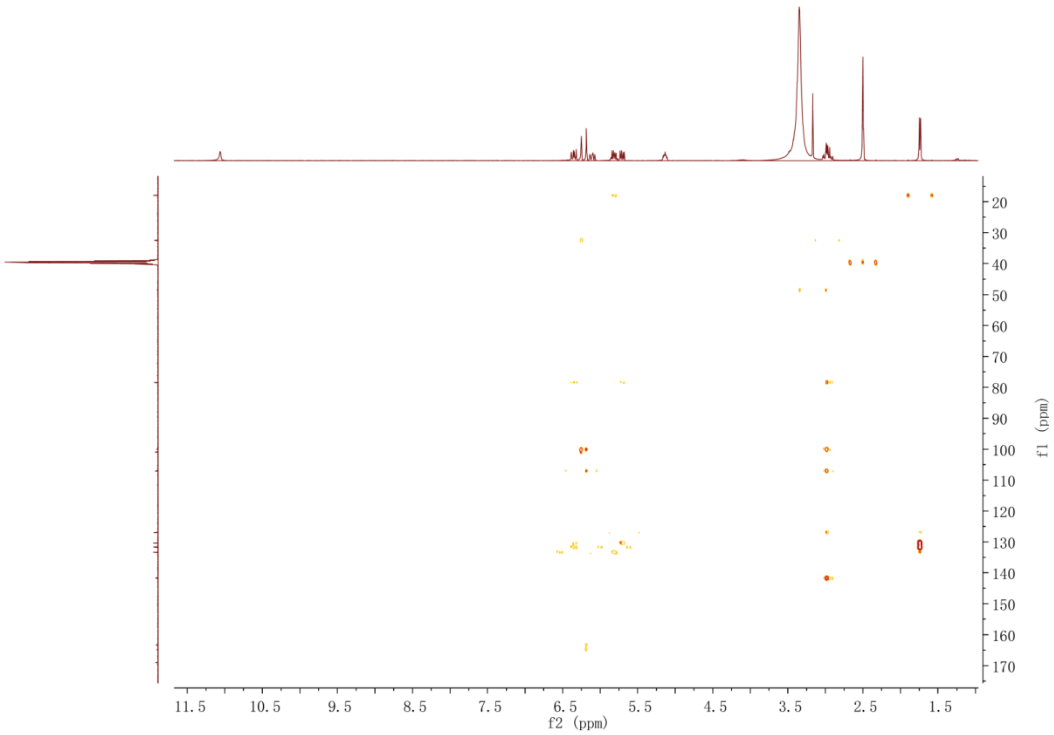

Figure S42. HMBC spectrum of 6,8-dihydroxy-3-((1*E*,3*E*)-penta-1,3-dien-1-yl)isochroman-1-one (**10**).

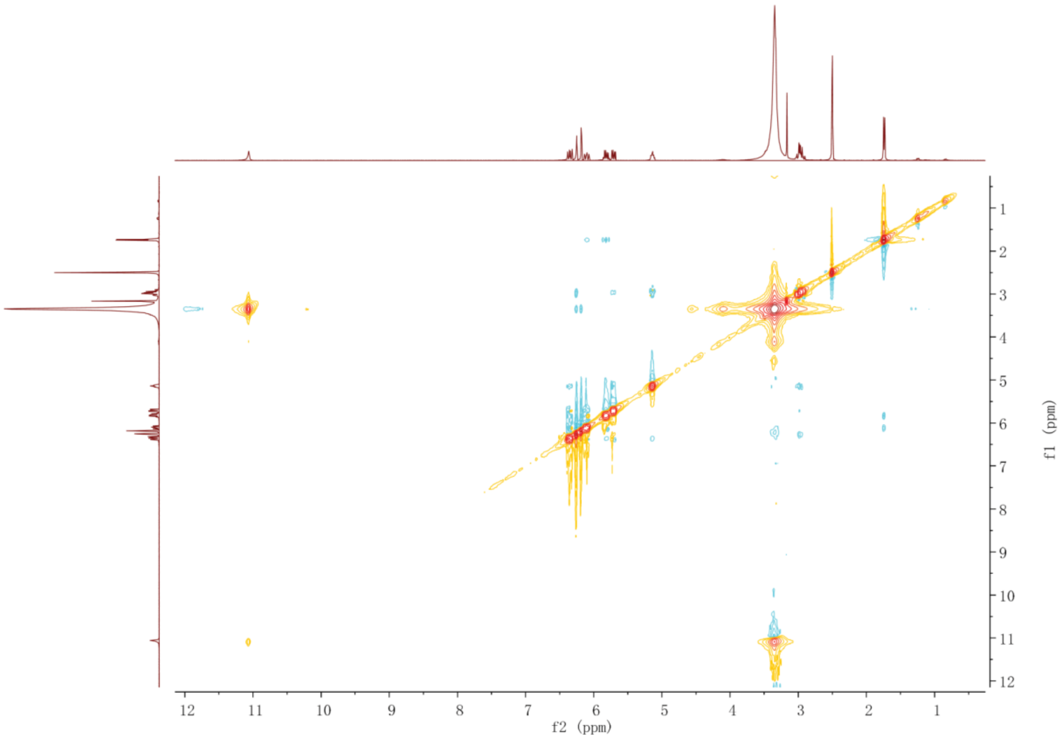

Figure S43. NOESY spectrum of 6,8-dihydroxy-3-((1*E*,3*E*)-penta-1,3-dien-1-yl)isochroman-1-one (**10**).

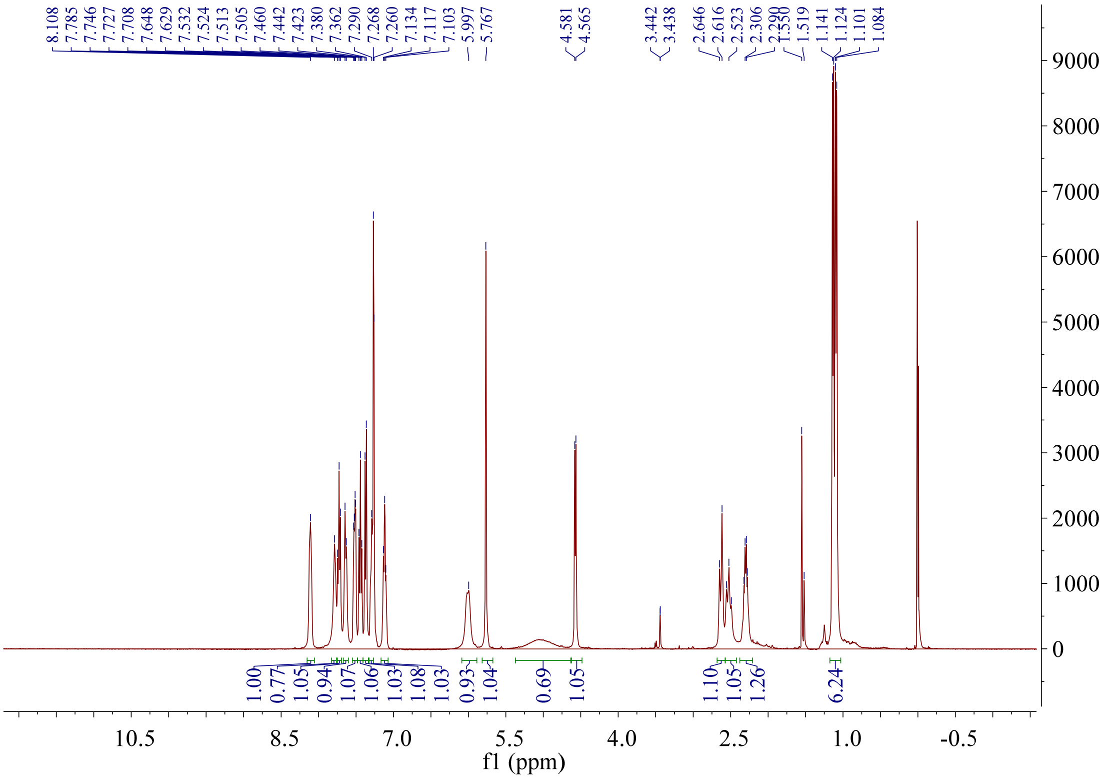

Figure S44. ^{1}H NMR spectrum of isochaetominine C (**11**) in $CDCl_3$, 400 MHz.

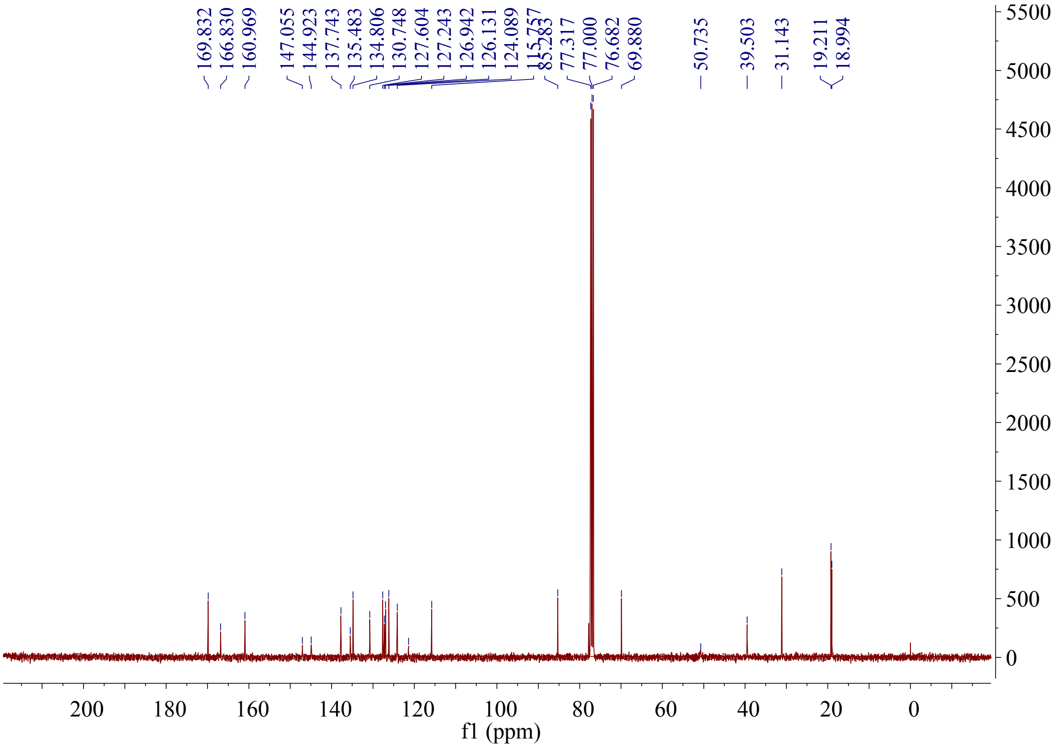

Figure S45. ^{13}C NMR spectrum of isochaetominine C (**11**) in $CDCl_3$, 100 MHz.

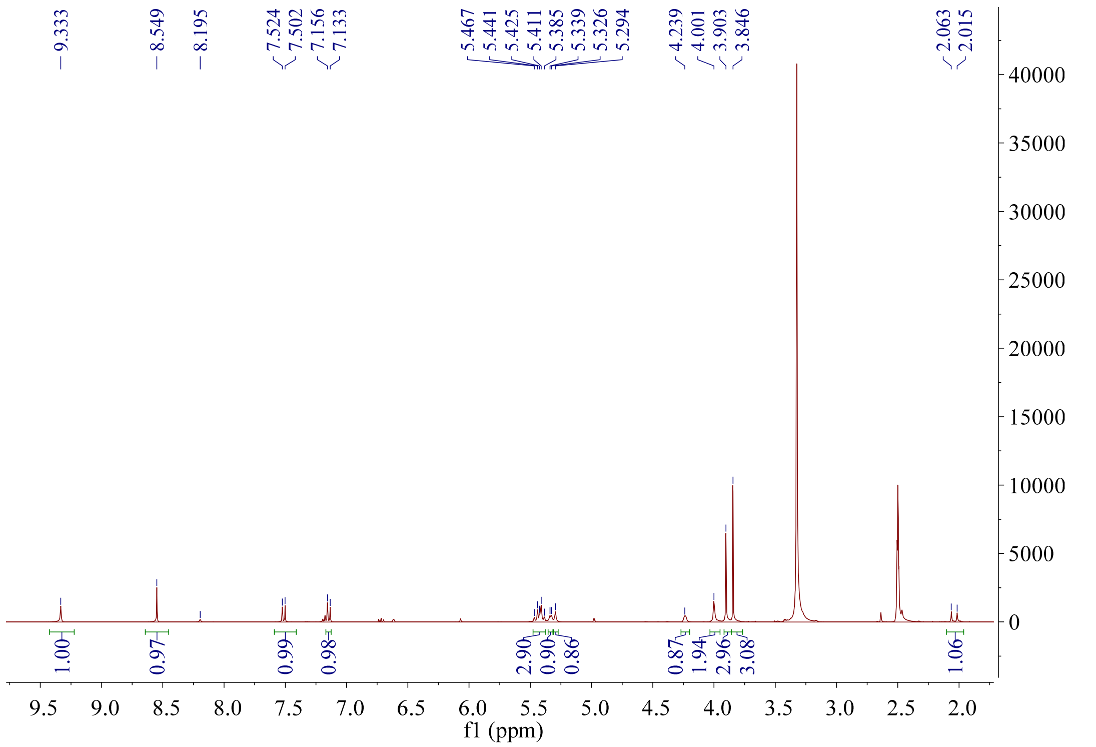

Figure S46. ^{1}H NMR spectrum of trichodermamide A (**12**) in DMSO-d_6, 400 MHz.

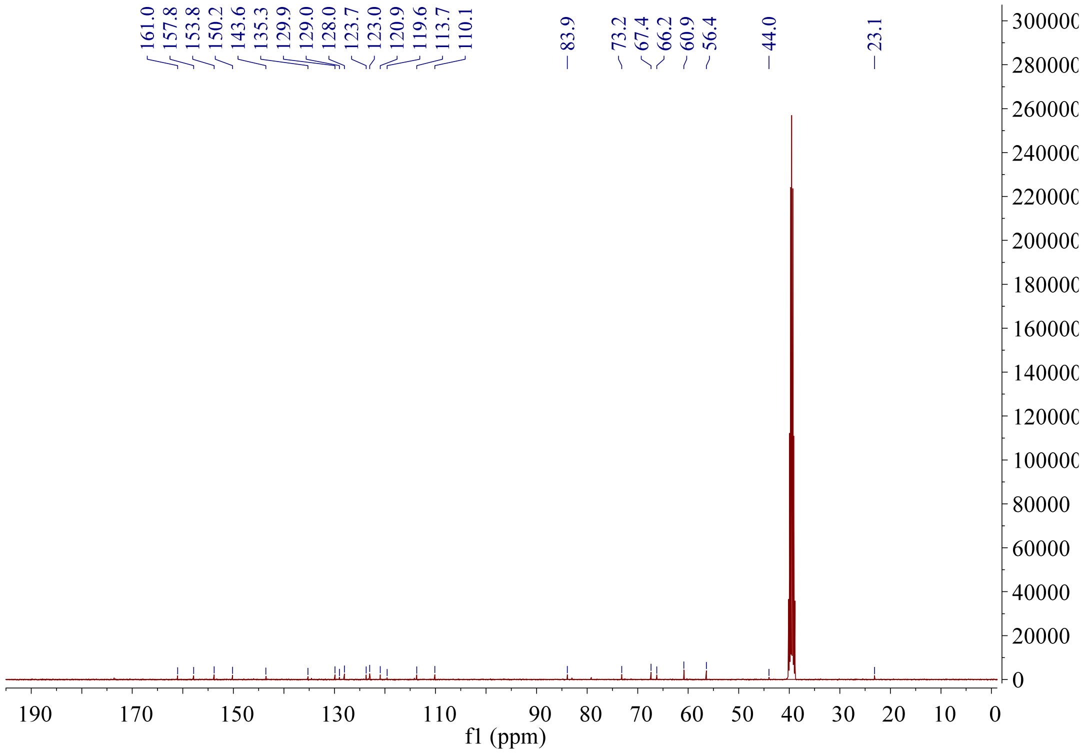

Figure S47. ^{13}C NMR spectrum of trichodermamide A (**12**) in DMSO-d_6, 100 MHz.

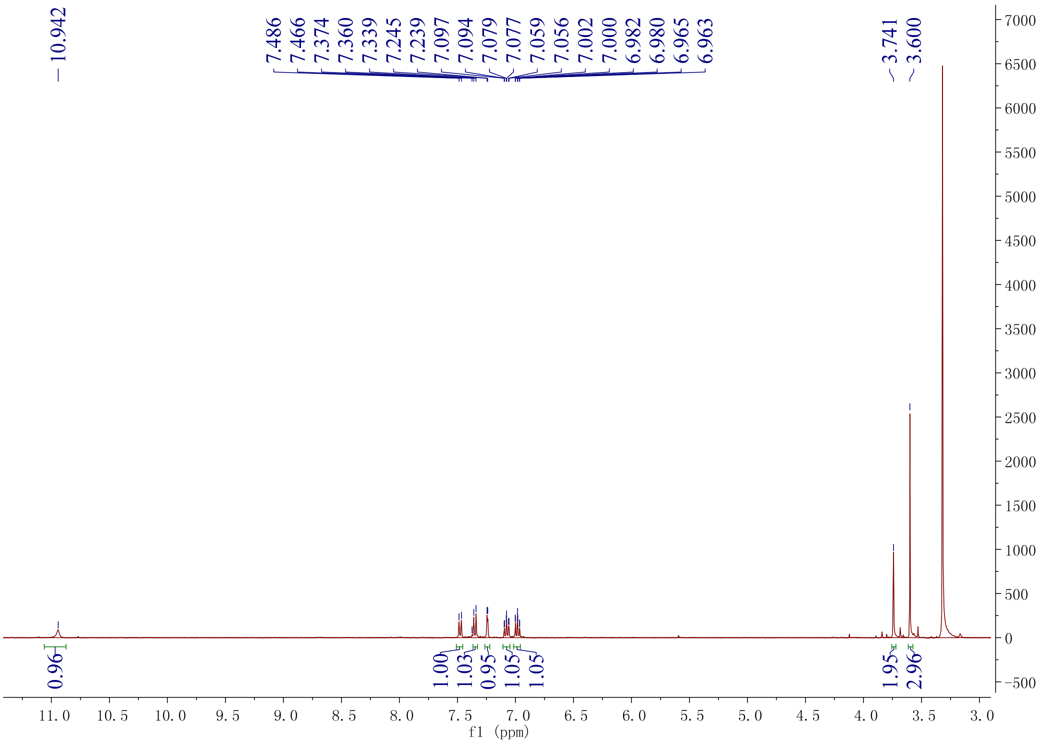

Figure S48. ^{1}H NMR spectrum of indolyl-3-acetic acid methyl ester (**13**) in DMSO-d_6, 400 MHz.

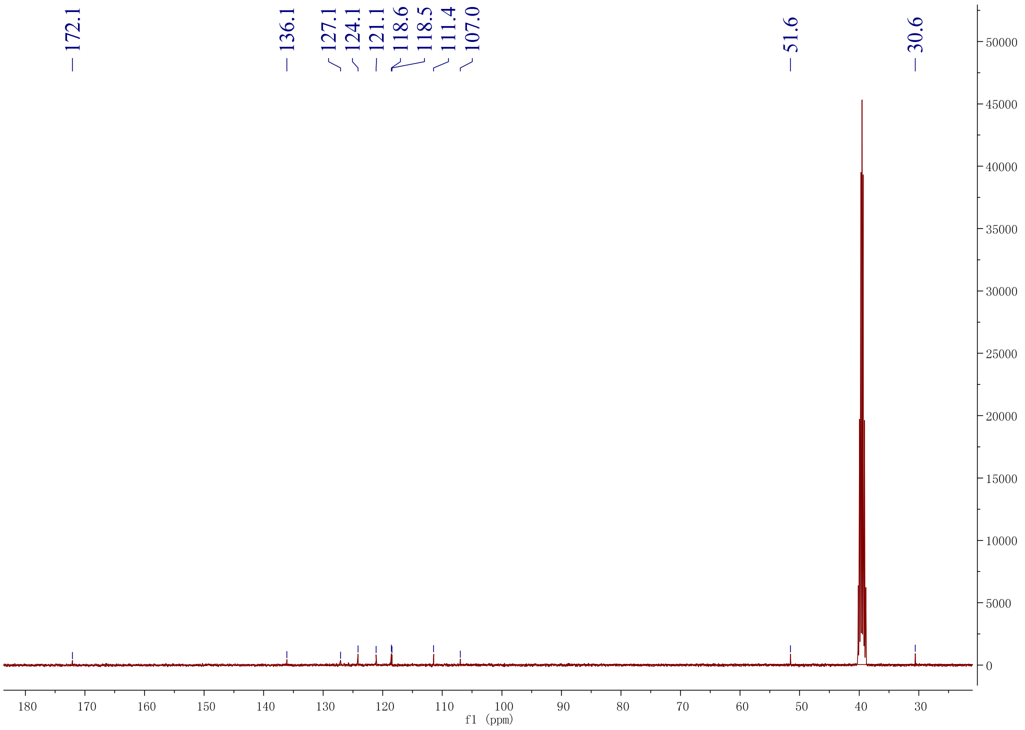

Figure S49. ^{13}C NMR spectrum of indolyl-3-acetic acid methyl ester (**13**) in DMSO-d_6, 100 MHz.

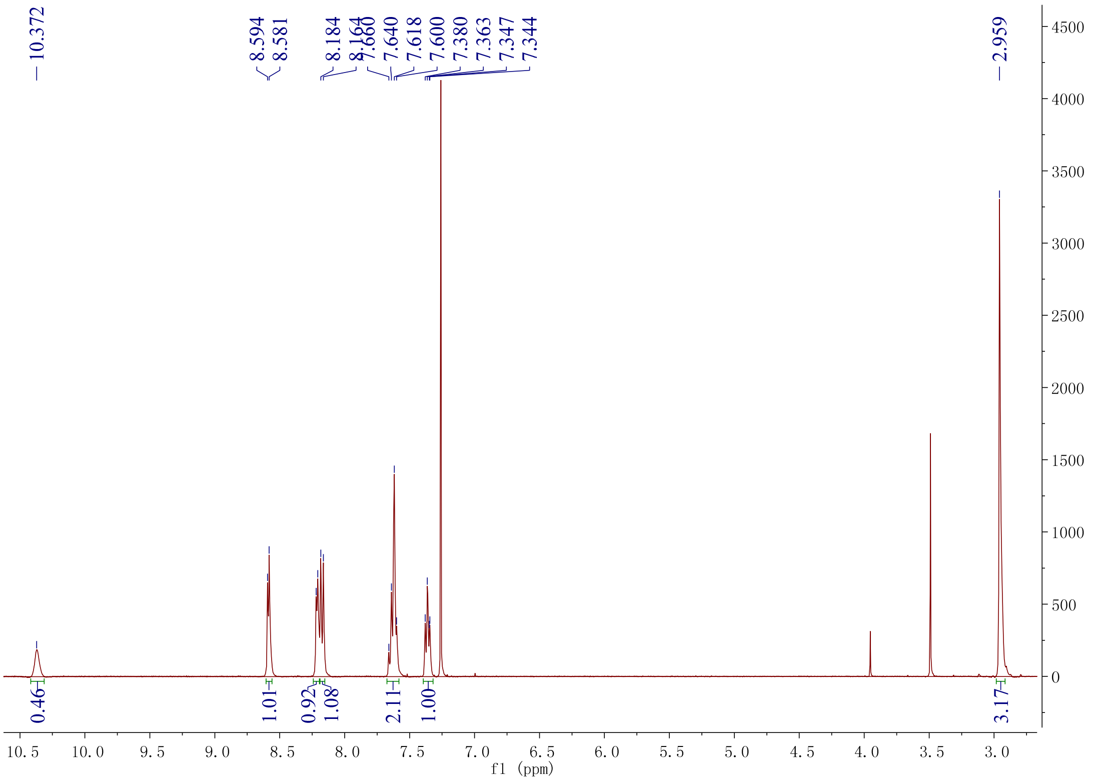

Figure S50. ^{1}H NMR spectrum of 1-(9*H*-β-carbolin-1-yl)-ethanone (**14**) in $CDCl_3$, 400 MHz.

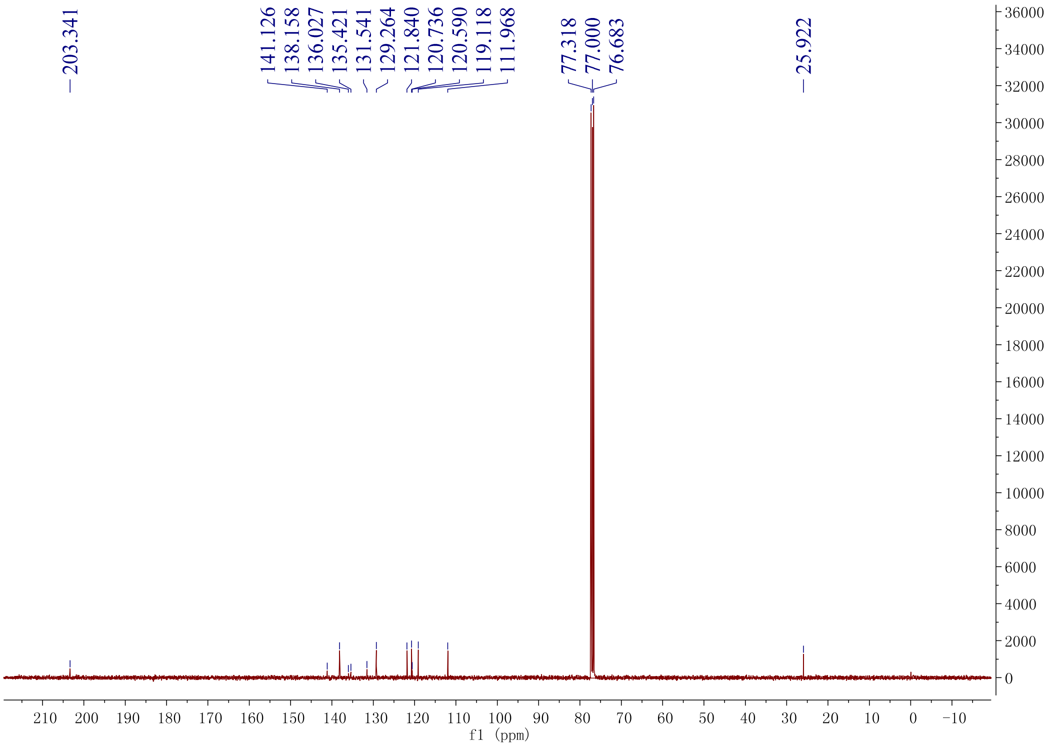

Figure S51. ^{13}C NMR spectrum of 1-(9*H*-β-carbolin-1-yl)-ethanone (**14**) in $CDCl_3$, 100 MHz.

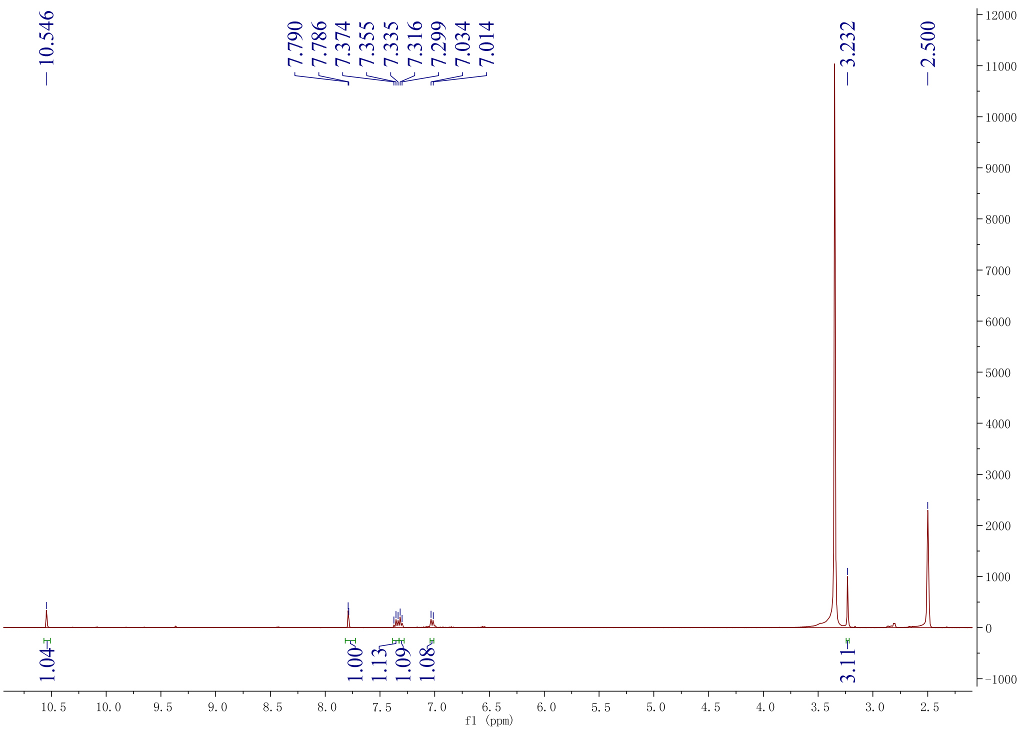

Figure S52. ^{1}H NMR spectrum of 1,2,3,4-tetrahydro-6-hydroxyl-2-methyl-l,3,4-trioxopyrazino [l,2-a]-indole (**15**) in DMSO-d_6, 400 MHz.

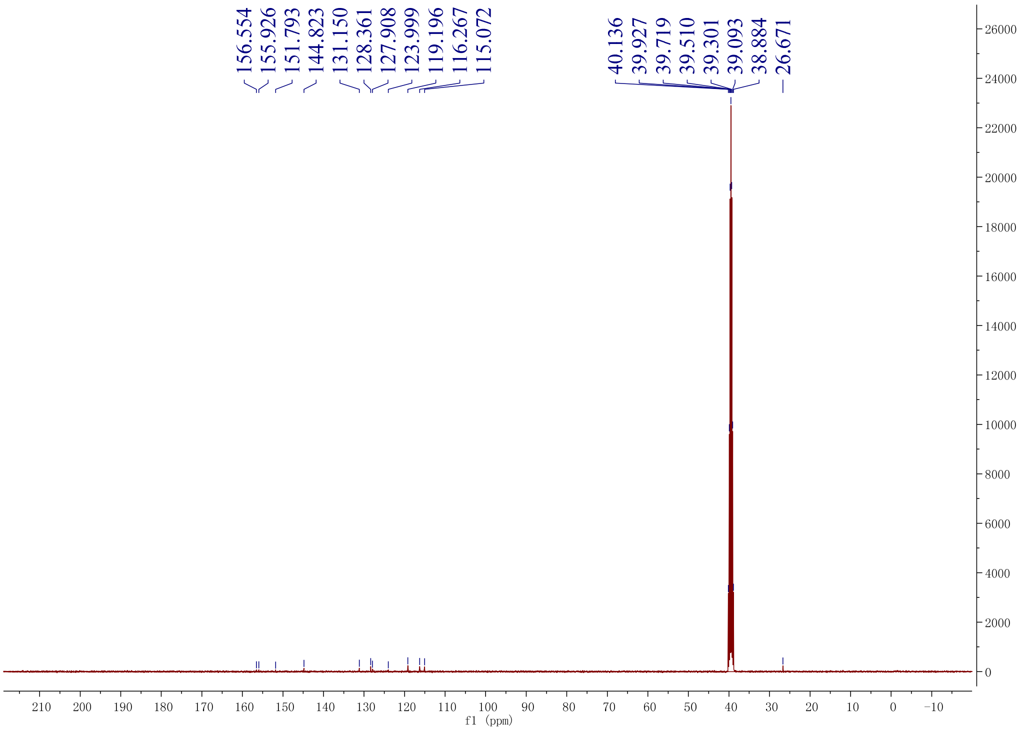

Figure S53. ^{13}C NMR spectrum of 1,2,3,4-tetrahydro-6-hydroxyl-2-methyl-l,3,4-trioxopyrazino[l,2-a]-indole (**15**) in DMSO-d_6, 100 MHz.

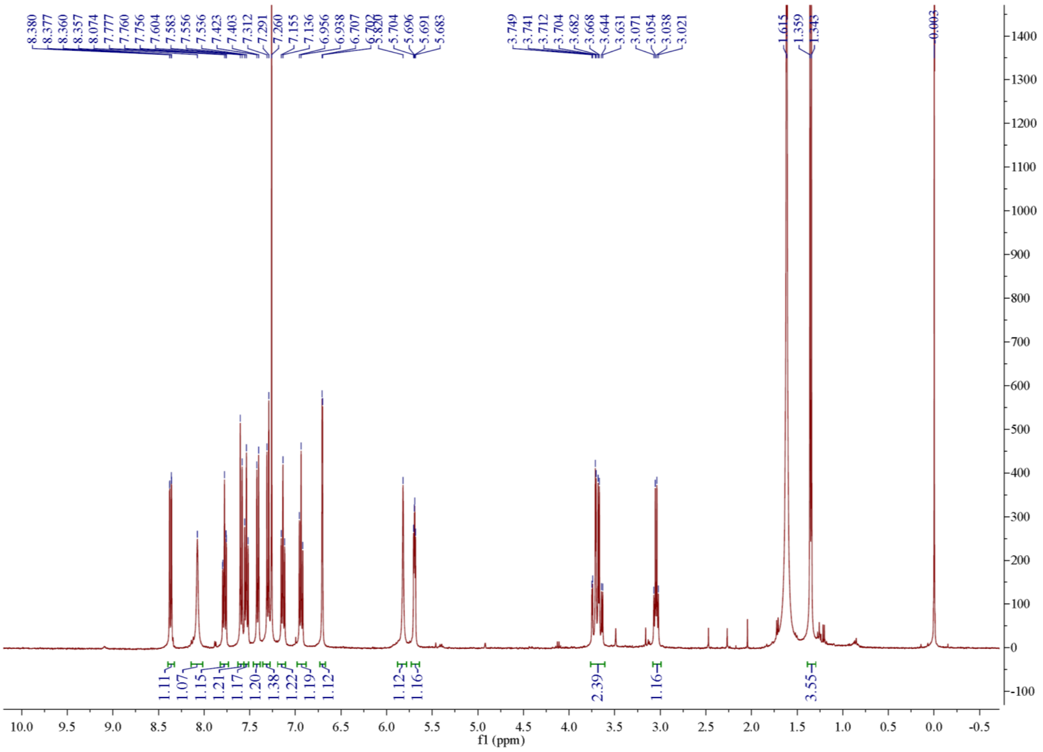

Figure S54. ^{1}H NMR spectrum of fumiquinazoline F (**16**) in $CDCl_3$, 400MHz.

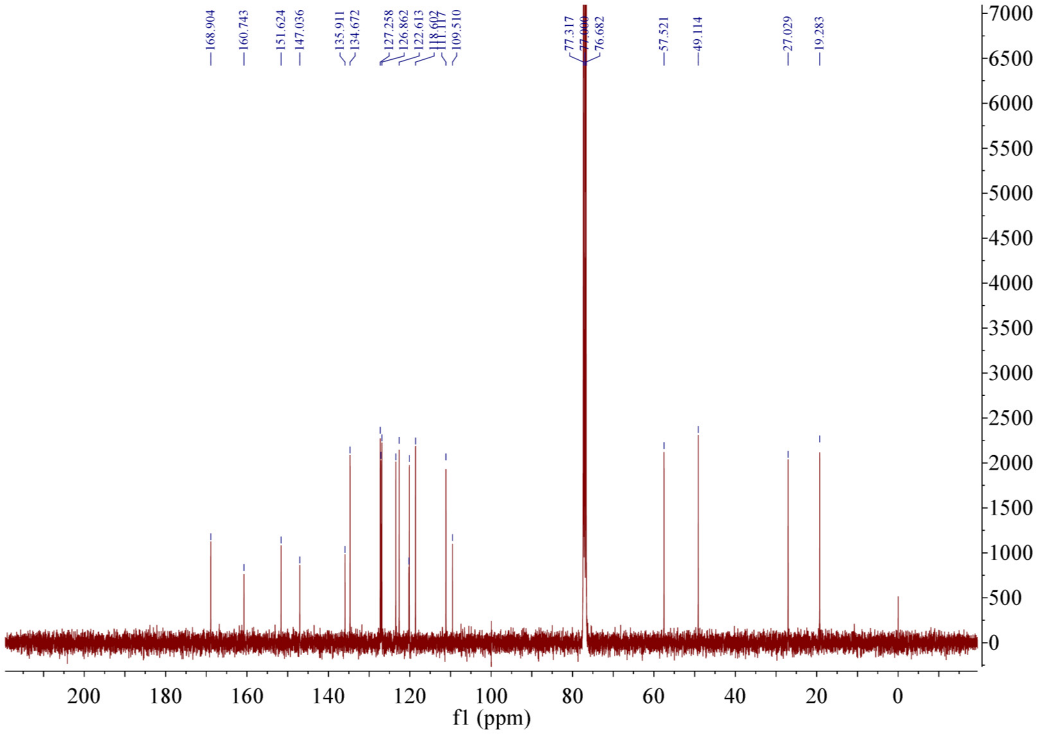

Figure S55. ^{13}C NMR spectrum of fumiquinazoline F (**16**) in $CDCl_3$, 100 MHz.

CIF of Sesquiterpene (8).

```
data_p

_audit_creation_method            SHELXL-97
_chemical_name_systematic
;
 ?
;
_chemical_name_common             Sesquiterpene
_chemical_melting_point           ?
_chemical_formula_moiety          ?
_chemical_formula_sum
 'C17 H26 O4'
_chemical_formula_weight          294.38

loop_
 _atom_type_symbol
 _atom_type_description
 _atom_type_scat_dispersion_real
 _atom_type_scat_dispersion_imag
 _atom_type_scat_source
 'C'  'C'   0.0181   0.0091
 'International Tables Vol C Tables 4.2.6.8 and 6.1.1.4'
 'H'  'H'   0.0000   0.0000
 'International Tables Vol C Tables 4.2.6.8 and 6.1.1.4'
 'O'  'O'   0.0492   0.0322
 'International Tables Vol C Tables 4.2.6.8 and 6.1.1.4'

_symmetry_cell_setting            ?
_symmetry_space_group_name_H-M    ?

loop_
 _symmetry_equiv_pos_as_xyz
 'x, y, z'
 '-x+1/2, -y, z+1/2'
 'x+1/2, -y+1/2, -z'
 '-x, y+1/2, -z+1/2'

_cell_length_a                    8.33640(10)
_cell_length_b                    9.3709(2)
_cell_length_c                    21.3150(5)
_cell_angle_alpha                 90.00
_cell_angle_beta                  90.00
```

_cell_angle_gamma 90.00
_cell_volume 1665.12(6)
_cell_formula_units_Z 4
_cell_measurement_temperature 173(2)
_cell_measurement_reflns_used ?
_cell_measurement_theta_min ?
_cell_measurement_theta_max ?

_exptl_crystal_description ?
_exptl_crystal_colour ?
_exptl_crystal_size_max ?
_exptl_crystal_size_mid ?
_exptl_crystal_size_min ?
_exptl_crystal_density_meas ?
_exptl_crystal_density_diffrn 1.174
_exptl_crystal_density_method 'not measured'
_exptl_crystal_F_000 640
_exptl_absorpt_coefficient_mu 0.663
_exptl_absorpt_correction_type ?
_exptl_absorpt_correction_T_min ?
_exptl_absorpt_correction_T_max ?
_exptl_absorpt_process_details ?

_exptl_special_details
;
 ?
;

_diffrn_ambient_temperature 173(2)
_diffrn_radiation_wavelength 1.54178
_diffrn_radiation_type CuK\a
_diffrn_radiation_source 'fine-focus sealed tube'
_diffrn_radiation_monochromator graphite
_diffrn_measurement_device_type ?
_diffrn_measurement_method ?
_diffrn_detector_area_resol_mean ?
_diffrn_standards_number ?
_diffrn_standards_interval_count ?
_diffrn_standards_interval_time ?
_diffrn_standards_decay_% ?
_diffrn_reflns_number 16100
_diffrn_reflns_av_R_equivalents 0.0405
_diffrn_reflns_av_sigmaI/netI 0.0220
_diffrn_reflns_limit_h_min -9

_diffrn_reflns_limit_h_max 9
_diffrn_reflns_limit_k_min -11
_diffrn_reflns_limit_k_max 11
_diffrn_reflns_limit_l_min -25
_diffrn_reflns_limit_l_max 25
_diffrn_reflns_theta_min 4.15
_diffrn_reflns_theta_max 66.96
_reflns_number_total 2933
_reflns_number_gt 2789
_reflns_threshold_expression >2sigma(I)

_computing_data_collection ?
_computing_cell_refinement ?
_computing_data_reduction ?
_computing_structure_solution 'SHELXS-97 (Sheldrick, 1990)'
_computing_structure_refinement 'SHELXL-97 (Sheldrick, 1997)'
_computing_molecular_graphics ?
_computing_publication_material ?

_refine_special_details
;
Refinement of F^2^ against ALL reflections. The weighted R-factor wR and
goodness of fit S are based on F^2^, conventional R-factors R are based
on F, with F set to zero for negative F^2^. The threshold expression of
F^2^ > 2sigma(F^2^) is used only for calculating R-factors(gt) etc. and is
not relevant to the choice of reflections for refinement. R-factors based
on F^2^ are statistically about twice as large as those based on F, and R-
factors based on ALL data will be even larger.
;

_refine_ls_structure_factor_coef Fsqd
_refine_ls_matrix_type full
_refine_ls_weighting_scheme calc
_refine_ls_weighting_details
'calc w=1/[\s^2^(Fo^2^)+(0.0504P)^2^+0.2215P] where P=(Fo^2^+2Fc^2^)/3'
_atom_sites_solution_primary direct
_atom_sites_solution_secondary difmap
_atom_sites_solution_hydrogens geom
_refine_ls_hydrogen_treatment mixed
_refine_ls_extinction_method none
_refine_ls_extinction_coef ?
_refine_ls_abs_structure_details
'Flack H D (1983), Acta Cryst. A39, 876-881'
_refine_ls_abs_structure_Flack -0.16(17)

_refine_ls_number_reflns 2933
_refine_ls_number_parameters 190
_refine_ls_number_restraints 0
_refine_ls_R_factor_all 0.0349
_refine_ls_R_factor_gt 0.0326
_refine_ls_wR_factor_ref 0.0860
_refine_ls_wR_factor_gt 0.0838
_refine_ls_goodness_of_fit_ref 1.048
_refine_ls_restrained_S_all 1.048
_refine_ls_shift/su_max 0.000
_refine_ls_shift/su_mean 0.000

loop_
_atom_site_label
_atom_site_type_symbol
_atom_site_fract_x
_atom_site_fract_y
_atom_site_fract_z
_atom_site_U_iso_or_equiv
_atom_site_adp_type
_atom_site_occupancy
_atom_site_symmetry_multiplicity
_atom_site_calc_flag
_atom_site_refinement_flags
_atom_site_disorder_assembly
_atom_site_disorder_group
O1 O 0.46681(12) 0.51433(11) 0.27934(5) 0.0269(2) Uani 1 1 d . . .
H1A H 0.5087 0.4958 0.2444 0.040 Uiso 1 1 calc R . .
O2 O 0.63404(12) 0.27985(11) 0.25162(5) 0.0289(2) Uani 1 1 d . . .
H2A H 0.6117 0.2003 0.2357 0.043 Uiso 1 1 calc R . .
O3 O 0.57579(12) 0.15686(11) 0.41287(5) 0.0279(2) Uani 1 1 d . . .
O4 O 0.45241(15) -0.00906(12) 0.35354(5) 0.0392(3) Uani 1 1 d . . .
C1 C 0.55912(17) 0.44826(15) 0.32845(7) 0.0234(3) Uani 1 1 d . . .
C2 C 0.56600(18) 0.28860(15) 0.31266(7) 0.0244(3) Uani 1 1 d . . .
H2B H 0.4548 0.2485 0.3120 0.029 Uiso 1 1 calc R . .
C3 C 0.66974(18) 0.20202(15) 0.35786(7) 0.0262(3) Uani 1 1 d . . .
H3A H 0.7074 0.1145 0.3354 0.031 Uiso 1 1 calc R . .
C4 C 0.81433(17) 0.27971(16) 0.38247(7) 0.0265(3) Uani 1 1 d . . .
C5 C 0.84050(18) 0.41607(16) 0.36758(7) 0.0267(3) Uani 1 1 d . . .
H5A H 0.9348 0.4598 0.3836 0.032 Uiso 1 1 calc R . .
C6 C 0.73195(17) 0.50588(16) 0.32728(7) 0.0240(3) Uani 1 1 d . . .
H6A H 0.7712 0.4966 0.2831 0.029 Uiso 1 1 calc R . .
C7 C 0.9291(2) 0.19147(18) 0.42099(8) 0.0369(4) Uani 1 1 d . . .
H7A H 1.0181 0.2516 0.4352 0.055 Uiso 1 1 calc R . .

H7B H 0.9710 0.1131 0.3953 0.055 Uiso 1 1 calc R . .
H7C H 0.8727 0.1523 0.4575 0.055 Uiso 1 1 calc R . .
C17 C 0.73678(18) 0.66772(16) 0.34400(7) 0.0269(3) Uani 1 1 d . . .
H17A H 0.6645 0.7168 0.3134 0.032 Uiso 1 1 calc R . .
C8 C 0.66293(19) 0.69028(16) 0.40876(7) 0.0304(3) Uani 1 1 d . . .
H8A H 0.7253 0.6369 0.4406 0.037 Uiso 1 1 calc R . .
H8B H 0.6665 0.7928 0.4198 0.037 Uiso 1 1 calc R . .
C9 C 0.48957(19) 0.63850(17) 0.40871(7) 0.0303(3) Uani 1 1 d . . .
H9A H 0.4269 0.6960 0.3784 0.036 Uiso 1 1 calc R . .
H9B H 0.4428 0.6536 0.4509 0.036 Uiso 1 1 calc R . .
C10 C 0.47522(17) 0.47978(16) 0.39125(7) 0.0260(3) Uani 1 1 d . . .
H10A H 0.5317 0.4236 0.4245 0.031 Uiso 1 1 calc R . .
C11 C 0.29866(18) 0.43455(18) 0.39178(9) 0.0365(4) Uani 1 1 d . . .
H11A H 0.2904 0.3334 0.3806 0.055 Uiso 1 1 calc R . .
H11B H 0.2387 0.4918 0.3613 0.055 Uiso 1 1 calc R . .
H11C H 0.2539 0.4495 0.4338 0.055 Uiso 1 1 calc R . .
C12 C 0.90253(19) 0.73034(17) 0.33435(9) 0.0362(4) Uani 1 1 d . . .
C13 C 0.9533(3) 0.7424(3) 0.26707(10) 0.0709(7) Uani 1 1 d . . .
H13A H 1.0616 0.7828 0.2649 0.106 Uiso 1 1 calc R . .
H13B H 0.8784 0.8047 0.2446 0.106 Uiso 1 1 calc R . .
H13C H 0.9530 0.6475 0.2477 0.106 Uiso 1 1 calc R . .
C14 C 0.9960(2) 0.7751(2) 0.38017(10) 0.0501(5) Uani 1 1 d . . .
H14A H 1.0976 0.8159 0.3708 0.060 Uiso 1 1 calc R . .
H14B H 0.9618 0.7667 0.4225 0.060 Uiso 1 1 calc R . .
C15 C 0.4706(2) 0.05012(16) 0.40310(7) 0.0311(3) Uani 1 1 d . . .
C16 C 0.3798(2) 0.0149(2) 0.46143(8) 0.0430(4) Uani 1 1 d . . .
H16A H 0.3042 -0.0627 0.4528 0.065 Uiso 1 1 calc R . .
H16B H 0.3207 0.0994 0.4756 0.065 Uiso 1 1 calc R . .
H16C H 0.4550 -0.0150 0.4942 0.065 Uiso 1 1 calc R . .

loop_
 _atom_site_aniso_label
 _atom_site_aniso_U_11
 _atom_site_aniso_U_22
 _atom_site_aniso_U_33
 _atom_site_aniso_U_23
 _atom_site_aniso_U_13
 _atom_site_aniso_U_12
O1 0.0275(5) 0.0251(5) 0.0279(5) 0.0039(4) -0.0023(4) 0.0025(5)
O2 0.0379(6) 0.0224(5) 0.0265(5) -0.0016(4) 0.0011(4) -0.0029(4)
O3 0.0338(5) 0.0226(5) 0.0275(5) 0.0013(4) 0.0022(4) -0.0027(4)
O4 0.0565(7) 0.0306(6) 0.0306(6) -0.0033(5) 0.0032(5) -0.0143(6)
C1 0.0222(7) 0.0219(7) 0.0262(7) 0.0024(5) -0.0021(6) 0.0012(6)
C2 0.0248(7) 0.0217(7) 0.0267(7) 0.0005(6) 0.0007(5) -0.0018(6)

C3 0.0302(8) 0.0218(7) 0.0266(7) 0.0021(6) 0.0036(6) 0.0010(6)
C4 0.0235(7) 0.0266(8) 0.0295(7) 0.0009(6) 0.0002(6) 0.0041(6)
C5 0.0205(7) 0.0293(8) 0.0302(8) -0.0011(6) 0.0023(6) 0.0014(6)
C6 0.0233(7) 0.0198(7) 0.0288(7) 0.0009(6) 0.0012(6) -0.0010(6)
C7 0.0318(8) 0.0319(9) 0.0471(10) 0.0040(7) -0.0065(7) 0.0057(7)
C17 0.0266(7) 0.0206(7) 0.0334(8) 0.0019(6) -0.0039(6) -0.0015(6)
C8 0.0352(8) 0.0223(7) 0.0338(8) -0.0041(6) -0.0049(6) 0.0020(6)
C9 0.0330(8) 0.0264(8) 0.0316(8) -0.0022(6) 0.0028(6) 0.0068(6)
C10 0.0236(7) 0.0252(7) 0.0292(7) 0.0021(6) 0.0016(6) 0.0021(6)
C11 0.0258(8) 0.0379(9) 0.0458(9) 0.0022(8) 0.0068(7) 0.0020(7)
C12 0.0299(8) 0.0233(8) 0.0553(10) 0.0026(8) -0.0021(7) -0.0046(7)
C13 0.0520(13) 0.0972(19) 0.0636(13) 0.0067(13) 0.0125(10) -0.0402(14)
C14 0.0367(9) 0.0418(10) 0.0718(13) -0.0035(10) -0.0074(9) -0.0114(8)
C15 0.0381(8) 0.0227(7) 0.0324(8) 0.0031(6) 0.0019(7) -0.0029(6)
C16 0.0562(11) 0.0379(10) 0.0350(9) -0.0003(8) 0.0095(8) -0.0143(9)

_geom_special_details
;
All esds (except the esd in the dihedral angle between two l.s. planes)
are estimated using the full covariance matrix. The cell esds are taken
into account individually in the estimation of esds in distances, angles
and torsion angles; correlations between esds in cell parameters are only
used when they are defined by crystal symmetry. An approximate (isotropic)
treatment of cell esds is used for estimating esds involving l.s. planes.
;

loop_
_geom_bond_atom_site_label_1
_geom_bond_atom_site_label_2
_geom_bond_distance
_geom_bond_site_symmetry_2
_geom_bond_publ_flag
O1 C1 1.4392(17) . ?
O1 H1A 0.8400 . ?
O2 C2 1.4216(17) . ?
O2 H2A 0.8400 . ?
O3 C15 1.3464(19) . ?
O3 C3 1.4721(17) . ?
O4 C15 1.2027(19) . ?
C1 C2 1.535(2) . ?
C1 C6 1.539(2) . ?
C1 C10 1.5389(19) . ?
C2 C3 1.528(2) . ?
C2 H2B 1.0000 . ?

C3 C4 1.503(2) . ?
C3 H3A 1.0000 . ?
C4 C5 1.335(2) . ?
C4 C7 1.507(2) . ?
C5 C6 1.505(2) . ?
C5 H5A 0.9500 . ?
C6 C17 1.558(2) . ?
C6 H6A 1.0000 . ?
C7 H7A 0.9800 . ?
C7 H7B 0.9800 . ?
C7 H7C 0.9800 . ?
C17 C12 1.515(2) . ?
C17 C8 1.526(2) . ?
C17 H17A 1.0000 . ?
C8 C9 1.525(2) . ?
C8 H8A 0.9900 . ?
C8 H8B 0.9900 . ?
C9 C10 1.538(2) . ?
C9 H9A 0.9900 . ?
C9 H9B 0.9900 . ?
C10 C11 1.532(2) . ?
C10 H10A 1.0000 . ?
C11 H11A 0.9800 . ?
C11 H11B 0.9800 . ?
C11 H11C 0.9800 . ?
C12 C14 1.318(3) . ?
C12 C13 1.499(3) . ?
C13 H13A 0.9800 . ?
C13 H13B 0.9800 . ?
C13 H13C 0.9800 . ?
C14 H14A 0.9500 . ?
C14 H14B 0.9500 . ?
C15 C16 1.492(2) . ?
C16 H16A 0.9800 . ?
C16 H16B 0.9800 . ?
C16 H16C 0.9800 . ?

loop_
 _geom_angle_atom_site_label_1
 _geom_angle_atom_site_label_2
 _geom_angle_atom_site_label_3
 _geom_angle
 _geom_angle_site_symmetry_1
 _geom_angle_site_symmetry_3

_geom_angle_publ_flag
C1 O1 H1A 109.5 . . ?
C2 O2 H2A 109.5 . . ?
C15 O3 C3 115.91(11) . . ?
O1 C1 C2 106.25(11) . . ?
O1 C1 C6 109.75(11) . . ?
C2 C1 C6 107.67(12) . . ?
O1 C1 C10 107.88(11) . . ?
C2 C1 C10 113.26(12) . . ?
C6 C1 C10 111.86(11) . . ?
O2 C2 C3 108.70(11) . . ?
O2 C2 C1 105.77(11) . . ?
C3 C2 C1 113.61(12) . . ?
O2 C2 H2B 109.5 . . ?
C3 C2 H2B 109.5 . . ?
C1 C2 H2B 109.5 . . ?
O3 C3 C4 106.74(12) . . ?
O3 C3 C2 110.72(12) . . ?
C4 C3 C2 114.64(12) . . ?
O3 C3 H3A 108.2 . . ?
C4 C3 H3A 108.2 . . ?
C2 C3 H3A 108.2 . . ?
C5 C4 C3 120.80(14) . . ?
C5 C4 C7 123.43(14) . . ?
C3 C4 C7 115.68(13) . . ?
C4 C5 C6 124.95(14) . . ?
C4 C5 H5A 117.5 . . ?
C6 C5 H5A 117.5 . . ?
C5 C6 C1 110.95(12) . . ?
C5 C6 C17 113.46(12) . . ?
C1 C6 C17 111.22(12) . . ?
C5 C6 H6A 106.9 . . ?
C1 C6 H6A 106.9 . . ?
C17 C6 H6A 106.9 . . ?
C4 C7 H7A 109.5 . . ?
C4 C7 H7B 109.5 . . ?
H7A C7 H7B 109.5 . . ?
C4 C7 H7C 109.5 . . ?
H7A C7 H7C 109.5 . . ?
H7B C7 H7C 109.5 . . ?
C12 C17 C8 115.91(13) . . ?
C12 C17 C6 111.68(12) . . ?
C8 C17 C6 109.34(12) . . ?
C12 C17 H17A 106.4 . . ?

C8 C17 H17A 106.4 . . ?
C6 C17 H17A 106.4 . . ?
C9 C8 C17 109.74(12) . . ?
C9 C8 H8A 109.7 . . ?
C17 C8 H8A 109.7 . . ?
C9 C8 H8B 109.7 . . ?
C17 C8 H8B 109.7 . . ?
H8A C8 H8B 108.2 . . ?
C8 C9 C10 112.44(12) . . ?
C8 C9 H9A 109.1 . . ?
C10 C9 H9A 109.1 . . ?
C8 C9 H9B 109.1 . . ?
C10 C9 H9B 109.1 . . ?
H9A C9 H9B 107.8 . . ?
C11 C10 C9 109.91(12) . . ?
C11 C10 C1 112.96(13) . . ?
C9 C10 C1 111.15(12) . . ?
C11 C10 H10A 107.5 . . ?
C9 C10 H10A 107.5 . . ?
C1 C10 H10A 107.5 . . ?
C10 C11 H11A 109.5 . . ?
C10 C11 H11B 109.5 . . ?
H11A C11 H11B 109.5 . . ?
C10 C11 H11C 109.5 . . ?
H11A C11 H11C 109.5 . . ?
H11B C11 H11C 109.5 . . ?
C14 C12 C13 121.19(17) . . ?
C14 C12 C17 124.19(17) . . ?
C13 C12 C17 114.60(15) . . ?
C12 C13 H13A 109.5 . . ?
C12 C13 H13B 109.5 . . ?
H13A C13 H13B 109.5 . . ?
C12 C13 H13C 109.5 . . ?
H13A C13 H13C 109.5 . . ?
H13B C13 H13C 109.5 . . ?
C12 C14 H14A 120.0 . . ?
C12 C14 H14B 120.0 . . ?
H14A C14 H14B 120.0 . . ?
O4 C15 O3 124.09(14) . . ?
O4 C15 C16 124.47(15) . . ?
O3 C15 C16 111.44(13) . . ?
C15 C16 H16A 109.5 . . ?
C15 C16 H16B 109.5 . . ?
H16A C16 H16B 109.5 . . ?

C15 C16 H16C 109.5 . . ?
H16A C16 H16C 109.5 . . ?
H16B C16 H16C 109.5 . . ?

loop_
_geom_torsion_atom_site_label_1
_geom_torsion_atom_site_label_2
_geom_torsion_atom_site_label_3
_geom_torsion_atom_site_label_4
_geom_torsion
_geom_torsion_site_symmetry_1
_geom_torsion_site_symmetry_2
_geom_torsion_site_symmetry_3
_geom_torsion_site_symmetry_4
_geom_torsion_publ_flag
O1 C1 C2 O2 56.97(14) . . . . ?
C6 C1 C2 O2 -60.56(14) . . . . ?
C10 C1 C2 O2 175.23(11) . . . . ?
O1 C1 C2 C3 176.12(11) . . . . ?
C6 C1 C2 C3 58.59(15) . . . . ?
C10 C1 C2 C3 -65.62(16) . . . . ?
C15 O3 C3 C4 -160.20(12) . . . . ?
C15 O3 C3 C2 74.41(15) . . . . ?
O2 C2 C3 O3 -156.28(11) . . . . ?
C1 C2 C3 O3 86.26(14) . . . . ?
O2 C2 C3 C4 82.90(15) . . . . ?
C1 C2 C3 C4 -34.56(18) . . . . ?
O3 C3 C4 C5 -119.42(15) . . . . ?
C2 C3 C4 C5 3.6(2) . . . . ?
O3 C3 C4 C7 63.97(16) . . . . ?
C2 C3 C4 C7 -173.04(13) . . . . ?
C3 C4 C5 C6 1.3(2) . . . . ?
C7 C4 C5 C6 177.60(14) . . . . ?
C4 C5 C6 C1 24.3(2) . . . . ?
C4 C5 C6 C17 150.37(14) . . . . ?
O1 C1 C6 C5 -166.99(11) . . . . ?
C2 C1 C6 C5 -51.74(15) . . . . ?
C10 C1 C6 C5 73.31(15) . . . . ?
O1 C1 C6 C17 65.72(14) . . . . ?
C2 C1 C6 C17 -179.04(11) . . . . ?
C10 C1 C6 C17 -53.99(15) . . . . ?
C5 C6 C17 C12 61.91(16) . . . . ?
C1 C6 C17 C12 -172.17(12) . . . . ?
C5 C6 C17 C8 -67.73(15) . . . . ?

C1 C6 C17 C8 58.19(15) . . . . ?
C12 C17 C8 C9 172.87(13) . . . . ?
C6 C17 C8 C9 -59.84(15) . . . . ?
C17 C8 C9 C10 58.82(16) . . . . ?
C8 C9 C10 C11 -179.79(13) . . . . ?
C8 C9 C10 C1 -53.98(16) . . . . ?
O1 C1 C10 C11 54.48(15) . . . . ?
C2 C1 C10 C11 -62.83(16) . . . . ?
C6 C1 C10 C11 175.28(12) . . . . ?
O1 C1 C10 C9 -69.61(14) . . . . ?
C2 C1 C10 C9 173.08(12) . . . . ?
C6 C1 C10 C9 51.19(15) . . . . ?
C8 C17 C12 C14 14.2(2) . . . . ?
C6 C17 C12 C14 -111.93(19) . . . . ?
C8 C17 C12 C13 -164.11(18) . . . . ?
C6 C17 C12 C13 69.8(2) . . . . ?
C3 O3 C15 O4 1.1(2) . . . . ?
C3 O3 C15 C16 -178.88(13) . . . . ?

loop_
 _geom_hbond_atom_site_label_D
 _geom_hbond_atom_site_label_H
 _geom_hbond_atom_site_label_A
 _geom_hbond_distance_DH
 _geom_hbond_distance_HA
 _geom_hbond_distance_DA
 _geom_hbond_angle_DHA
 _geom_hbond_site_symmetry_A
O1 H1A O4 0.84 2.11 2.9194(15) 160.6 4_655
O2 H2A O1 0.84 1.89 2.7080(14) 164.5 4_645
C2 H2B O4 1.00 2.57 3.0718(18) 110.8 .
C10 H10A O3 1.00 2.54 3.1737(18) 121.2 .

_diffrn_measured_fraction_theta_max 0.996
_diffrn_reflns_theta_full 66.96
_diffrn_measured_fraction_theta_full 0.996
_refine_diff_density_max 0.155
_refine_diff_density_min -0.167
_refine_diff_density_rms 0.035
